# *Burkholderia pseudomallei* BopE suppresses the Rab32-dependent defense pathway to promote its intracellular replication and virulence

**DOI:** 10.1128/msphere.00453-24

**Published:** 2024-10-21

**Authors:** Chenglong Rao, Ziyuan Zhang, Jianpeng Qiao, Dongqi Nan, Pan Wu, Liting Wang, Changhao Yao, Senquan Zheng, Jinzhu Huang, Yaling Liao, Wenzheng Liu, Zhiqiang Hu, Shiwei Wang, Yuan Wen, Jingmin Yan, Xuhu Mao, Qian Li

**Affiliations:** 1 Department of Clinical Microbiology and Immunology, College of Pharmacy and Medical Laboratory, Army Medical University (Third Military Medical University), Chongqing, China; 2 Second Brigate of Student, College of Basic Medical Sciences, Army Medical University (Third Military Medical University), Chongqing, China; 3 Biomedical Analysis Center, Army Medical University (Third Military Medical University), Chongqing, China; The University of Texas Medical Branch at Galveston, Galveston, Texas, USA

**Keywords:** *Burkholderia pseudomallei*, Rab32-dependent defense pathway, T3SS, BopE, intracellular survival

## Abstract

Melioidosis is a serious infectious disease caused by the Gram-negative bacterium *Burkholderia pseudomallei*. Recently, Rab32-dependent immune vesicles emerge as a critical defense pathway to restrict the intracellular *B. pseudomallei*. However, *B. pseudomallei* can evade host immune vesicles and survive in the cytoplasm, although this mechanism is not well understood. In this study, we found Rab32-dependent vesicles could effectively combat *B. pseudomallei* infection, but not all intracellular *B. pseudomallei* were encapsulated in Rab32-positive vesicles. To explore how *B. pseudomallei* counteracted the Rab32-dependent defense pathway, transcriptomic profiling of *B. pseudomallei* was performed to characterize the response dynamics during infection. We found that the type III secretion system of *B. pseudomallei* was activated, and a variety of effector proteins were highly upregulated. Among them, BopE, BprD, and BipC were shown to interact with Rab32. Interestingly, BopE directly interacts with host Rab32, potentially suppressing Rab32 function by interfering with nucleotide exchange, which in turn restricts the recruitment of Rab32 to bacterial-containing vesicles. Knocking out of BopE can increase the proportion of Rab32-positive vesicles, suppressing the intracellular replication and virulence of *B. pseudomallei*. Collectively, our findings have demonstrated that BopE may be an important effector for *B. pseudomallei* to evade from the Rab32-dependent killing vesicles into the cytosol for survival and replication. Therefore, a deeper understanding of the interaction between BopE and the host Rab32-dependent restriction pathway may provide an effective therapeutic strategy for the elimination of intracellular *B. pseudomallei*.

**IMPORTANCE:**

*B. pseudomallei* is facultative intracellular bacterium that has evolved numerous strategies to evade host immune vesicles and survive in the cytoplasm. Rab32-dependent vesicles are one of these immune vesicles, but the mechanism by which *B. pseudomallei* escape Rab32-dependent vesicles remains elusive. Here, we find *B. pseudomallei* infection leading the activation of the type III secretion system (T3SS-3) and increasing the expression of various effectors. Specifically, we identify that BopE, an effector secreted by T3SS-3, triggers vesicle escape to promote *B. pseudomallei* pathogenicity and survival. Mechanistically, BopE suppresses the activation of Rab32 by interfering with nucleotide exchange, ultimately triggering vesicle escape and intracellular survival. We also find knocking out the *bopE* gene can increase the proportion of Rab32-positive vesicles that trap *B. pseudomallei*, dampening the survival of *B. pseudomallei* both *in vitro* and *in vivo*. Taken together, our findings provide insights into the molecular mechanisms of pathogen effector-induced vesicle escape, indicating a potential melioidosis treatment via blocking *B. pseudomallei* BopE-host Rab32 interaction.

## INTRODUCTION

Melioidosis, a tropical illness, which can be acquired from the environment, is caused by *Burkholderia pseudomallei* infection. Currently, community-acquired clinical cases of melioidosis mainly result from injurious inoculation, ingestion, and inhalation of aerosolized bacteria (1, 2). It is estimated that there were currently 165,000 annual cases of melioidosis and 89,000 deaths worldwide (3). The clinical symptoms of melioidosis are diverse, including asymptomatic infection, chronic localized pathology, acute sepsis, or a latent infection that can reactivate decades later (4). Owing to the serious biological hazard, *B. pseudomallei*, the causative agent, has been classified as a tier-1 select agent by the Center for Disease Control (CDC) of the United States (2). Melioidosis is highly endemic in Southeast Asia and northern Australia, and the endemic regions are believed to be expanding globally, such as in southeast Queensland, Australia, and regions of the southern United States, with a variety of factors including climate change, environmental disruption, and increased human activity (5). Although melioidosis is of growing concern, the therapeutic resources are extremely limited (6). Elucidating the pathogenic mechanism of *B. pseudomallei* infection is essential for clinical diagnosis, treatment, and prevention of melioidosis.

*B. pseudomallei* can invade a broad range of host cells, particularly through a process where it is phagocytosed into phagosomes by macrophages. And following internalization, *B. pseudomallei* is encapsulated in macrophage endocytic vesicles. The maturation of phagocytic vesicle transport is crucial for the clearance of pathogens within macrophages (7). It has established the role of Rab32 in the regulation of phagosome maturation and intracellular survival of various pathogens, such as *Salmonella typhi* (8) and *L. monocytogenes* (9). Functionally, Rab32 participates in the regulation of the cyclic transition of vesicles by facilitating the cyclic transition from an inactive state (bound to GDP) to an active state (bound to GTP). The activation and recruitment of Rab32 to bacterial-containing vesicles lead to the degradation of engulfed bacteria (9, 10). Rab32 is also able to promote the transport of the antibacterial agent itaconate, to vesicles resulting in engulfed bacterial elimination (11–13). Notably, our previous research has demonstrated that Rab32 is recruited to *B. pseudomallei*-containing phagosome vesicle (BCV) and promotes the fusion of BCV with lysosomes and activates lysosomal acid hydrolase, thus limiting the intracellular growth of *B. pseudomallei* in murine macrophages (14). However, growing evidence suggests *B. pseudomallei* has evolved numerous strategies to evade BCV, by escaping in the cytoplasm (15–17). The mechanism by which *B. pseudomallei* escapes from Rab32-dependent vesicles remains to be elucidated.

The type III secretion system (T3SS) is a highly recognized mechanism employed by pathogens to secrete a variety of effector proteins into the host cell, which enables bacteria to escape from endocytic vesicles, promoting their intracellular survival and pathogenesis (18). *B. pseudomallei* encodes for three distinct T3SSs (T3SS-1, *BPSS1390-1408*; T3SS-2, *BPSS1613-1629*; and T3SS-3, *BPSS1520-1554*). Among these, T3SS-3 is particularly well characterized for its role in the intracellular virulence and manipulation of host cell processes (19–21). Inactivating T3SS-3 in *B. pseudomallei* effectively prevents the pathogen from escaping the BCVs, which can be achieved either by altering the apparatus proteins of T3SS-3, such as BsaZ, BsaQ, and BipD (20, 22), or by employing small molecule inhibitors that target the ATPase activity of BsaS, a component of T3SS-3 (23). Notably, it has been reported that *Salmonella typhi* delivers two T3SS effectors, SopD2 and GtgE, to disrupt host immune response by specifically interacting with Rab32 and deactivating Rab32 (8, 24). Therefore, T3SS-3 in *B. pseudomallei* may be the significant strategy that interferes with Rab32 function to facilitate *B. pseudomallei* escape from the Rab32-positive vesicles.

In our study, we found that a proportion of *B. pseudomallei* was able to escape from Rab32-positive vesicles and replicate within the cytoplasm. Transcriptomic analysis found that the T3SS-3 of *B. pseudomallei* was activated and a broad range of its effector proteins were highly upregulated during intracellular infection. Interestingly, the effector proteins BipC, BopE, and BprD were shown to interact with Rab32. In particular, BopE can prevent Rab32 recruitment to BCVs, facilitating *B. pseudomallei* escape into the cytoplasm for replicating, which may be mediated by interfering with Rab32 at the level of nucleotide exchange. Furthermore, we found that BopE-deficient strains not only showed a significant reduction in the number of Rab32-positive vesicles in RAW264.7 cells but also exhibited a decrease in virulence in mice, suggesting that BopE plays a crucial role in modulating *B. pseudomallei*-host interactions. These findings may shed a light on the pathogenic mechanism by which *B. pseudomallei* manipulates Rab32-dependent defense pathways for its intracellular replication.

## RESULTS

### *B. pseudomallei* is able to escape Rab32-positive vesicles and replicate intracellularly

**Fig 1:**
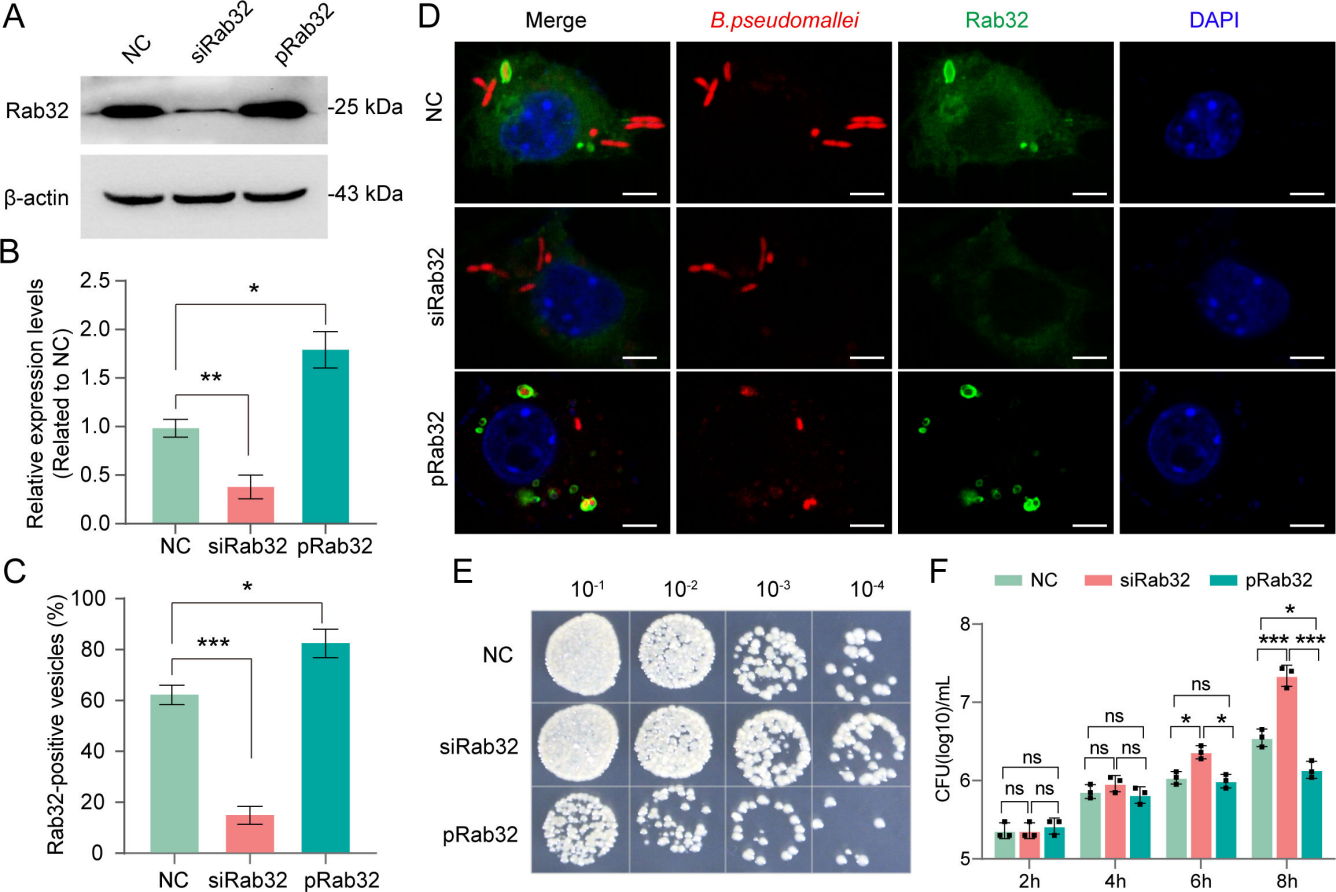
*B. pseudomallei* is able to escape Rab32 vesicles and replicate intracellularly. (**A**) Western blot analysis of Rab32 in RAW264.7 cells. Rab32 was detected by its antibody; cells without treatment were used as the NC. (**B**) The relative expression levels of Rab32 were determined by quantifying band intensities presented in panel A. The ratio of the intensities of all Rab32 bands to that of β-actin has been calculated and adjusted for normalization against the group of NC. (**C**) Quantification of Rab32 co-localized with *B. pseudomallei*. Fifty infected RAW264.7 cells in 10 fields of view were counted in each group after 2 hours of infection from three independent experiments. All assessments were performed in a blinded manner. *P < 0.05, **P < 0.01, and ***P < 0.001; ns, not significant (one-way ANOVA). (**D**) Representative immunofluorescence images of co-localization of Rab32 and *B. pseudomallei* in RAW264.7 cells after 2 hours of infection by confocal microscopy. RAW264.7 cells were infected with *B. pseudomallei* (MOI = 10:1) and imaged at the indicated time points of 2 hpi. Rab32 was stained with an anti-Rab32 antibody (green); *B. pseudomallei* was stained with anti-*B*. *pseudomallei* antibody (red). Scale bar, 5 µm. (**E and F**) Intracellular growth of *B. pseudomallei* was detected by CFU assay. Plate count of *B. pseudomallei* at 8 hpi (**E**). Intracellular *B. pseudomallei* burdens in RAW264.7 cells at the indicated times after infection (**F**). NC is negative control group, siRab32 is Rab32-siRNA group, and pRab32 is Rab32 overexpression group. The graphed data represent mean ± SD of three biological replicates. *P < 0.05, **P < 0.01, and ***P < 0.001; ns, not significant (one-way ANOVA).

Our previous research has established that the Rab32-dependent membrane vesicle traffic pathway in macrophages could be a host strategy for the restriction of phagocytosed *B. pseudomallei* (14). This hypothesis was further substantiated by our observation of a pronounced elevation in Rab32 expression levels following *B. pseudomallei* infection (Fig. S1). To investigate the effect of Rab32 on the elimination of *B. pseudomallei*, we overexpressed Rab32 with pcDNA4-Rab32 or knocked down Rab32 with siRNA in RAW264.7 cells (Fig. 1A and B). Subsequently, these cells were infected with *B. pseudomallei* at an MOI of 10. It was observed that the intracellular *B. pseudomallei* were efficiently co-localized with Rab32. Interestingly, we found that the proportion of Rab32-positive vesicles in negative control (NC) cells was 62.26% ± 2.92%, which significantly decreased to 15.4% ± 2.65% (*t* = 20.52, *P* < 0.0001) after knocking down Rab32 in RAW264.7 cells. Conversely, when Rab32 was overexpressed, the proportion was significantly increased to 83.26% ± 4.80% (*t* = 6.47, *P* = 0.0029), as seen in Fig. 1C. However, not all intracellular bacteria were contained in Rab32-positive vesicles. Visibly, Rab32-positive vesicle-free *B. pseudomallei* were ~37.74% in the NC group, while the percentage was decreased to ~16.74% when overexpressing Rab32 in RAW264.7 cells (Fig. 1D). This observation was also recorded by live cell video microscopy (Movie S1). These findings suggest that while Rab32-dependent defense vesicles have the ability to encapsulate *B. pseudomallei*, the bacteria can still manage to escape from these vesicular enclosures.

To further estimate the intracellular survival of *B. pseudomallei*, bacterial survival assays were conducted at different infection periods. We found the expression level of Rab32 did not affect the bacterial load of *B. pseudomallei* at the early stage, as there was no statistically significant difference in the amount of intracellular *B. pseudomallei* at 2 hours post-infection. But after 8 hours of infection, the intracellular survival of *B. pseudomallei* was significantly increased to 7.34 ± 0.13 log_10_CFU/mL in Rab32 knockdown cells, compared with 6.55 ± 0.11 log_10_CFU/mL in the NC group (*P* = 0.0004), while it significantly decreased to 6.14 ± 0.09 log_10_CFU/mL when Rab32 overexpressed (*P* = 0.013), and siRab32 group rose more than 10 times relative to the pRab32 group (*P* < 0.0001), shown in Fig. 1E and F. These indicate that Rab32 overexpression can efficiently suppress *B. pseudomallei* intracellular replication. Collectively, our findings indicate that Rab32 can be immediately recruited to the BCV membrane, thereby limiting *B. pseudomallei* intracellular replication. In response, *B. pseudomallei* is presumed to have evolved some specific strategies to avoid containment within Rab32-positive vesicles for intracellular replication.

### The T3SS-3 of *B. pseudomallei* is activated during infection

**Fig 2:**
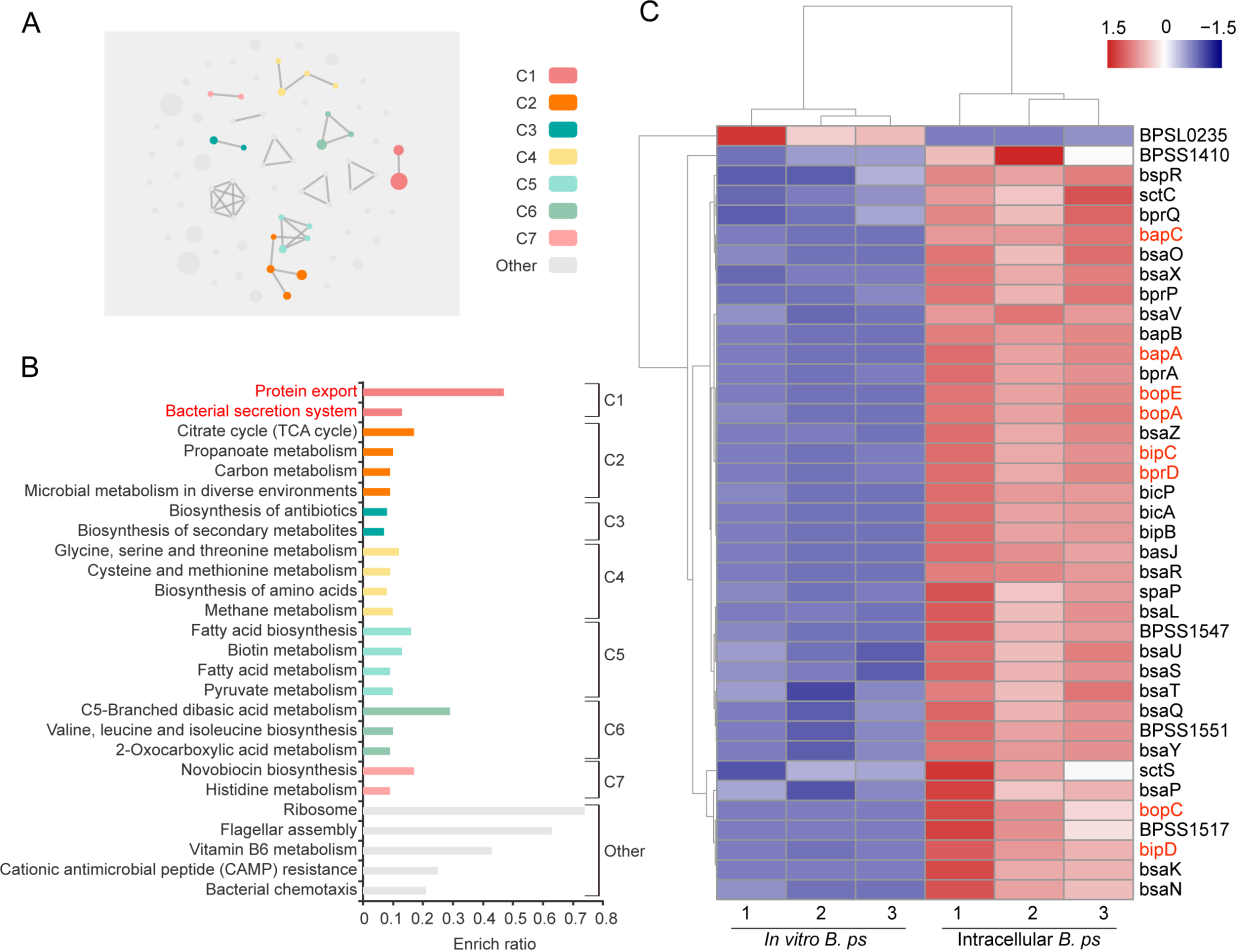
Transcriptomic analyses reveals type III secretion system was activated during *B. pseudomallei* infection. (**A**) Enriched terms of the DEGs visualized in cirFunMap from the KOBAS tool. Each node represents an enriched term, and the node color represents different clusters for enriched pathways; the node size represents six levels of enriched *P* value, node sizes from small to large: 0.05 ≤ *P* value ≤ 1, 0.01 ≤ *P* value < 0.05, 0.00 ≤ *P* value < 0.01, 0.0001 ≤ *P* value < 0.001, 1*e*^−10^ ≤ *P* value < 0.0001, 0 ≤ *P* value < 1*e*^−10^. (**B**) Enriched terms of DEGs visualized in cirFunMap. Each row represents an enriched pathway, and the length of the bar represents the enrich ratio, which is calculated as “mapped gene number”/“background gene number.” The color of the bar is the same as the color in the circular network above (**A**), which represents different clusters. For each cluster, if there are more than five terms, top five with the highest enrich ratio were displayed. (**C**) The heatmap of the DEGs enriched in T3SS-3 with a cutoff threshold of 2.0-fold difference between *in vitro B. ps* and intracellular *B. ps*. T3SS-3 effector proteins were marked as red.

In order to investigate the strategies employed by *B. pseudomallei* to evade Rab32-positive vesicles, we performed comparative transcriptomic analyses between *B. pseudomallei* cultured *in vitro* (*in vitro B. ps*) and during intracellular infection (intracellular *B. ps*) to obtain a global view of virulence factors involved in *B. pseudomallei* infection. Applying a cut-off level of twofold change and a *P* value < 0.05 on the mRNA level, a total of 1,137 differentially expressed genes (DEGs) were detected (Table S3). In these genes, 771 DEGs (67.81%) of *B. pseudomallei* were upregulated and 366 genes (32.19%) were downregulated (Fig. S2). Pathway enrichment analysis demonstrates that protein export and the bacterial secretion system are enriched during *B. pseudomallei* infection (Fig. 2A and B). Interestingly, we found that 97.43% of DEGs (38/39) enriched in the type III secretion system (T3SS) was upregulated, which included T3SS-3 regulators (e.g., BspR, BprP, and BsaN), chaperones (e.g., BicA, BicP, and BapB), and apparatus components (e.g., BsaP, BsaZ, and BsaQ). Of particular interest were eight T3SS-3 effectors, including BipC, BipD, BopC, BprD, BopE, BapC, BopA, and BapA, which were emphasized with red highlighting in Fig. 2C. Additionally, a two-component system, such as VirAG (25), and ribosome and flagellar assembly, such as FliG (26), have also shown significant enrichment. Our transcriptomic data suggest that *B. pseudomallei* T3SS-3 is activated during the infection process.

### T3SS-activated *B. pseudomallei* highly expressed effector proteins during infection

**Fig 3:**
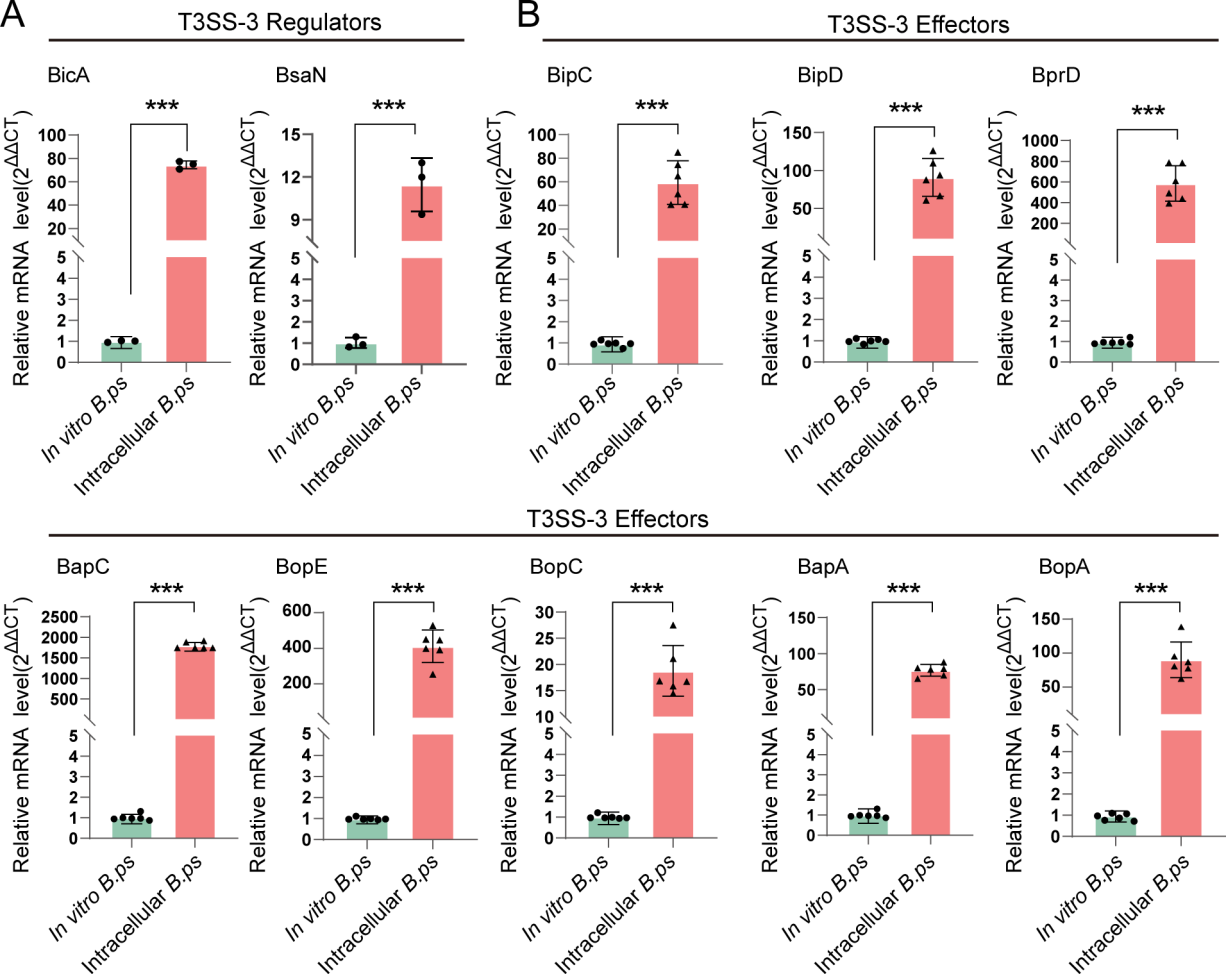
*B. pseudomallei* highly expresses T3SS-3 effector proteins during infection. (**A**) Quantification of the expression levels of BicA and BsaN in intracellular *B. pseudomallei* by RT-qPCR analysis. *B. pseudomallei* that infected RAW264.7 cells for 4 hpi serve as the intracellular *B. ps* group, while *B. pseudomallei* cultured *in vitro* (*in vitro B. ps*) is used as control group. (**B**) The mRNA levels of *B. pseudomallei* T3SS-3 effectors were analyzed by RT-qPCR. *rpoB* of *B. pseudomallei* was used as a reference gene. All data are presented as mean ± SD, and from at least three independent experiments, ***P < 0.001 (Student’s *t*-test).

The T3SS is essential for the intracellular survival strategies of *B. pseudomallei*, with its effectors commonly deployed to subvert host cell immune responses (27). BsaN, together with the chaperone BicA, acts as a central regulator that plays an essential role in activating the functionality of T3SS-3 in *B. pseudomallei* (28, 29). To ascertain the role of the T3SS-3 during *B. pseudomallei* infection, the transcriptional levels of BsaN and BicA were detected by reverse transcription-quantitative polymerase chain reaction (RT-qPCR), and the results demonstrated a marked increase in the expression levels of these two genes (Fig. 3A). This upregulation is consistent with the RNA sequencing data and points to a probable active role of the T3SS-3 in facilitating the infection process. We further detected the expression dynamics of T3SS-3 effectors among the DEGs and found a significant upregulation of eight effectors during the infection (Fig. 3B). These results suggest that T3SS-3 is activated during *B. pseudomallei* infection, and the eight upregulated effectors may be crucial for *B. pseudomallei* to persist within host cells.

### *B. pseudomallei* T3SS effector protein BopE interacts with Rab32

**Fig 4:**
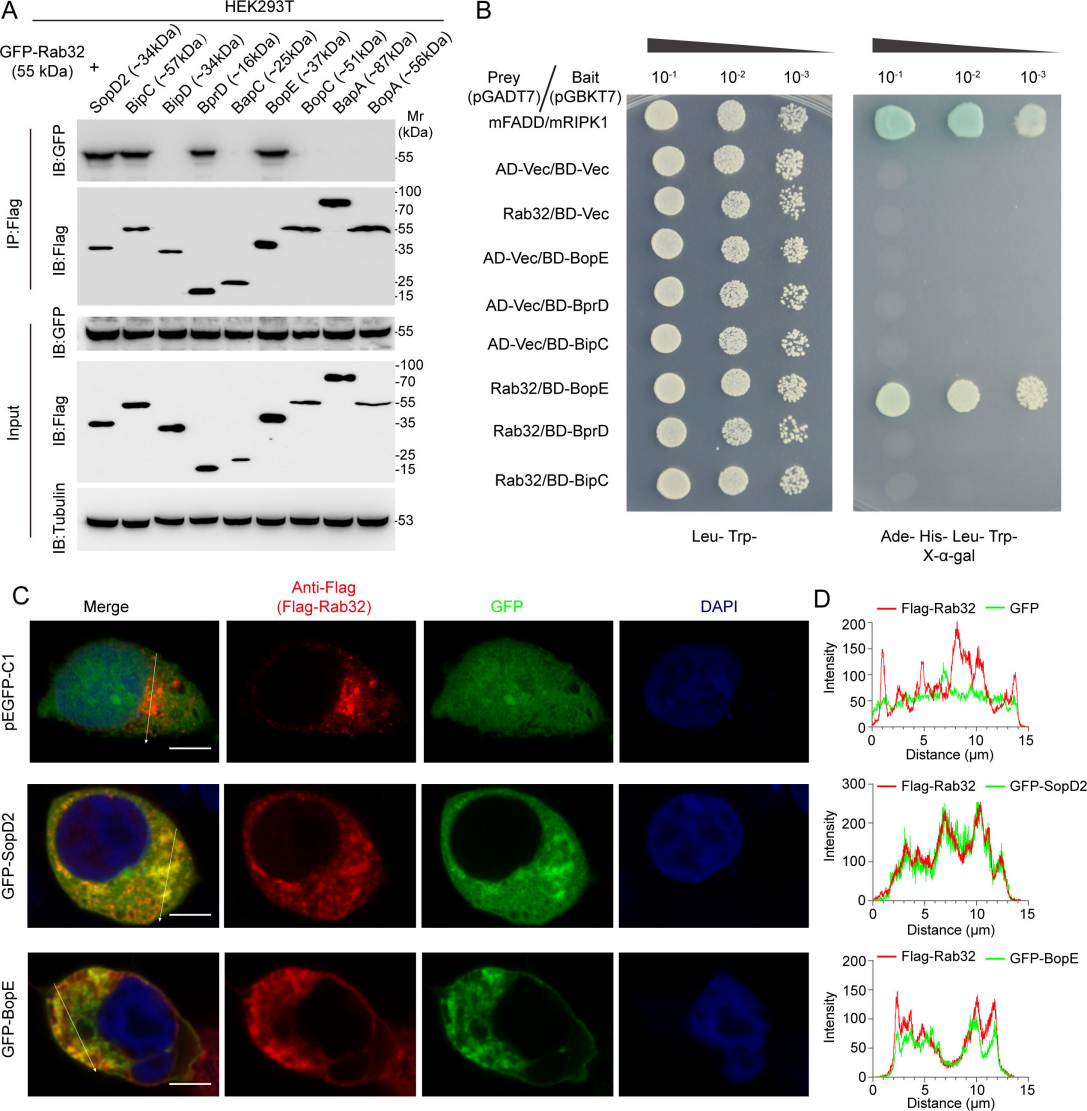
*B. pseudomallei* T3SS effector protein BopE directly interacted with Rab32. (**A**) Co-immunoprecipitation assays of Rab32 and *B. pseudomallei* T3SS effector proteins. The molecular mass of FLAG-tagged T3SS effector proteins and that of GFP-tagged Rab32 are denoted within corresponding parentheses. “Input” refers to the total protein lysate, while “IP:Flag” indicates the immunoprecipitated protein using an anti-Flag antibody. “IB:GFP/Flag/Tubulin” denotes the immunoblotting with antibodies against GFP (to detect pEGFP-Rab32), Flag (to detect the T3SS effector proteins), and tubulin (as a loading control). (**B**) The interaction assay between *B. pseudomallei* T3SS effector proteins and Rab32 using yeast two-hybrid system. The transformants were plated onto low-stringency selection plates (left) to verify the successful integration of the plasmids and high-stringency selection plates (right) to detect any potential interactions between the T3SS effectors and Rab32. (**C**) Representative immunofluorescence images of co-localization of Rab32 and BopE in HEK293T cells. GFP-BopE and Flag-Rab32 were co-expressed in HEK293T cells. Rab32 was stained with anti-Flag (red), BopE, or SopD2 tagged with GFP (green). Images show maximum-intensity projections of confocal Z-stacks. The empty vector of pEGFP-C1 was used as a negative control, and SopD2 was used as a positive control. Scale bars, 5 µm. (**D**) Confocal microscopy of BopE and Rab32 in panel **C** with fluorescence intensity plotted along the arrows.

Heterologous expression of prokaryotic proteins within eukaryotic cellular systems is a widely utilized approach across the fields of cell biology and microbiological research (30, 31). To identify potential effectors that interact with Rab32, we developed an array of expression plasmids with a Flag tag by cloning eight highly expressed *B. pseudomallei* effector genes into the pcDNA4-Basic vector. These constructs were confirmed to be correct by sequencing and enzymatic digestion, followed by expression verification (Fig. S3). We subsequently executed co-immunoprecipitation assays utilizing HEK293 cells, which were co-transfected with effector-expressing plasmids and pEGFP-Rab32, a vector encoding Rab32 tagged with GFP. SopD2, identified as a GTPase-activating protein (GAP) for Rab32 in *Salmonella* Typhimurium (24), served as a positive control in our experiments. Notably, we found that BprD, BipC, and BopE were capable of interacting with Rab32, as demonstrated in Fig. 4A.

To further substantiate the interactions with Rab32, we employed the yeast two-hybrid system, a well-established method for assessing protein-protein interactions, known as the Y2H assay. We found that only the Y2HGold strain, co-transformed with the pGBKT7-BopE and pGADT7-Rab32 expression vectors, exhibited growth under both low-stringency conditions (Leu^−^ and Trp^−^) and high-stringency conditions (Ade^−^, His^−^, Leu^−^, and Trp^−^), which indicates BopE can interact with Rab32 *in vivo* (Fig. 4B). Confocal microscopy analysis also confirmed significant co-localization of BopE with Rab32 (Fig. 4C and D). Collectively, our findings provide evidence supporting the interaction between BopE and Rab32 *in vivo*.

### BopE suppresses Rab32 in a manner unrelated to its GEF activity

**Fig 5:**
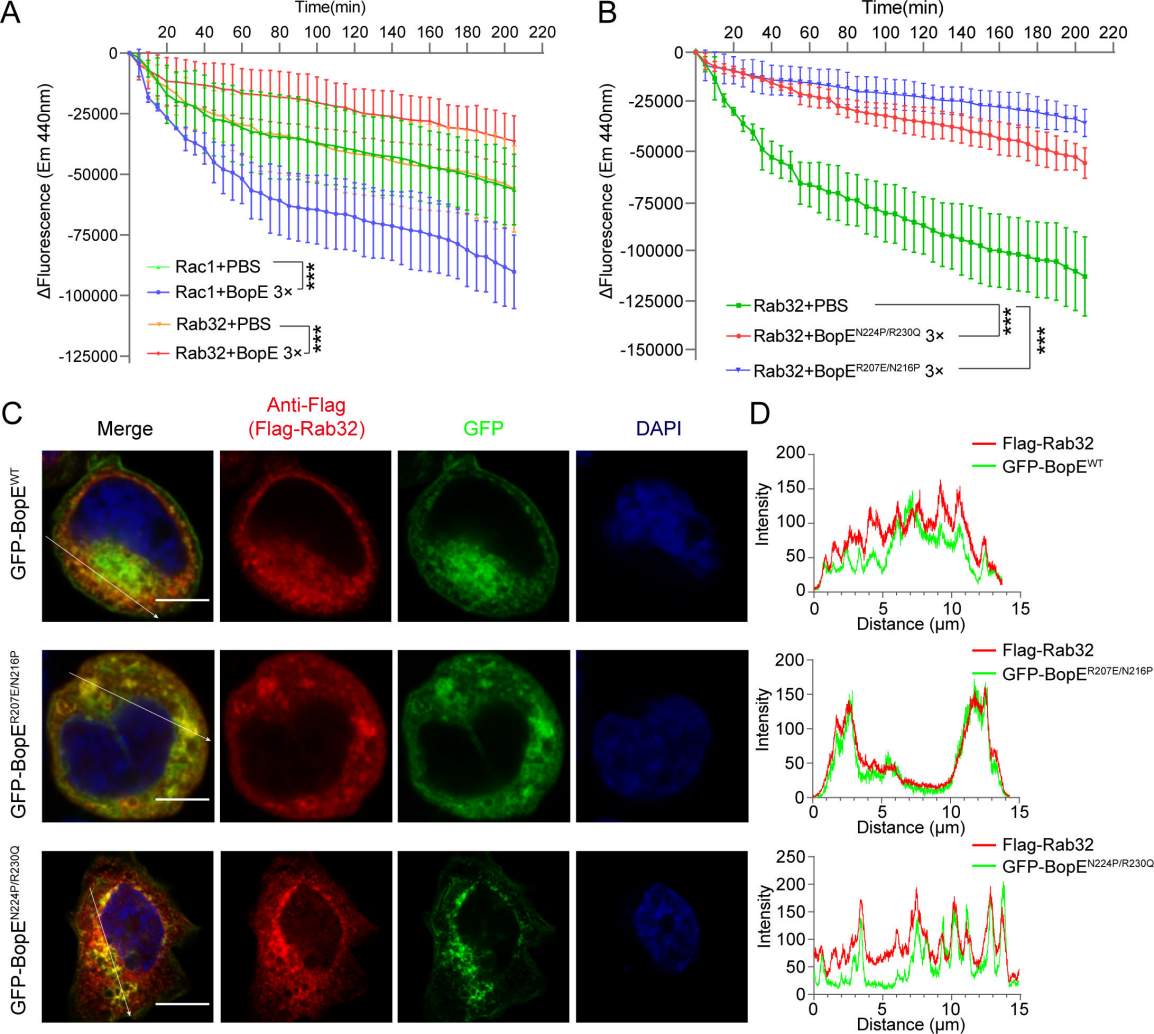
BopE interferes with the nucleotide exchange process of Rab32. (**A**) Nucleotide exchange assay for Rab32. ΔFluorescence is equal to fluorescence intensity measured at each specified time point minus the baseline fluorescence intensity recorded at the onset of the experiment. All dots are presented as mean ± SD and derived from three independent experiments. ***P < 0.001 (two-way ANOVA with Tukey’s method). (**B**) BopE inhibits the activation of Rab32 independent of the GEF activity. The mutant BopE^N224P/R230Q^ exhibits high catalytic activity, while the mutant BopE^R207E/N216P^ has lost its catalytic activity. Fluorescence spectrometry assay data were showed mean ± SD from three independent experiments. ***P < 0.001 (two-way ANOVA with Tukey’s method). (**C**) BopE interacts with Rab32 independent of its GEF activity. Representative immunofluorescence images of co-localization of Rab32 and the GEF activity mutants of BopE in HEK293T cells. Rab32 was stained with an anti-Flag antibody (red), BopE, or its mutants tagged with GFP (green). Scale bar, 5 µm. (**D**) Confocal microscopy of BopE and Rab32 in panel **C** with fluorescence intensity plotted along the arrows.

A previous study suggested BopE can activate small GTPases Rac1 and Cdc42 through its guanine nucleotide exchange factor (GEF) function (32). We then hypothesis that BopE can also interfere in the activation of Rab32. Surprisingly, through an *in vitro* nucleotide exchange assay using N-methylanthraniloyl-guanosine diphosphate (Mant-GDP) (33), we found BopE can suppress the GTP exchange activation for Rab32 in a dose-dependent manner, while it can activate Rac1 as previously described (Fig. 5A; Fig. S4B). It suggested BopE has an additional function to suppress Rab32 in a manner unrelated to its GEF activity. To further investigate this hypothesis, we introduce a catalytically inactive mutant (BopE^R207E/N216P^) and a highly active mutant (BopE^N224P/R230Q^) as previously described (34). However, neither those two mutants can reverse the suppression of BopE to Rab32 (Fig. 5B). The T3SS effector SopD from *Salmonella Typhimurium* has been shown to activate Rab8 through direct interaction and is unrelated to its GAP activity (35). Therefore, we examine the interaction between BopE and its mutants with Rab32. As show in Fig. 5C and D, interference with GEF activity of BopE does not alter its co-localization with Rab32. Taken together, these results suggest that BopE suppresses Rab32 via a mechanism independent of its GEF function.

### BopE targets the Rab32 pathogen-restriction pathway to enhance *B. pseudomallei* intracellular replication and virulence

**Fig 6:**
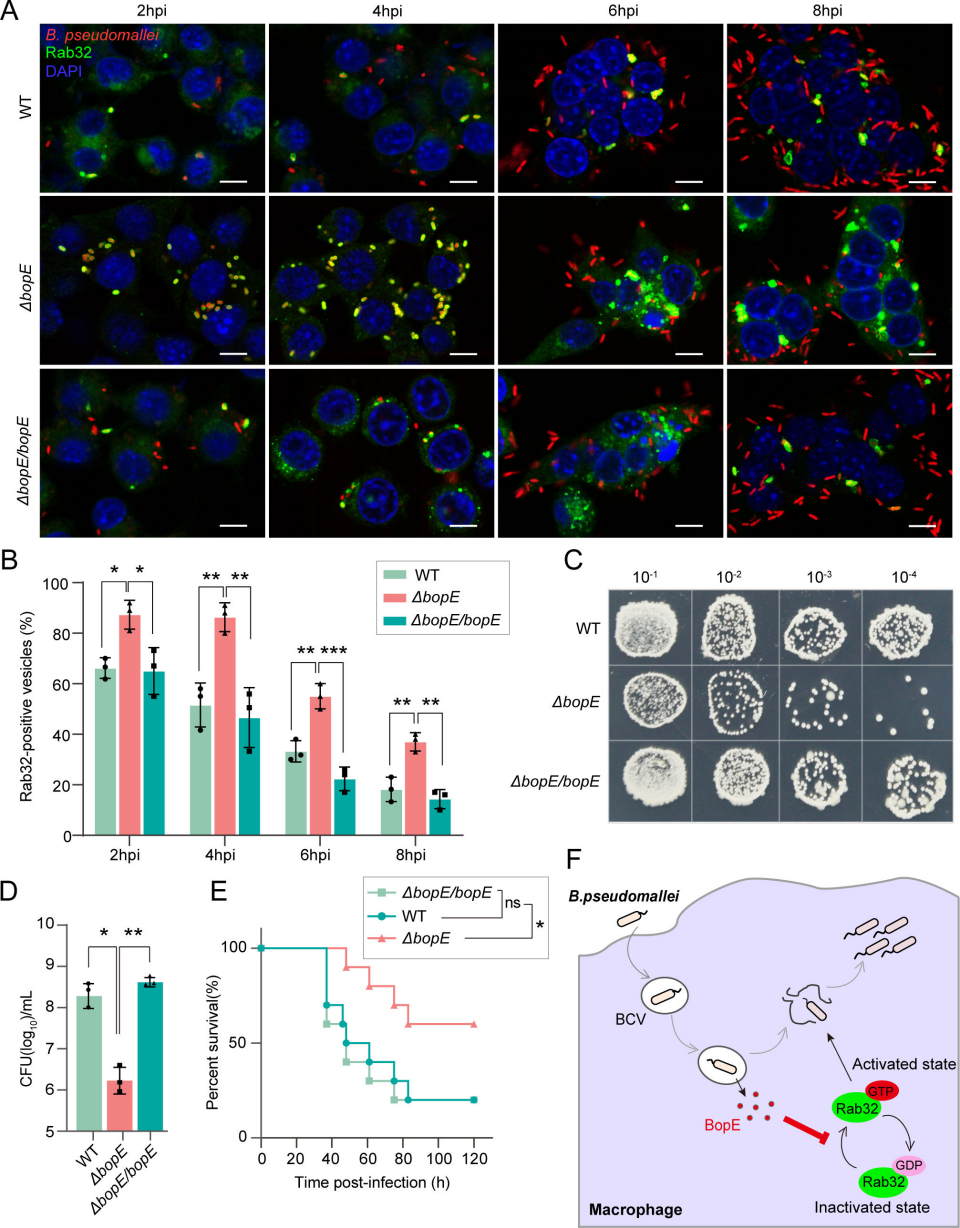
BopE targets the Rab32-dependent defense pathway allowing *B. pseudomallei* to survive in the host. (**A**) Representative immunofluorescence images of co-localization of Rab32 and *B. pseudomallei* mutant strains in RAW264.7 cells at the indicated times after infection. Scale bar, 50 µm. (**B**) Quantification of *B. pseudomallei* in Rab32-positive vesicles in panel **A**. Ten random fields were counted in each slice, and three different experiments were conducted in every group. All assessments were performed in a blinded manner. *P < 0.05, **P < 0.01, and ***P < 0.001 (one-way ANOVA). (**C and D**) Intracellular *B. pseudomallei* burdens in RAW264.7 cells at 8 hpi. WT is the wild-type strain of *B. pseudomallei*, Δ*bopE* is the *bopE* deficient strain, and Δ*bopE/bopE* is the *bopE* complementation strain. The data were showed as means ± SD. Three different experiments were conducted in each group. *P < 0.05 and **P < 0.01 (one-way ANOVA). (**E**) Survival rate of WT, Δ*bopE*, or Δ*bopE/bopE B. pseudomallei*-infected BALB/c mice. Mice models were infected with 4 × 10^5^ CFU of *B. pseudomallei* WT, Δ*bopE*, or Δ*bopE/ bopE* and then continued to be observed until day 5. χ^2^ = 4.101, *P* = 0.0429 (Δ*bopE* vs. WT); χ^2^ = 5.141, *P* = 0.0234 (Δ*bopE* vs. Δ*bopE/bopE*), 12 mice per group (log-rank test). (**F**) The graphical model illustrates that BopE, a T3SS-3 effector of *B. pseudomallei*, suppresses the Rab32-dependent defense pathway in macrophage, thereby facilitating intracellular replication.

To examine the potential effects of the BopE-Rab32 interaction on the Rab32 pathogen-restriction pathway. We developed BopE-deficient (Δ*bopE*) and complemented (Δ*bopE*/*bopE*) strains of *B. pseudomallei* (Fig. S5). Confocal microscopy analysis was performed at various time points post-infection. We observed that Rab32 was efficiently recruited to BCVs within 2 hpi, followed by a decline in recruitment at 4 hpi. Notably, the number of Rab32-positive BCVs was significantly higher in the Δ*bopE*-infected monolayers than in the wild-type and complement infected cells (Fig. 6A and B). Our findings suggest that BopE plays a role in suppressing the recruitment of Rab32 to BCVs.

Furthermore, we explored the effects of BopE on the intracellular survival and the virulence of *B. pseudomallei*. We observed a significant reduction in intracellular bacterial loads within the BopE-deficient strain infection group, in contrast to the groups infected with the wild-type or BopE complement strains, at 8 hpi as shown in Fig. 6C and D. We then sought to determine the role of BopE in the Rab32-dependent host pathogen-restriction pathway during *B. pseudomallei* infection *in vivo*. Consistent with our hypothesis, the BopE-deficient mutant strain of *B. pseudomallei* showed obviously attenuated virulence and reduced mortality in infected mice, compared with wild-type strain- and BopE complement strain-infected groups (Fig. 6E). Taken together, our results demonstrate that BopE interacts with Rab32 and likely suppresses its activation at the nucleotide exchange level, thereby preventing Rab32’s recruitment to BCVs, thus counteracting the host pathogen-restriction pathway and facilitating intracellular replication and virulence of *B. pseudomallei* in the host, as illustrated in Fig. 6F.

## DISCUSSION

Our immune system is engaged in a continuous battle against invading pathogens to avoid infection. As an important part of the innate immune system, macrophages can actively phagocytose pathogens, form bacteria-containing phagocytic vesicles, then initiate membrane traffic, and transport bacteria-filled vesicles to degradative intracellular compartments. Recently, numerous studies emphasize the importance of Rab32-dependent membrane trafficking in the elimination of intracellular bacteria in macrophages (9, 12, 24). We have also previously described that the Rab32-dependent pathway can restrict the intracellular replication of *B. pseudomallei* (14). However, it is believed that *B. pseudomallei* has the ability to escape from defensive BCVs and replicate in the cytoplasm (15). After invading host cells, *B. pseudomallei* often employ several strategies to escape the host cell immune system. It can circumvent autophagy either by modulating ATG10 or by manipulating the host’s lipid metabolism for its intracellular survival (36, 37). Additionally, *B. pseudomallei* can trigger the PERK-mediated unfolded protein response, a mechanism that supports its survival both *in vivo* and *in vitro*, thereby maintaining its survival and proliferation within a variety of host cells (38, 39). However, the mechanism by which *B. pseudomallei* escapes from Rab32-dependent vesicles remains to be elucidated.

In this study, the overexpression of Rab32 led to the accumulation of Rab32-positive vesicles and increased the probability of containing *B. pseudomallei*, thereby inhibiting *B. pseudomallei* intracellular survival. While interference with Rab32 expression showed the opposite effect. This is consistent with our previous findings that macrophages can specifically detect *B. pseudomallei*, triggering an upregulation of Rab32 as part of the host immune response (14). Interestingly, we found not all intracellular *B. pseudomallei* were encompassed by Rab32-positive vesicles despite Rab32 overexpression. This implies that *B. pseudomallei* may employ certain strategies to counteract the bactericidal effects of the Rab32-dependent pathway. In a parallel study, Rab32 is strongly recruited to human-adapted pathogen *Salmonella enterica* serovar Typhi (*S. Typhi*)-containing phagosome vesicle and restrict the replication of *S. Typhi*, but not the broad host range *Salmonella enterica* serovar Typhimurium (*S. Typhimurium*), as these *serovars* have evolved potent bacterial effectors, SopD2 and GtgE, to neutralize the Rab32-dependent defense pathway (24). Pathogens that enter the endocytic network must subvert membrane trafficking to avoid further transportation to degradative intracellular compartments. Many pathogenic bacteria accomplish this subversion with effector proteins delivered by specialized secretion systems, which can directly modulate membrane traffic (40, 41).

To identify potential effectors in *B. pseudomallei* that target the Rab32-mediated host defense pathway, specifically by preventing Rab32 recruitment to BCVs, we conducted a transcriptomic analysis of *B. pseudomallei*’s response to macrophage-mediated immune clearance. This analysis revealed that genes associated with T3SS of *B. pseudomallei* were significantly enriched among the DEGs. Our findings align with the results obtained through the innovative “TRANSITomic” approach, which has detailed the transcriptomes of individual *B. pseudomallei* cells at distinct stages of host cell infection, demonstrating that *B. pseudomallei* strategically deploys its T3SSBsa to escape the vesicle to gain entry into the host cell cytoplasm (16). We also observed a significant upregulation of DEGs in the type VI secretion system (T6SS) of *B. pseudomallei* at 4 hpi, including genes such as *hcp1*, *tssA*, and *tssB*, as detailed in Table S3. This is consistent with a previous report that highlighted the significant modulation of *B. pseudomallei* T6SS over 6 hours of infection (42).

T3SS is the key virulence for *B. pseudomallei* by injecting bacterial effector proteins into host cells. Once inside host cells, the effector proteins subvert host cell processes in favor of the bacteria (21, 43). Among the DEGs, we found that a variety of effector proteins of *B. pseudomallei*, such as BprD, BipC, and BopE, were highly expressed in the state of infection. Specifically, BopE displays significant homology to *Salmonella* effector proteins SopE and SopE2 (Fig. S6). It has been characterized as GEFs for the Rho GTPases Cdc42 (cell division cycle 42) and Rac1, which promote the invasion of *B. pseudomallei* into host cells (32). Moreover, the amino acids 78–261 of BopE have been reported as a GEF catalytic domain; the mutant BopE^N224P/R230Q^ showed high catalytic activity, but BopE^R207E/N216P^ lost its catalytic activity (34). Interestingly, in our study, we highlighted BopE interacting with Rab32 indeed has an inhibitory effect on the activation of Rab32 at the level of nucleotide exchange. Crucially, this inhibition is independent of the GEF activity of BopE, as demonstrated by the mutants BopE^N224P/R230Q^ and BopE^R207E/N216P^. Our results suggest that BopE may have distinct effects on various small GTPases in the host. It is similar to *Salmonella* effector SopE2, which acts as a GEF for Rho GTPase Cdc42 but not Rac1 (44). Likewise, *Salmonella* effector SopD is known as a GAP that deactivates Rab8, while in the late stages of infection, SopD activates Rab8 by promoting its dissociation from guanine nucleotide dissociation inhibitors (35). Besides, BopE also appears to localize to the host-cell membrane; this could be a part of the bacterial strategy to manipulate host cell processes to facilitate its infection and survival. But how BopE localizes to the host cell membrane, which proteins on the host cell membrane it interacts with, and how it affects the infection process are questions that require extensive *in vitro* and *in vivo* experimentation for further clarification. Collectively, we hypothesize that the dual functionality of BopE facilitates the invasion of *B. pseudomallei* into host cells while simultaneously suppressing the Rab32-dependent defense pathway, enhancing its intracellular survival. Thereby, a comprehensive understanding of the role of BopE in modulating host cell functions necessitates further research to elucidate.

In summary, the current study builds upon the foundational work of Hu et al., who established the critical role of Rab32 in the intracellular survival of *B. pseudomallei*. This study not only validates our previous observations but also provides a significant extension to this body of knowledge. We demonstrate that *B. pseudomallei* employs the T3SS-3 effector BopE to subvert the Rab32-dependent defense pathway, thereby enhancing its intracellular replication and virulence. This discovery elucidates a critical aspect of the evasion strategy of *B. pseudomallei* and underscores the importance of Rab32 in the pathogenesis of melioidosis. Moreover, our findings may offer valuable insights for the development of targeted therapeutic interventions.

## MATERIALS AND METHODS

### Bacterial strains, plasmids, and culture conditions

*B. pseudomallei* (BPC006, NC_018529.1, and NC_018527.1) and *Escherichia coli* were cultured on Luria broth (LB) agar plates or in liquid LB media at 37°C with shaking at 200 rpm. *E. coli* DH5α strain was used for plasmid construction and cloning. *E.coli* S17-1/λpir strain was used as plasmid donor strain for conjugation experiments. Trimethoprim (Tp; 200 µg/mL) or kanamycin (Kan; 250 µg/mL) was utilized for the screening of *B. pseudomallei*. Live *B. pseudomallei* was carried out under standard laboratory conditions (biosafety level 3 [BSL-3]). Bacterial strains and plasmids are listed in Table S1.

### Cell culture and infection

HEK293T and RAW264.7 were cultured in Dulbecco’s modified Eagle medium (DMEM; Thermo Fisher C11995500BT) supplemented with 10% fetal bovine serum (FBS; Gibco 10091148) at 37°C in a humidified atmosphere with 5% CO_2_. Cell infection with *B. pseudomallei* was carried out as previously described (14). Briefly, cells were seeded overnight and infected with the *B. pseudomallei* at a multiplicity of infection (MOI) of 10 for 1.5 hours. Then, cells were washed twice with phosphate-buffered saline (PBS) and incubated in fresh medium containing 250 µg/mL kanamycin to kill the extracellular bacteria. At the selected time points, macrophage cells were lysed using RIPA for western blot or TRIzol for RT-qPCR or fixed in 4% paraformaldehyde for immunofluorescence or lysed using 0.1% Triton X-100 for plate counting to determine the intracellular bacterial counts. The number of bacteria was estimated by measuring the absorbance of the bacterial suspension at 600 nm.

### Gene silence and overexpression

*Rab32* was knocked down by a small interfering (si)RNA that targets the *Rab32* gene (NM_026405) in RAW264.7 cells and overexpressed by the plasmid of pcDNA4-Rab32. Briefly, RAW264.7 cells were seeded till they reached 70%–80% confluence; then, siRNA oligo nucleotides targeting mouse *Rab32* (siRab32; Santa Cruz, sc-152636) or pcDNA4-Rab32 (pRab32) were directly mixed with Advanced DNA RNA Transfection Reagent (ZETA LIFE, USA) according to the manufacturer’s instructions. Following incubation at room temperature for 10 min, the complex was then added to cell culture plates. After 24 hours of incubation, cells were harvested and the expression levels of Rab32 were detected by western blotting. Cells that received no treatment were used as NC. All experiments were performed in triplicate.

### Bacterial survival assays

Intracellular survival of *B. pseudomallei* in RAW264.7 cells was estimated as previously described with some modifications (45). Briefly, cells were infected with either WT or *bopE* mutant of *B. pseudomallei* as described above. At the indicated time points, the infected cells were washed with PBS three times and lysed with 0.1% Triton X-100 (Sigma, T8787). Cell lysates were serially diluted 10-fold with PBS and plated onto LB agar. CFU were then measured after 36 hours of incubation at 37°C. We performed the experiment three replicates independently.

### Western blot

The expression of target proteins was detected by western blot as previously described with some modifications (39). Briefly, equal amounts of denatured proteins were separated by SDS-PAGE and then transferred to a polyvinylidene difluoride membrane (Millipore, IPVH00010). The membranes were blocked with 5% (wt/vol) skimmed milk for 2 hours, followed by an overnight incubation at 4°C with Rab32 (Proteintech 10999-1-AP; 1:1,000), Flag tag (ZENBIO R24091; 1:1,000), GFP tag (Bioss bsm-33019M; 1:1,000) antibody, or anti-BopE rabbit serum (1:500). Membranes were subsequently incubated with HRP-linked anti-rabbit IgG (CST 7074S; 1:5,000) or anti-mouse IgG (CST 91,196S; 1:5,000) secondary antibody at the room temperature for 2 hours. Blots were scanned with the ChemiDoc Touch System (Bio-Rad, USA) and analyzed using Image Lab Software (version 6.0.0, build 25, Bio-Rad, USA). GAPDH (CST 8884; 1:1,000) or DnaK (CUSABIO CSB-PA633459HA01EGW; 1:2,000) was used as loading control, and three independent replicates were performed.

### Immunofluorescence

Cells were seeded on coverslips (NEST, 801011) in 24-well plates and transfected with the indicated plasmids or siRNA. After incubation for 24 hours, cells were washed twice with PBS and fixed in 4% paraformaldehyde (Beyotime P0099) for 10 min; subsequently, cells were permeabilized with 0.3% Triton X-100 (amresco, 0694-1L) and incubated with Flag tag antibody (ZENBIO R24091; 1:500), Rab32 antibody (Proteintech 10999-1-AP; 1:300), or a mouse polyclonal antibody of *B. pseudomallei* (1:200) and subsequently with the Alexa Fluor 647 anti-mouse secondary antibody (Thermo Fisher A-31571; 1:2,000) or Alexa Fluor 488 anti-rabbit secondary antibody (Thermo Fisher A-11008; 1:2,000). Nucleus was stained with DAPI (Thermo Fisher 62248; 300 nM). The coverslips were mounted onto glass slides using anti-fade mounting medium (Invitrogen, S36967). Confocal images were taken with the Leica SP8 confocal microscope (Leica Microsystems) using a 60× lens objective.

### Intracellular bacteria isolation and RNA-seq

The BPC006 strain was cultured on the LB agar plate 36 hours at 37°C. A single colony was selected to inoculate into 5 mL of LB broth and incubated at 37°C for 12 hours. The cultures were divided into two equal parts (*in vitro B.ps* and intracellular *B. ps*) based on the bacterial count with an MOI of 100. Intracellular *B. ps* was used on infected RAW264.7 cells, and intracellular bacteria were isolated as previously described (46). Briefly, after 4 hours of infection, cells were washed extensively with PBS and lysed in 20 mM Tris-HCl (pH 7.6) buffer containing 150 mM NaCl and 0.5% Triton X-100. To recover intracellular bacteria, collected cell lysates were centrifuged at 600 × *g* for 5 min to remove nuclei and cell debris, and then, the supernatant was centrifuged at 4,000 × *g* for 20 min. The pellets were immediately washed with RIPA buffer (Beyotime P0013D) to remove residual host RNA and centrifuged at 6,000 × *g* for 5 min to get the final bacterial pellets. *In vitro B.ps* were cultivated in DMEM medium, without the presence of RAW264.7 cells, and were recovered as the group of intracellular *B. ps*.

The total RNA was extracted from the harvested bacteria using a RNAprep Pure Cell/Bacteria Kit (Tiangen DP340) following the manufacturers’ protocols and sent to the Sangon Biotech (Shanghai, China) company for quality control and complementary DNA (cDNA) library construction, followed by sequencing. Trimmomatic (version 0.36) was used to trim low-quality reads and remove adapters of raw data. The Bowtie2 (version 2.3.2) was used to align the trimmed sequence to *B. pseudomallei* C006 and matched to the *Burkholderia pseudomallei* K96243 genome (NC_006350.1and NC_006351.1). The DEGs in which |log_2_FoldChange| ≥ 1 and *P* value < 0.05 were analyzed using DESeq2 software (version 1.12.4). KOBAS 3.0 (http://bioinfo.org/kobas/) was used to determine the main biological functions of DEGs enriched in Kyoto Encyclopedia of Genes and Genomes pathway enrichment analyses (47).

### Real-time qPCR

To validate the data generated from GO analysis and pathway analysis, eight DEGs of effectors were selected from T3SS-1 for RT-qPCR analysis. The respective primers were listed in Table S2. The total RNA used for RT-PCR was extracted by TRIzol (Invitrogen Life Technologies, USA) according to the manufacturer’s recommendation. Next, cDNA was retro-transcribed from 1 µg of total RNA using a PrimeScript RT Reagent Kit with gDNA Eraser (Takara, Dalian, China). The cDNA samples were then amplified using SYBR Premix Ex Taq II (Takara, Dalian, China) on a CFX96 Touch Real-Time PCR Detection System (Bio-Rad, USA). Six replicates were respectively conducted in the infected group (I) and non-infected group (NI). Relative fold expression for target genes was calculated by the 2^−ΔΔCt^ method relative to *rpoB* as reference gene.

### Co-immunoprecipitation

The interactions between T3SS effectors and Rab32 were assessed using immunoprecipitation assays. Briefly, HEK293T cells were co-transfected with a combination of plasmids, including pEGFP-Rab32 (encoding Rab32 with a GFP tag) and pcDNA4-Flag-effectors (encoding T3SS effectors BapA, BipC, BopA, BopC, BopE, BprD, BapC, or BipD, each fused with a Flag tag). Additionally, pcDNA4-Flag-SopD2 was included as a positive control for the interaction (24). After 24 hours of transfection, cells were collected and lysed in lysis buffer (50 mM Tris-HCl, pH 7.4, 150 mM NaCl, 1% Triton X-100, and protease inhibitor cocktails) on ice. The supernatants of the cell lysates were isolated by centrifugation at 12,000 × *g* for 30 min at 4°C, and the supernatants were then incubated with anti-Flag M2 Affinity Gel (Sigma-Aldrich, A2220) overnight at 4°C. The resins were washed three times with lysis buffer followed by elution with 3× Flag peptide for 2 hours at 4°C. The IP samples were detected by western blot.

### Yeast two-hybrid assay

Yeast two-hybrid analyses were conducted using the Matchmaker Gold Yeast Two-Hybrid System. Strain Y2HGold was co-transformed with the bait Gal4-BD vector pGBKT7 where *B. pseudomallei bprD*, *bopE*, and *bipC* genes were inserted in frame (pGBKT7-BprD, pGBKT7-BopE, and pGBKT7-BipC) and the prey AD vector pGADT7 where the gene of *Rab32* was also inserted in frame (pGADT7-Rab32). Expression of BD and AD fusion proteins was confirmed by western blotting using anti-c-Myc antibody and anti-HA antibody, respectively. The transformants were plated onto low-stringency (lacking leucine and tryptophan) and high-stringency (lacking adenine, histidine, leucine, and tryptophan) selection plates for detection. The interaction between receptor-interacting protein kinase 1 (RIPK1) and the Fas-associated death domain (FADD) was well-documented (48); we used pGBKT7-mRIPK1 and pGADT7-mFADD co-transformed Y2HGold strain as a positive control in this study.

### Construction of *B. pseudomallei bopE* mutant and complementation strain

The construction of *B. pseudomallei bopE* mutant (Δ*bopE*) and complementation strain (Δ*bopE*/*bopE*) was performed as previously described with some modifications (25). The *BPSS1525* (*bopE*) mutant was constructed in a *B. pseudomallei* C006 background by double-crossover allelic exchange using the λpir-dependent pK18mobsacB vector. The clean deletion construct was confirmed by Sanger sequencing. For complementation, the full-length of the *bopE* gene was amplified from the genomic DNA of *B. pseudomallei* C006. After double enzyme digestion with *Xba* I and *Hin*d III, the product was cloned into the broad-host-range vector pUCP28T by T4 DNA Ligase (Tiangen Biotech Co. Ltd., Beijing, China) to generate pUCP28T-BopE, which expressed BopE under the control of the 16S promoter of *B. pseudomallei*. Then, the recombinant plasmid pUCP28T-BopE was electroporated into *B. pseudomallei* C006. Expression of BopE was confirmed by western blot. The plasmids and primer sequences used in this study were listed in Tables S1 and S2.

### Nucleotide exchange assay

BopE and the mutant BopE^N224P/R230Q^ or BopER^207E/N216P^ were expressed in *Escherichia coli* BL21(DE3) with the pET28a vector under isopropyl-β-d-thiogalactoside induction. Subsequently, the fusion proteins were purified through a HisTrap FF (5 mL) column (Cytiva, 17-5255-01) on an Akta pure 25 system. Nucleotide exchange assays were conducted using fluorescent N-methylanthraniloyl-GDP (Mant-GDP, Molecular Probes), following a modified method from a previous report (33). Briefly, Rab32 (Origene, TP502577) or Rac1 (Origene, TP527648) equilibrated in loading buffer (20 mmol/L HEPES-NaOH [pH 7.5], 50 mmol/L NaCl, 0.5 mmol/L MgCl_2_, 5 mmol/L EDTA, 1 mmol/L DTT) was loaded with 20-fold excess of Mant-GDP (Jena Bioscience, NU-204S) at room temperature for 1.5 hours. The loading reaction was terminated with 10 mM MgCl_2_, diluted with desalting buffer (20 mM HEPES [pH 7.5], 50 mM NaCl, 5 mM MgCl_2_, and 1 mM DTT), and desalted using a NAP-5 column (Cytiva, 17085301). Exchange reactions (200 μL total volume) were performed in 20 mM HEPES (pH 7.5), 50 mM NaCl, 5 mM MgCl_2_, and 1 mM DTT and contained 30 µM Rab32 in the presence or absence of BopE or BSA. After a 10-min pre-incubation on ice, reactions were initiated with 200 µM GTP (Macklin, G810427) and data were acquired at 25°C using a Molecular Devices SpectraMaxiD5 microplate spectrofluorometer with excitation at 360 nm and emission at 440 nm.

### Infection-induced mouse melioidosis model

Specified pathogen-free BALB/c mice at approximately 6–8 weeks were purchased from Vital River Laboratory Animal Technology Co. Ltd. (Beijing, China) and were maintained under barrier conditions in a BSL-3 biohazard animal room and provided with free water and diet and a 12-hour light/dark cycle. After 1 week of adaptive feeding, mice were intraperitoneally injected with 4 × 10^5^ CFU of *B. pseudomallei* (WT, Δ*bopE*, and Δ*bopE*/*bopE*, 12 mice per group) and continuously observed for 5 days.

### Statistical analysis

The quantified data with statistical analysis were performed using GraphPad Prism (v9.5.1) software. Unpaired two-sided Student’s *t*-test, one-way analysis of variance (ANOVA), or two-way ANOVA followed by multiple comparisons was used for statistical analyses. Significance was indicated in the figures and figure legends. All experimental data from the assays were obtained from at least three separate biological samples, and the results were presented as the average values with their standard deviations (means ± SD). The statistical tests and the numbers of samples were indicated in the corresponding figure legends.

## SUPPLEMENTAL MATERIAL

Supplemental material msphere.00453-24-s0001.pdf Movie legend, Figures S1 to S6, and Tables S1 to S3.

Movie S1 msphere.00453-24-s0002.avi Recruitment of Rab32 vesicles to capture intracellular *B. pseudomallei*.

ASM does not own the copyrights to Supplemental Material that may be linked to, or accessed through, an article. The authors have granted ASM a non-exclusive, world-wide license to publish the Supplemental Material files. Please contact the corresponding author directly for reuse.
