# Supplementary material for: Burkholderia pseudomallei BopE suppresses the Rab32-dependent defense pathway to promote its intracellular replication and virulence
Source: mSphere. 2024 Oct 21;9(11):e00453-24. doi: 10.1128/msphere.00453-24 (PMC11580396; doi:10.1128/msphere.00453-24)
Supplement: Supplemental material — Movie legend, Figures S1 to S6, and Tables S1 to S3. [file msphere.00453-24-s0001.pdf]

Supplemental Information

Burkholderia pseudomallei BopE suppresses Rab32-dependent defense pathway to promote its intracellular replication and virulence

Chenglong Rao[a, †], Ziyuan Zhang[a, †], Jianpeng Qiao[a,b], Dongqi Nan[a], Pan Wu[a], Liting Wang[c], Changhao Yao[a], Senquan Zheng[a], Jinzhu Huang[a], Yaling Liao[a], Wenzheng Liu[a], Zhiqiang Hu[a], Shiwei Wang[a], Yuan Wen[a], Jingmin Yan[a], Xuhu Mao[a, ✉] and Qian Li[a, ✉].

[†] These authors contributed equally to this work.

✉ Corresponding Author:

Qian Li

Email: liqianjane@tmmu.edu.cn

Xuhu Mao

Email: maoxuhu2023@tmmu.edu.cn

This file includes:

Supplemental movie

Movie S1

Supplemental Figures

Figure S1 to S6

Supplemental Tables

Table S1 to S3

Supplemental movie

Movie S1. The Recruitment of Rab32 Vesicles to capture the intracellular *B. pseudomallei*. pEGFP-Rab32 transfected RAW264.7 cells were infected with *B. pseudomallei* (tagRFP) (MOI = 10). Red fluorescent strain of *B. pseudomallei* was genetically engineered to allow the red fluorescent protein expression under the control of the 16S promoter, which marks *B. pseudomallei* with red fluorescence. The dynamic process of containment was recorded by live cell video microscopy. Rab32 is recruited to *B. pseudomallei*-containing vesicles, but not all intracellular *B. pseudomallei* were in Rab32-positive vesicles, supporting Figure 1.

Supplemental Figures

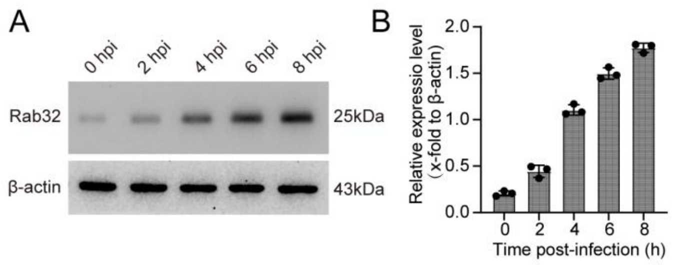

Figure S1 Temporal dynamics of Rab32 expression in RAW264.7 following *B. pseudomallei* infection. (A) Western blot analysis of Rab32 in RAW264.7 cells during *B. pseudomallei* infection. RAW264.7 cells were infected with *B. pseudomallei* at an MOI of 10, and the cell lysis at 2hpi,4hpi,6hpi and 8hpi were detected. (B) The relative expression levels of Rab32 are derived by quantifying the band intensities presented in (A). The ratio of the intensities of all Rab32 bands to that of β-actin have been calculated. All data are presented as mean ± SD from three independent experiments.

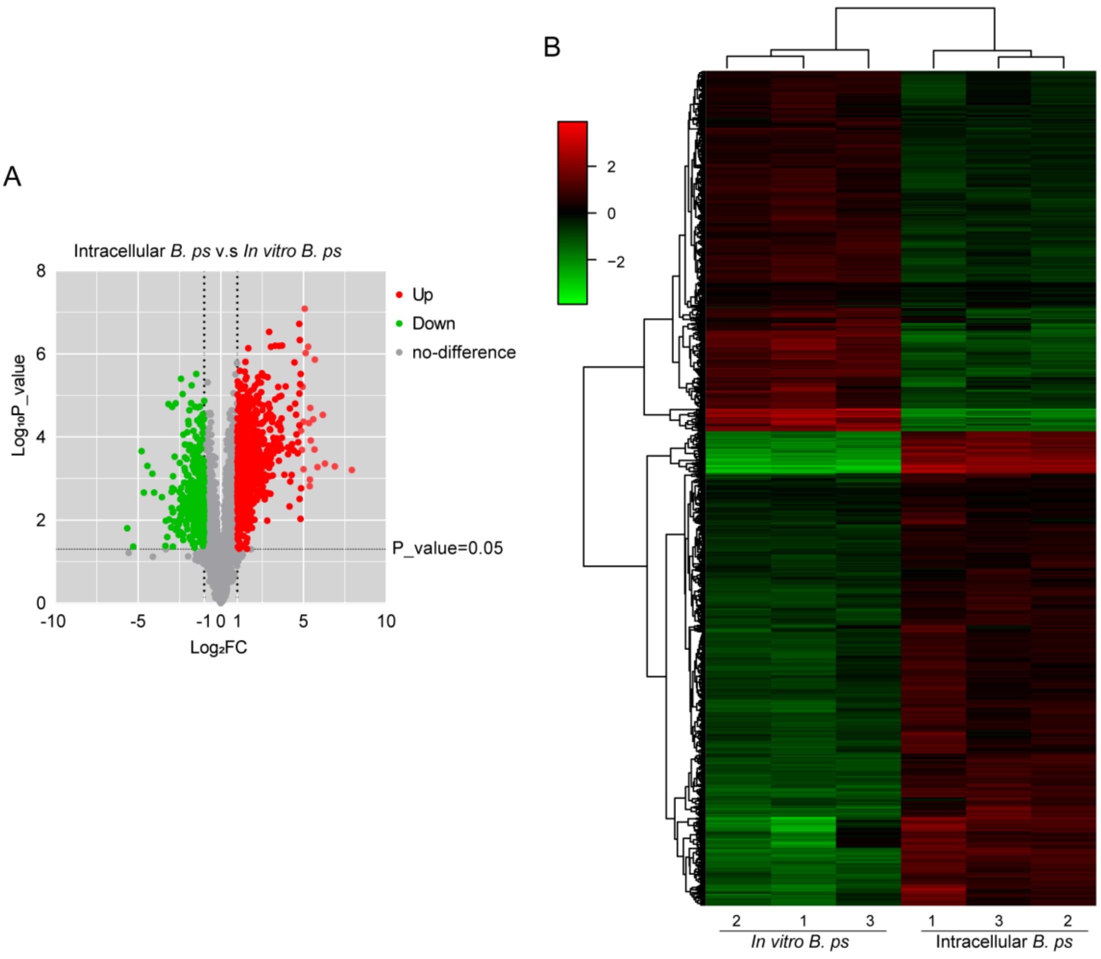

Figure S2. **Comparative transcriptomic analyses of *B. pseudomallei* between culturing *in vitro* and intracellular infection groups.** (A) Volcano plots representing genes expressed in *B. pseudomallei* cultured *in vitro* (*In vitro B. ps*) and intracellular infections (Intracellular *B. ps*). Red and green dots present the DEGs of intracellular *B. ps*. Counts of genes up-regulated or down-regulated are shown in red or green dots, respectively, and a cut-off level of 2-fold change and a P-value < 0.05 were applied. (B) The heatmap of all the expression genes in both *in vitro B. ps* and intracellular *B. ps* groups. Expression from low to high is indicated by a gradient from green to red, three repetitions per group.

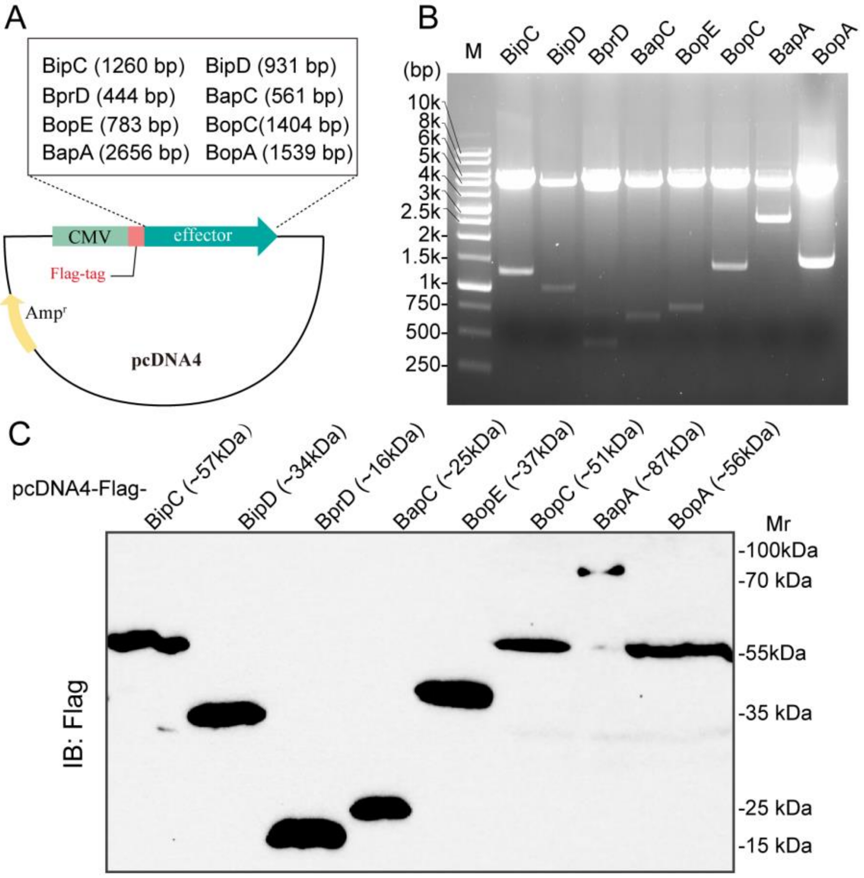

Figure S3. **Constructed the plasmids for T3SS effector proteins tagged with Flag.** (A) Schematic representation of the strategy to obtain the plasmids for T3SS effector proteins tagged with Flag. (B) Fusion expression plasmids were detected by appropriate enzyme digestion. Specifically, the pcDNA4-BapC construct was identified with B*am*H I and X*ba* I, which introduced a 106 base pair tag gene sequence upstream of BapC,

yielding a 668 base pair band after digestion. Apart from this, all other constructs were digested using their respective restriction enzymes listed in Supplementary Table 2, yielding sizes consistent with the effector protein genes. Furthermore, all pcDNA4 constructs were confirmed to be correct by sequencing. (C) Fusion expression plasmids were transfected into HEK293T cells, respectively, and detected by Flag antibody, the molecular mass of the effector proteins is denoted in corresponding parentheses.

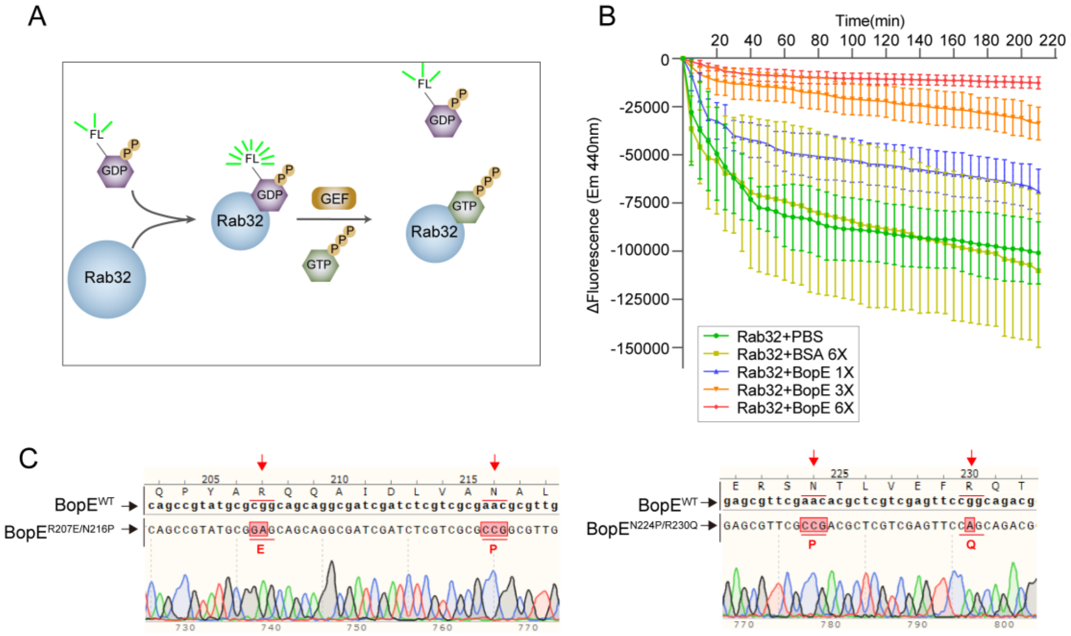

Figure S4. BopE inhibits the activation of Rab32 by interference with the nucleotide exchange process. (A) The mode diagram for the nucleotide exchange assay utilizing Mant-GDP. Mant-GDP exhibits optimal fluorescence when bound to protein, and the exchange of GTP for GDP on the protein leads to a significant reduction in fluorescence. (B) BopE suppressed Rab32 activation in a dose-dependent manner. Rab32 was loaded with Mant-GDP. Following desalting, exchange reactions were performed supplemented with BopE or BSA at the indicated ratios. Reactions were monitored by spectrofluorometry (excitation at 360 nm, emission at 440 nm). (C) The sequencing results of the BopE GEF activity mutants. The mutant BopE$^{N224P/R230Q}$ exhibits high catalytic activity, while the mutant BopE$^{R207E/N216P}$ has lost its catalytic activity. The arrows indicate the mutation sites.

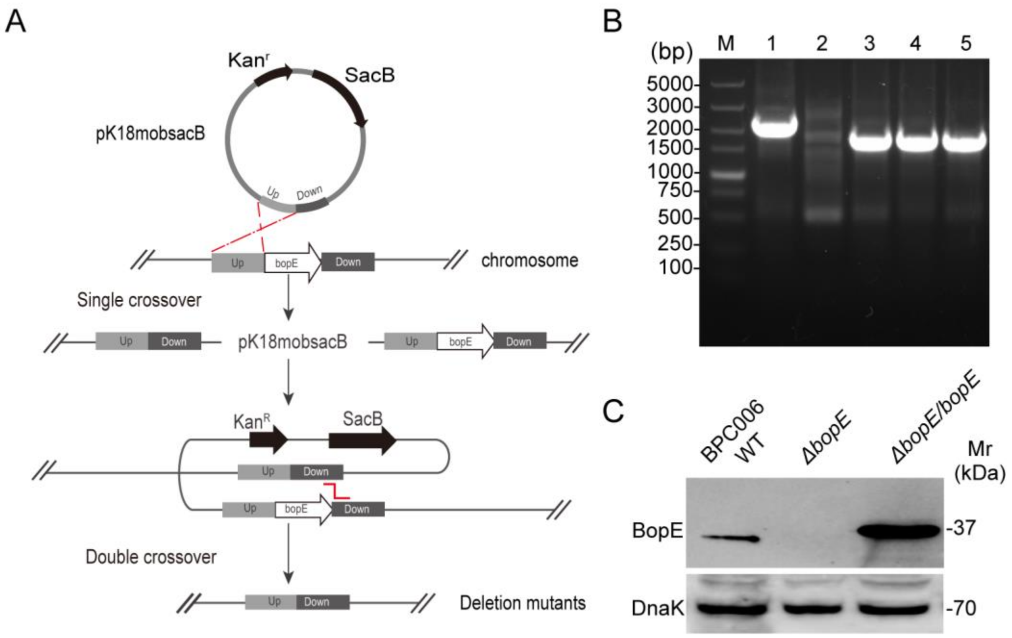

Figure S5. Generating *bopE* deficient strain (*ΔbopE*) and complementation strains (*ΔbopE/bopE*) of *B. pseudomallei.* (A) Schematic representation of the strategy to obtain markerless *ΔbopE* strains using the counterselective properties of the sacB gene. Kanamycin is referred to Kan. (B) Validation of *bopE* knockout strains by PCR. The M lane represents the DNA marker and lane 2 is *E·coli* as a negative control, lanes 3-5 are the detected *bopE* knockout strains by PCR using primers BopE-up-F and BopE-down-R listed in Supplementary table 2. (C) Detected the expression of BopE in *B. pseudomallei* WT, *ΔbopE* or *ΔbopE/bopE* strain by rabbit anti serum against BopE, respectively.

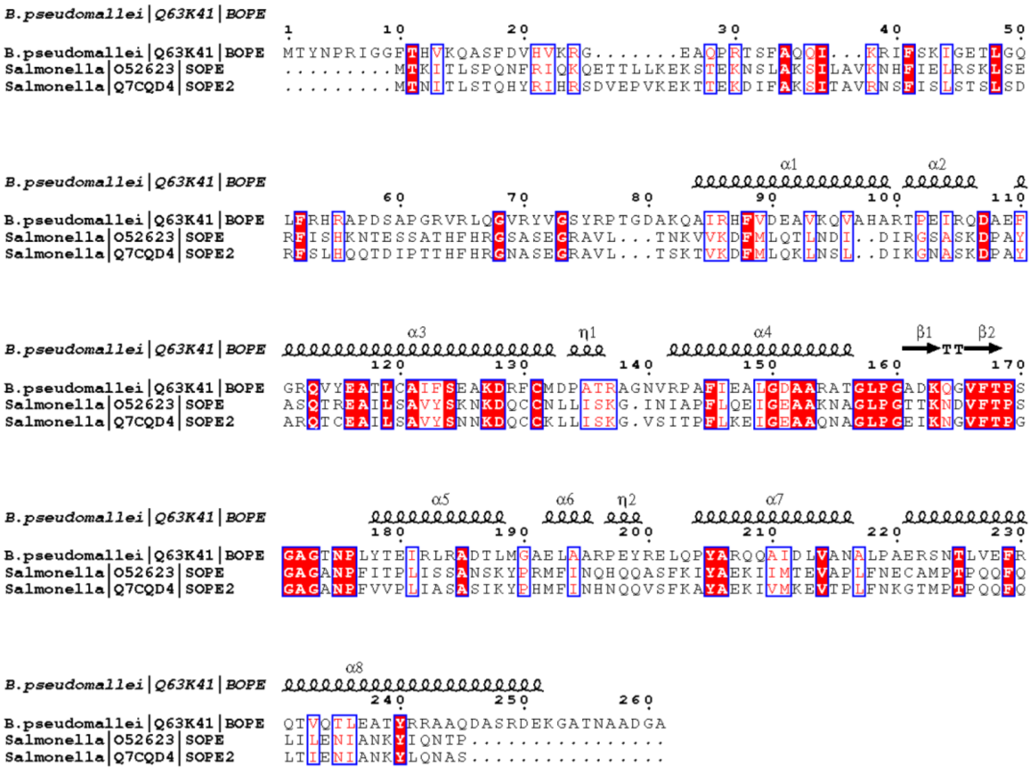

Figure S6. Multiple sequence alignment of BopE and SopE/SopE2. The sequence of *B. pseudomallei* BopE (Q63K41) and *Salmonella enterica* SopE (O52623) and SopE2 (Q7CQD4) was download on Uniport (https://www.uniprot.org/uniprotkb/). The structure *B. pseudomallei* BopE (PDB ID: 2JOK) was download on Protein Data Bank (www.rcsb.org/). Multiple sequence alignment was done using ClustalW, and the alignment of the secondary structures were done using ESPript 3.0.

Supplemental Tables

Supplementary Table 1. The list of bacterial strains and plasmids used in this study

Strain or plasmid	Relevant characteristic	Source or reference
B. pseudomallei		
BPC006	B. pseudomallei wild-type strain	Fang Y et al., 2012 [1]
ΔbopE	BPC006 *ΔbopE*, codon 1-261 of *BPSS1525* (*bopE*) were deleted	This study
ΔbopE/bopE	bopE-complemented strain by pUCP28T-BopE	This study
BPC006-tagRFP	BPC006 was tagged with RFP by pUCP28T-RFP	This study
E.coli		
DH5α	Cloning host	This study
S17-λpir	Donor strain for conjugation	Jocelyn Wong et al.,2015 [2]
**Yeast**		
Y2HGold	GAL4 yeast two-hybrid experimental strain, containing distinct ADE2, HIS3, MEL1, and AUR1-C reporter constructs that are only expressed in the presence of GAL4-based protein interactions.	Clontech (Cat. No. 630498)
**Plasmid**		
pK18mobsacB	Conjugative, suicide vector	Jocelyn Wong et al.,2015 [2]
pUCP28T	Broad-host-range vector; oriT and mob region from RP4, Tp^r	K. L. Chua et al., 2003 [3]
pUCP28T-BopE	Contains a 444bp full-length of *B. pseudomallei bopE* gene and P16s promoter inserted into the front of *bopE*	This study
pUCP28T-RFP	Contains a 678bp full-length of *mRFP* gene and P16s promoter inserted into the front of *mRFP*	This study
pEGFP-Rab32	Mouse Rab32 protein expressed with EGFP-tag in pEGFP-C1 were synthesized from Sangon Biotech (Shanghai, China).	This study
pGBKT7	pGBKT7 was used to express the fusion protein of the GAL4 DNA-binding domain (BD; amino acids 1–147) and the target	Clontech (Cat. No. 630443)

	protein (Bait). Kan^R, The screening marker was Trp.	
pGADT7	pGADT7 was used to express the fusion protein of GAL4 activation domain (AD; amino acids 768–881) and the target protein (Prey). Amp^R, The screening marker was Leu.	Clontech (Cat. No. 630442)
pGBKT7-mRIPK1	pGBKT7-mRIPK1 was a positive control plasmid encoding the fusion protein of mouse receptor-interacting serine/threonine-protein kinase 1 and GAL4-BD.	This study
pGADT7-mFADD	pGBKT7-mFADD was a positive control plasmid encoding the fusion protein of mouse FAS-associated death domain protein and GAL4-AD.	This study
pcDNA4-Flag	Mammalian cell expression vector with HA and Flag tags in the N-terminus, CMV promoter, Amp^R.	This study
pcDNA4-$SopD_2$	The N-terminus of *Salmonella typhimurium* $SopD_2$ was tagged with Flag in pcDNA4-Flag.	This study
pcDNA4-BipC	B. pseudomallei BipC expressed with Flag in pcDNA4-Flag.	This study
pcDNA4-BipD	B. pseudomallei BipD expressed with Flag in pcDNA4-Flag.	This study
pcDNA4-BprD	B. pseudomallei BprD expressed with Flag in pcDNA4-Flag.	This study
pcDNA4-BapC	B. pseudomallei BapC expressed with Flag in pcDNA4-Flag.	This study
pcDNA4-BopE	B. pseudomallei BopE expressed with Flag in pcDNA4-Flag.	This study
pcDNA4-BopC	B. pseudomallei BopC expressed with Flag in pcDNA4-Flag.	This study
pcDNA4-BapA	B. pseudomallei BapA expressed with Flag in pcDNA4-Flag.	This study
pcDNA4-BopA	B. pseudomallei BopA expressed with Flag in pcDNA4-Flag.	This study
pGADT7-Rab32	pGADT7-Rab32 was used to express the fusion protein of	This study

	GAL4 activation domain (AD) and the prey of mouse Rab32 protein.	
pGBKT7-BopE	pGBKT7-BopE was used to express the fusion protein of B. pseudomallei BopE and GAL4-BD.	This study
pGBKT7-BprD	pGBKT7-BprD was used to express the fusion protein of B. pseudomallei BprD and GAL4-BD.	This study
pGBKT7-BipC	pGBKT7-BopE was used to express the fusion protein of B. pseudomallei BipC and GAL4-BD.	This study

References

[1] FANG Y, HUANG Y, LI Q, et al. First genome sequence of a *Burkholderia pseudomallei* Isolate in China, strain BPC006, obtained from a melioidosis patient in Hainan [J]. Journal of bacteriology, 2012, 194(23): 6604-5.

[2] WONG J, CHEN Y, GAN Y H. Host Cytosolic Glutathione Sensing by a Membrane Histidine Kinase Activates the Type VI Secretion System in an Intracellular Bacterium [J]. Cell host & microbe, 2015, 18(1): 38-48.

[3] CHUA K L, CHAN Y Y, GAN Y H. Flagella are virulence determinants of *Burkholderia pseudomallei* [J]. Infection and immunity, 2003, 71(4): 1622-9.

Supplementary Table 2. The list of primers used in this study

Primers name	Primers sequence (5’→3’) [a]	Restriction enzymes	Amplicon size(bp)
**The primers for qPCR**			
BapA-F	CGCACCGATCAGTTGCTCAA		166
BapA-R	CGTTGTTCTCGTTATGATTGTTGAC		
BipC-F	AGATGAAGGGCGATCTGCTCCA		115
BipC-R	GCGTTCTCGTCCTCTTTCTGCT		
BopA-F	GTTGGACAAGGATGCCGAATGG		135
BopA-R	AACATCGCCGCCTCGTTCAG		
BopC-F	AGATGGCTGGCGTGAGGCTA		103
BopC-R	GCGGCTGTCAGTACCTTGTCTT		
BopE-F	CGTCGTTCGCACAACAGATCAA		197
BopE-R	GACCTGCTTGACCGCTTCGT		
BprD-F	GTGCTCAAGGAGATCCGCTTCA		142
BprD-R	TGACCGCCTCGAACATCTTGC		
BapC-F	ATTGATCTGCATCAGGCCGATA		108
BapC-R	AATGCCGATCTGCTGCGTTC		
BipD-F	CGCTCAGCACCTTGACCAGATT		101
BipD-R	CTCCGGCCAGAAGGACAACATC		
BsaN-F	CGGCGAAATTGCTCGCTTTT		139
BsaN-R	ATCAGCCACCGGTCGAATTT		
BicA-F	CGACGTGAACATAGACGACATCG		125
BicA-R	CATAGATGCCGTCCATCAGGT		
RpoB-F	GTTCCATCGTTCACCAAGTG		68
RpoB-R	TTGCAGAAATGTGCTGAATG		
**The primers for the purification of BopE**			
BopE-F	GCGGATCCGAATTCACGGGCGACGCGAAACAGGCGATCC	*Bam*H I	577
BopE-R	GCAAGCTTTCACGCGCCGTCCGCCGCGTTC	*Hind* III	
**The primers for gene knockout and complement**			
BopE-Up-F	CTATGACATGATTACGAATTTGCTCGGCGATCGACACC		976
BopE-Up-R	CGTTACTGCGGTCTTGCTCTCGGTTGAAGGG		
BopE-Down-F	AGAGCAAGACCGCAGTAACGTGGTCCGGAC		977
BopE-Down-R	ACGACGGCCAGTGCCAAGCTCGTCGCGCACGCGGACTG		
28TBopE-F	GCTCTAGAATGACTTACAACCCGAGAATCGGC	*Xba* I	989
28TBopE-Flag-R	CCAAGCTTTCACTTGTCATCGTCGTCCTTGTAATCCGCGCC GTCCGCCGCGTT	*Hind* III	
**The primers for effector protein expression by pcDNA4**			
BapA-F	GGGAATTCATGCCGCCGTCCATTCACCG	*Eco*R I	2656
BapA-R	TCTCTAGAGGTCATCGCTTCGTGCCGTTGG	*Xba* I	
BapC-F	GAGGCCGGCCGATGAGCGCCCGGAGGGCC	*Fse* I	561
BapC-R	CCTCTAGACTCACTGCACGCTAGCCTCCTC	*Xba* I	
BipD-F	GAGGCCGGCCGATGAATATGCATGTTGATATGG	*Fse* I	931

BipD-R	TCTCTAGAGGTTAAATCTGCAGATAGCTTTTGGC	*Xba* I	
BipC-F	GGGAATTCATGTCAATCGGTGTGCAAAGCAG	*Eco*R I	1260
BipC-R	TCTCTAGAGGTCAGGTCCGCAGATTGCCGG	*Xba* I	
BopA-F	GGGAATTCATGATCAATGTCGATGCATTCGTCG	*Eco*R I	1539
BopA-R	TCTCTAGAGGTCAGGCGGCGATCGCAGC	*Xba* I	
BprD-F	GGGAATTCATGAAGCTCAATGGAATGACCGC	*Eco*R I	444
BprD-R	TCTCTAGAGGTCACGGCGCCGGGCGCTG	*Xba* I	
BopC-F	TTGGTACCATGAGTACCGACGATCTCGTCAAAG	*Kpn* I	1404
BopC-R	TAGGATCCTGCGAGTGGGGTGTCCTG	*Bam*H I	
BopE-F	TTGGTACCATGACTTACAACCCGAGAATCGGC	*Kpn* I	783
BopE-R	TAGGATCCCGCGCCGTCCGCCGCGTT	*Bam*H I	
Rab32-F	CCGAATTCATGGCGGGCGAGGGACTAG	*Eco*R I	697
Rab32-R	GCAAGCTTTTAGCAGCAGCACTGGGACCTG	*Hin*d III	
**The primers for effector protein expression by pEGFP-C1**			
BopE-F	TCAAGCTTATGACTTACAACCCGAGAATCGGC	*Hin*d III	802
BopE-R	GTGGATCCTTACGCGCCGTCCGCCGCGTTC	*Bam*H I	
BopE$^{N224P/R230Q}$-F	CCGACGCTCGTCGAGTTCCAGCAGACGGTGCAGACGCTCG		5501
BopE$^{N224P/R230Q}$-R	CTGGAACTCGACGAGCGTCGGCGAACGCTCGGCCGGCAAC		
BopE$^{R207E/N216P}$-F	GAGCAGCAGGCGATCGATCTCGTCGCGCCGGCGTTGCCGG CCGAGC		5501
BopE$^{R207E/N216P}$-R	AGATCGATCGCCTGCTGCTCCGCATACGGCTGAAGCTCGCG		
SopD2-F	TCAGATCTGCCACCATGCCAGTTACGTTAAGTTTTGG	*Bgl* II	983
SopD2-F	GTGGATCCTTTATATAAGCATATTGCGACAACTCG	*Bam*H I	
**The primers for yeast two hybrid**			
pGBK-F1	ATGAATTCGGCCTCCATGGCCATATGCAG	*Eco*R I	7301
pGBK-R1	GAGGATCCGTCGACCTGCAGC	*Bam*H I	
pGBK-F2	ATGGTACCGAATTCGGCCTCCATGGCCATATGC	*Kpn* I	
pGAD-F	GGATCCATCGAGCTCGAGCTG		7966
pGAD-R	GAATTCACTGGCCTCCATGGC		
AD-Rab32-F	TATGGCCATGGAGGCCAGTGAATTCATGGCGGGCGAGGGA CTA	*Eco*R I	722
AD-Rab32-R	TCTGCAGCTCGAGCTCGATGGATCCTCAGCAGCACTGGGA CCTGG	*Bam*H I	
BK-BprD-F	CCGAATTCATGAAGCTCAATGGAATGACCGC	*Eco*R I	490
BK-BprD-R	ACGGATCCTCACGGCGCCGGGCGCTG	*Bam*H I	
BK-BipC-F	CCGAATTCATGTCAATCGGTGTGCAAAGCAG	*Eco*R I	1260
BK-BipC-R	ACGGATCCGGTCAGGTCCGCAGATTGCCGG	*Bam*H I	
BK-BopE-F	TCGGTACCATGACTTACAACCCGAGAATCGGC	*Kpn* I	791
BK-BopE-R	ACGGATCCTCACGCGCCGTCCGCCGCGTT	*Bam*H I	

a. The underline parts represent the enzyme cleavage sites.

Supplementary table 3. A total of 1137 differentially expressed genes (DEGs) were detected (Fold change>2, P-value < 0.05)

Gene_ID	GeneName	I_1[a]	I_2	I_3	NI_1	NI_2	NI_3	Log_2FC[b]	Diff_Type	P_Value
BPSS1496	tssB	14.37	18.4	13.23	0	0.07	0.12	7.919491	UP	6.24E-04
BPSS0243	BPSS0243	76.57	93.35	67.22	0.64	0.57	0.79	6.889595	UP	5.10E-04
BPSS1498	hcp1	27.23	37.47	34.78	0.44	0.71	0.12	6.291506	UP	4.38E-04
BPSL1775	BPSL1775	114.98	134.85	122.97	2.15	1.53	1.52	6.163746	UP	2.92E-05
BPSS1497	tssC	10.54	13.43	9.88	0.1	0.21	0.28	5.842297	UP	5.29E-04
BPSL1779	BPSL1779	67.67	72.68	71.47	1.62	1.25	1.25	5.684051	UP	1.37E-06
BPSL0024	BPSL0024	1631.93	1262.67	1401.86	36.45	28.8	19.83	5.658185	UP	2.01E-04
BPSL1776	BPSL1776	91.49	93.45	79.6	2.52	1.55	1.42	5.590536	UP	3.77E-05
BPSS1533	bicA	87.78	108.01	90.85	2.56	1.62	2.45	5.434087	UP	1.21E-04
BPSS0244	BPSS0244	44.1	50.96	47.52	0.89	1.08	1.4	5.402879	UP	1.99E-05
BPSS0362	BPSS0362	78.02	113.96	87.98	1.53	2.05	3.13	5.382764	UP	1.05E-03
BPSL1786	BPSL1786	116.68	135.25	86.26	2.38	3.29	2.48	5.37489	UP	1.53E-03
BPSS0369	BPSS0369	370.8	430.41	369.57	8.31	8.49	12.3	5.330307	UP	4.60E-05
BPSS0495	BPSS0495	23.23	23.82	24.62	0.82	0.35	0.68	5.275772	UP	6.73E-07
BPSL1785	BPSL1785	174.51	182.28	186.16	5.98	5.93	3.67	5.123052	UP	9.50E-07
BPSS1233	BPSS1233	36.73	37.6	37.02	1.53	0.54	1.24	5.072127	UP	8.21E-08
BPSS0494	BPSS0494	18.96	22.58	21.08	0.51	0.72	0.72	5.005077	UP	4.32E-05
BPSS1499	tssE	26.98	35.04	25.9	0.7	0.86	1.18	5.003944	UP	5.99E-04
BPSL1787	BPSL1787	63.2	69.09	63.33	2.12	1.17	3.04	4.949705	UP	6.21E-06
BPSL0434	BPSL0434	9.3	11.4	9.19	0	0.65	0.35	4.901591	UP	2.05E-04
BPSL1784	BPSL1784	47.75	57.06	56.79	1.81	2.04	1.76	4.848283	UP	7.04E-05
BPSS1548	bsaL	164.49	257.56	205.36	10.6	6.73	4.46	4.84767	UP	1.71E-03

BPSL1774	BPSL1774	212.81	231.12	227.16	9.76	9.05	4.88	4.824156	UP	3.04E-06
BPSS1517	BPSS1517	81.03	109.82	52.23	2.75	3.28	2.58	4.819274	UP	9.30E-03
BPSS2125	BPSS2125	224.98	214.28	237.35	7.2	8.05	9.12	4.795146	UP	5.34E-06
BPSL1777	BPSL1777	106.18	103.18	100.87	4.86	3.82	2.66	4.773846	UP	4.65E-07
BPSS1549	bsaK	44.95	67.08	42.65	2.23	1.68	1.79	4.762181	UP	3.11E-03
BPSS0357	BPSS0357	233.57	257.3	259.97	8.62	7.78	11.35	4.757946	UP	9.03E-06
BPSL3369	BPSL3369	299.47	358.1	321.76	14.46	12.75	9.19	4.749785	UP	5.22E-05
BPSL1778	BPSL1778	88.91	91.78	92.6	3.97	3.42	2.78	4.748041	UP	1.90E-07
BPSS0241	BPSS0241	12.83	13.83	10.98	0.52	0.52	0.43	4.678379	UP	1.34E-04
BPSS0242	BPSS0242	20.42	23.35	17.84	0.97	0.63	0.85	4.652311	UP	2.46E-04
BPSS1530	bprA	109.31	133.09	116.08	5.75	3.75	5.59	4.570228	UP	8.67E-05
BPSL0025	BPSL0025	362.56	312.89	324.54	15.74	13.13	13.84	4.549268	UP	2.90E-05
BPSS1235	BPSS1235	40.88	38.02	35.85	2.17	1.44	1.46	4.500365	UP	1.58E-05
BPSS1234	BPSS1234	29.12	27.46	27.75	1.9	0.76	1.19	4.453116	UP	1.60E-06
BPSS1531	bipC	91.28	117.96	103.24	5.06	4.36	5.21	4.416762	UP	2.11E-04
BPSS0367	BPSS0367	87.78	104.44	110.59	5.6	4.29	5.45	4.303042	UP	1.49E-04
BPSS1529	bipD	36.42	45.39	31.92	2.41	1.56	1.87	4.283501	UP	8.21E-04
BPSS1532	bipB	33.62	41.78	34.26	2.35	1.62	1.72	4.268465	UP	1.92E-04
BPSS1547	BPSS1547	23.37	34.4	27.38	2.45	1.07	1.12	4.19781	UP	1.18E-03
BPSS0358	BPSS0358	95.37	107.05	108.9	5.65	5.73	5.84	4.176241	UP	2.07E-05
BPSS1516	bopC	81.61	105.58	58.62	5.36	4.18	4.25	4.155849	UP	4.67E-03
BPSS1521	bprD	105.29	137.21	121.77	10	4.26	7.46	4.067912	UP	2.60E-04
BPSL1793	BPSL1793	8.97	7.15	7.84	0.34	0.27	0.83	4.056487	UP	1.78E-04
BPSS0359	BPSS0359	80.09	82.14	88.2	4.73	5.57	6.29	3.916022	UP	6.07E-06
BPSL1781	BPSL1781	27.57	32.6	39.19	3.09	2.25	1.56	3.847997	UP	8.15E-04
BPSS0324	BPSS0324	146.06	148.91	150.49	9.86	8.8	15.56	3.702384	UP	6.28E-07

BPSS1550	basJ	29.77	33.61	27.45	2.82	1.84	2.35	3.695683	UP	1.05E-04
BPSS0368	BPSS0368	24.4	25.51	28.39	2.24	2.04	1.85	3.675053	UP	3.59E-05
BPSL1742	arcD	140.09	138.66	114.85	5.22	7.2	18.44	3.67292	UP	1.90E-04
BPSS1232	BPSS1232	95.31	102.46	106.14	9.34	5.79	9.08	3.649969	UP	1.03E-05
BPSS1027	BPSS1027	79.04	92.79	92.47	5.68	6.63	9.1	3.62582	UP	6.36E-05
BPSL0826	BPSL0826	1052.28	1076.9	1235.66	127.7	71.99	74.91	3.615134	UP	6.86E-05
BPSS1525	bopE	153.47	181.01	174.51	18.73	10.36	13.11	3.592322	UP	5.66E-05
BPSS2000	BPSS2000	14.16	17.47	15.95	0.57	1.49	1.94	3.572283	UP	1.51E-04
BPSL0840	BPSL0840	79.29	70.87	82.79	8.76	4.68	6.67	3.534035	UP	4.49E-05
BPSL0503	BPSL0503	29.71	30.06	28.08	1.02	3.45	3.17	3.523398	UP	1.08E-05
BPSS0672	BPSS0672	41.79	43.62	42.59	3.05	4.61	3.49	3.521028	UP	6.40E-07
BPSL0271	flgC	8.59	8.95	6.82	0.9	0.68	0.56	3.508831	UP	3.71E-04
BPSL1274	BPSL1274	69.2	59.82	56.91	4.55	5.42	6.51	3.495971	UP	1.13E-04
BPSS1523	bicP	23.7	28.75	23.61	3.45	1.59	1.72	3.492043	UP	2.13E-04
BPSL1943	rpmI	5990.02	6992.39	7544.63	573.57	686.09	588.87	3.473075	UP	1.67E-04
BPSL0026	fliL	12.17	12.05	11.8	0.78	1.75	0.73	3.465854	UP	6.25E-06
BPSS1526	bapC	32.02	32.2	36.98	3.82	2.64	2.89	3.436099	UP	5.13E-05
BPSS1524	bopA	14.94	17.82	17.26	2.47	1.16	1.1	3.402593	UP	1.07E-04
BPSS1536	bsaX	25.64	31.69	30.96	1.76	3.28	3.31	3.402402	UP	1.74E-04
BPSL3204	rpsQ	1736.1	2141.92	1791.12	157.94	215.82	194.23	3.319192	UP	1.87E-04
BPSL1782	fhuF	35.01	34.2	35.5	4.15	3.83	2.76	3.285333	UP	6.38E-07
BPSS1527	bapB	28.59	32.11	30.51	3.53	2.84	2.99	3.284612	UP	1.25E-05
BPSS1495	virA	14.38	16.89	17.33	1.76	1.3	2.19	3.210567	UP	1.11E-04
BPSS0240	BPSS0240	8.15	7.52	6.66	0.47	1.07	0.88	3.205904	UP	1.42E-04
BPSS1528	bapA	27.48	33.47	30.37	4	2.8	3.24	3.185172	UP	1.05E-04
BPSL3205	rpmC	1860.56	2349.61	1990.08	170.66	264.11	249.06	3.180617	UP	2.49E-04

BPSS0673	BPSS0673	46.57	53.54	52.66	5.62	5.42	6.02	3.162672	UP	3.30E-05
BPSS1520	bprC	13.56	14.89	10.57	1.49	1.3	1.64	3.138835	UP	8.44E-04
BPSS1522	bprB	24.8	27.55	22.08	3.49	2.33	2.69	3.128653	UP	1.69E-04
BPSS0366	BPSS0366	74.78	83.29	86.58	10.42	9.24	8.43	3.122591	UP	3.50E-05
BPSL0829	BPSL0829	196.55	224.7	199.95	30.29	23.62	18.27	3.105387	UP	4.33E-05
BPSL0236	BPSL0236	24.56	30.03	32.31	3.21	2.48	4.77	3.054473	UP	4.43E-04
BPSS0585	BPSS0585	22.87	26.25	25.14	2.73	2.85	3.52	3.028647	UP	2.93E-05
BPSL1783	fhuB	48.01	46.71	47.1	6.96	5.73	4.69	3.028561	UP	6.77E-07
BPSS1934	BPSS1934	332.07	283.25	287.92	13.72	28.26	69.34	3.020397	UP	3.17E-04
BPSS1933	BPSS1933	219.37	166.48	153.55	7.04	17.3	43.31	2.995194	UP	2.33E-03
BPSS0938	BPSS0938	192.53	208.79	229.38	28	25.67	26.04	2.984121	UP	6.72E-05
BPSL0830	BPSL0830	143.68	158.65	135.37	23.24	18.9	13.64	2.972123	UP	6.54E-05
BPSS1545	bsaO	16.78	22.39	23.07	3.45	2.24	2.33	2.956168	UP	8.81E-04
BPSS1494	virG	22.59	24.28	21.45	2.7	2.76	3.35	2.955094	UP	1.98E-05
BPSS1932	BPSS1932	64.95	58.29	59.86	2.94	5.66	15.1	2.949673	UP	2.24E-04
BPSS2172a	BPSS2172a	148.43	167.26	165.23	24.7	22.14	16.3	2.929171	UP	2.75E-05
BPSL0501	cydB	21.9	22.04	22.67	2.6	3.1	3.07	2.92509	UP	2.94E-07
BPSS1028	BPSS1028	7.79	9.55	10.22	0.9	0.87	1.86	2.924534	UP	5.53E-04
BPSL0502	BPSL0502	25.6	28.32	25.78	3.53	3.06	4.12	2.895621	UP	1.59E-05
BPSL0520	BPSL0520	1598.61	1917.95	1959.27	243.58	272.1	228.68	2.879005	UP	1.62E-04
BPSS2349	BPSS2349	49.41	72.24	52.71	6.74	7.79	9.91	2.834753	UP	2.25E-03
BPSS1493	tssA	15.91	17.22	16.31	2.47	2.2	2.29	2.82852	UP	3.64E-06
BPSS0831	BPSS0831	556.17	467.77	512.19	29.09	61	128.8	2.811022	UP	3.52E-04
BPSS0077A	BPSS0077A	6.31	11.66	7.87	0.73	1.19	1.77	2.807913	UP	1.03E-02
BPSS2228	BPSS2228	19.48	19.49	15.92	3.04	2.14	2.71	2.798446	UP	2.10E-04
BPSS0360	BPSS0360	59.09	66.94	81.48	9.02	9.42	11.44	2.795929	UP	8.55E-04

BPSL1947	BPSL1947	239.33	269.67	240.55	34.33	35.34	41.53	2.752868	UP	3.08E-05
BPSL3206	rplP	3285.46	3470.18	2760.5	447.69	582.29	393.47	2.740985	UP	2.53E-04
BPSS1236	ugpC	27.18	24.24	24.7	3.93	2.77	4.75	2.732928	UP	3.70E-05
BPSS1335	BPSS1335	44.05	41.37	36.57	3.17	4.61	10.75	2.718828	UP	4.16E-04
BPSL2308	BPSL2308	11.41	13.2	9.45	1.95	0.99	2.26	2.711495	UP	1.11E-03
BPSS0674	BPSS0674	7.05	8.12	10.75	1.38	1.32	1.39	2.663893	UP	2.70E-03
BPSS1112	arcD	60.67	45.39	43.01	4.12	6.8	12.61	2.663417	UP	2.33E-03
BPSL2307	BPSL2307	9.94	9.43	6.64	1.48	1.05	1.6	2.654853	UP	2.17E-03
BPSS1537	spaP	13.68	21.31	16.33	3.4	2.19	2.57	2.65288	UP	3.15E-03
BPSL3215	tuf	22.78	27.59	21.61	2.94	3.7	4.81	2.652249	UP	4.53E-04
BPSL0827	BPSL0827	362.9	354.02	424.82	93.1	51.8	38.6	2.637382	UP	3.21E-04
BPSS1044	BPSS1044	28.35	24.92	30.13	4.1	4.38	5.13	2.61538	UP	1.18E-04
BPSS0141	BPSS0141	56.07	77.16	74.2	11.28	11.53	11.29	2.604781	UP	9.35E-04
BPSL3311	flhD	102.89	98.6	85.38	14.73	16.77	15.77	2.6014	UP	1.13E-04
BPSS1163	fnr	751.17	792.53	766.11	101.41	138.58	141.14	2.599419	UP	3.38E-06
BPSL1621	BPSL1621	5.98	8.55	6.16	1.19	1.55	0.68	2.596865	UP	2.66E-03
BPSS0233	BPSS0233	10.66	10.52	13.36	1.31	1.54	2.86	2.596705	UP	7.70E-04
BPSS0322	BPSS0322	240.84	244.57	277.33	32.53	34.29	59.42	2.595022	UP	1.26E-04
BPSS0481	BPSS0481	110.88	111.58	102.61	22.86	15.08	16.64	2.574306	UP	1.75E-05
BPSS1595	pilS	44.98	50.55	48.04	10.6	7.47	6.19	2.565103	UP	4.37E-05
BPSL2911	rplM	4122.97	5010.64	4736.59	646.45	840.51	882.21	2.549535	UP	1.48E-04
BPSS1553	bprP	25.65	33.17	32.45	4.33	4.9	6.38	2.54767	UP	5.20E-04
BPSL1406	BPSL1406	519.02	488.95	387.66	35.56	66.24	138.77	2.536388	UP	1.55E-03
BPSL3191	rpmJ	760.9	906.47	738.71	86.57	154.81	173.56	2.53571	UP	3.53E-04
BPSL2378	cyoA	5.02	5.19	4.49	0.32	0.53	1.69	2.532916	UP	1.04E-03
BPSL3200	rpsN	802.83	927.24	828.25	113.49	151.42	179.19	2.52624	UP	7.72E-05

BPSL2743	BPSL2743	105.77	99.95	110.25	12.27	13.76	29.09	2.51914	UP	1.45E-04
BPSL0027	fliM	11.94	11.39	12.76	2.19	2.37	1.75	2.515887	UP	2.26E-05
BPSS1551	BPSS1551	7.03	8.64	7.59	1.59	0.86	1.64	2.507678	UP	2.82E-04
BPSL2377	BPSL2377	11.21	8.57	9.14	0.6	1.53	2.97	2.503498	UP	1.68E-03
BPSL1743	arcA	93.51	83.43	63.1	10.21	10.01	22.29	2.497401	UP	2.58E-03
BPSL1942	rplT	4059.91	4159.86	3898.84	639.68	727.92	792.04	2.488361	UP	2.94E-06
BPSS0840	BPSS0840	55.32	44.73	52.65	3.24	5.28	19.1	2.466915	UP	2.13E-03
BPSL0270	flgB	17.56	12.74	14.47	2.68	3.03	2.45	2.455891	UP	1.01E-03
BPSL3209	rpsS	1817.02	1973.32	1723.41	286.12	400.87	318.75	2.454776	UP	4.85E-05
BPSL2318	glnA	731.08	746.1	688.69	121.64	122.34	154.21	2.443418	UP	8.34E-06
BPSL3310	flhC	80.15	70.8	68.06	11.77	16.27	12.23	2.443219	UP	1.10E-04
BPSS1204	BPSS1204	16.51	17.66	19.33	2.79	3.06	4.05	2.434038	UP	8.75E-05
BPSL3207	rpsC	2744.28	2702.06	2208.52	436.22	575.49	410.44	2.428302	UP	3.18E-04
BPSL1185	BPSL1185	25.96	21.94	19.64	3.86	3.66	5.05	2.425758	UP	6.43E-04
BPSS1954	BPSS1954	218.89	204.6	192.21	11.54	27.29	75.77	2.425621	UP	1.31E-03
BPSL0831	BPSL0831	185.51	182.37	177.16	41.13	32.01	28.63	2.42105	UP	4.94E-06
BPSS0325	BPSS0325	76.34	87.81	83.91	14.24	14.37	17.89	2.415386	UP	4.71E-05
BPSL1491	BPSL1491	837.02	1113.97	1158.65	120.94	203.48	260.2	2.411176	UP	1.48E-03
BPSL2159	rpsB	642.27	853.21	722.82	108.35	130.02	178.9	2.410401	UP	7.59E-04
BPSL1792	BPSL1792	8.45	6.86	8.19	1.86	1.33	1.27	2.397545	UP	2.72E-04
BPSL0871	rpsT	567.75	756.19	644.44	99.57	132.45	142.11	2.395397	UP	6.98E-04
BPSS1928	BPSS1928	109.25	85.94	95.09	7.73	12.43	35.08	2.39366	UP	1.94E-03
BPSL3218	rpsL	687.83	710.81	557.59	113.59	128.97	132.53	2.382767	UP	3.93E-04
BPSS1543	bsaQ	5.56	7.47	6.44	1.27	0.72	1.8	2.360983	UP	1.18E-03
BPSS1542	bsaR	44.75	46.37	41.83	9.62	8.74	7.68	2.352082	UP	1.59E-05
BPSS1534	bsaZ	5.89	7.35	6.82	1.38	1.33	1.22	2.35172	UP	2.34E-04

BPSS0485	BPSS0485	42.16	41.68	36.68	9.45	7.84	6.36	2.34936	UP	8.05E-05
BPSS0578	livG	52.22	65.33	69.07	12.13	13.11	11.52	2.343895	UP	6.23E-04
BPSS2173	BPSS2173	70.54	81.95	69.79	16.15	16.11	11.71	2.337787	UP	1.45E-04
BPSL1339	proP	199.79	236.67	225.17	34.59	37.28	60.99	2.316118	UP	2.13E-04
BPSL0303	BPSL0303	114.82	98.66	99.35	10.19	14.82	38.12	2.308981	UP	1.19E-03
BPSL3208	rplV	1705.18	1786.45	1407.5	271.85	394.24	324.84	2.305671	UP	4.15E-04
BPSL0030	fliP	5.42	6.31	6.27	1.18	1.05	1.42	2.302029	UP	1.03E-04
BPSL3202	rplX	461.33	578.51	469.8	75.75	100.87	129.87	2.300293	UP	6.03E-04
BPSL1552	BPSL1552	801.41	729.2	617.9	55.63	114.08	267.74	2.296146	UP	2.32E-03
BPSS1999	BPSS1999	6.39	8.4	7.95	1.46	1.39	1.8	2.28993	UP	6.34E-04
BPSS0265	BPSS0265	33.02	34.13	32.45	7.14	5.25	8.13	2.279115	UP	1.13E-05
BPSL0435	BPSL0435	18.75	15.73	17.53	4.06	3.32	3.41	2.269094	UP	1.11E-04
BPSS1310	BPSS1310	90.19	92.19	72.94	15.18	16.49	21.54	2.262537	UP	4.64E-04
BPSL3211	rplW	422	438.53	362.28	71.96	93.06	89.91	2.262027	UP	1.79E-04
BPSL0273	flgE	17.67	20.28	16.19	3.98	3.93	3.4	2.259096	UP	2.95E-04
BPSL1247	BPSL1247	113.37	98.81	110.33	22.13	20.85	24.45	2.257881	UP	4.86E-05
BPSL0230	fliF	8.46	8.36	7.96	1.93	1.6	1.67	2.252593	UP	3.67E-06
BPSL0674	miaB	71.45	81.93	75.79	14.18	14.24	19.91	2.245427	UP	7.33E-05
BPSS0486	BPSS0486	56.97	60.1	58.65	14.28	10.58	12.31	2.241068	UP	5.04E-06
BPSS1936	BPSS1936	149.79	137.86	119.93	8.7	22.8	55.05	2.235478	UP	2.74E-03
BPSS1921	BPSS1921	309.94	259.67	237.28	26.57	50.51	95.61	2.224187	UP	2.01E-03
BPSS2302	BPSS2302	32.07	28.99	31.8	2.82	4.8	12.29	2.221564	UP	1.34E-03
BPSS1774A	BPSS1774A	8.47	7.21	8.06	1.25	2.22	1.67	2.20748	UP	1.83E-04
BPSL1791	BPSL1791	6.01	5.17	5.66	1.35	0.97	1.36	2.194114	UP	9.08E-05
BPSS1540	bsaT	5.68	7.32	7.54	2.21	0.47	1.81	2.193649	UP	2.46E-03
BPSL2317	glnL	64.76	59.99	63.44	12.97	11.1	17.08	2.193226	UP	2.71E-05

BPSS0760	BPSS0760	39.64	43.03	42.41	10.23	8.67	8.5	2.190603	UP	1.02E-05
BPSS1546	bsaN	20.84	27.41	18.81	6.11	4.08	4.51	2.189636	UP	2.82E-03
BPSL0915	rpmG	628.92	911.38	734.28	117.71	150.46	232.78	2.182862	UP	2.69E-03
BPSL0916	rpmB	3113.24	3894.3	3023.55	652.55	716.69	843.33	2.180683	UP	7.63E-04
BPSS0487	BPSS0487	82.09	78.88	75.84	21.74	14.68	15.91	2.17802	UP	2.65E-05
BPSS1923	BPSS1923	114.36	97.67	74.33	23.37	23.6	16.45	2.17482	UP	3.29E-03
BPSS1267	BPSS1267	8.83	8.49	6.67	1.71	2.46	1.16	2.170226	UP	1.27E-03
BPSL0272	flgD	10.98	10.47	11.14	2.87	1.97	2.41	2.168376	UP	1.37E-05
BPSL1474	BPSL1474	50.07	59.53	55.36	10.57	12.66	13.73	2.15808	UP	1.23E-04
BPSS0323	BPSS0323	188.55	164.63	183.67	32.59	29.4	58.35	2.157403	UP	2.90E-04
BPSL3217	rpsG	466.78	533.1	396.17	94.01	99.31	119.87	2.156241	UP	8.61E-04
BPSS0484	BPSS0484	91.49	77.32	79.07	23.62	17.33	14.72	2.15467	UP	2.46E-04
BPSL1794	BPSL1794	28.95	23.86	28.58	7.02	6.32	5.17	2.136547	UP	2.63E-04
BPSL0226	fliJ	6.43	6.14	7.11	1.56	1.76	1.2	2.122336	UP	1.07E-04
BPSS0483	BPSS0483	72.95	67.07	59.62	19.58	13.21	13.12	2.12052	UP	3.14E-04
BPSL3188	rpsD	1145.26	1242.32	1007.52	223.22	326.52	234.37	2.114326	UP	3.25E-04
BPSL2910	rpsI	1981.17	2202.65	2029.84	438.86	488.65	508.58	2.113297	UP	2.25E-05
BPSS0321	BPSS0321	210.73	187.05	229.51	49.17	44.93	51.22	2.109899	UP	2.06E-04
BPSL3221	rpoB	287.16	301.32	236.22	55.81	78.96	56.57	2.107731	UP	5.69E-04
BPSS0590	BPSS0590	5.69	5.39	4.52	1.14	1.2	1.28	2.107484	UP	3.49E-04
BPSL0828	BPSL0828	183.41	178.23	187.11	62.76	36.15	28.98	2.101246	UP	1.88E-04
BPSL3223	rplJ	3743.75	3143.52	2748.27	696.09	857.13	694.76	2.099736	UP	1.12E-03
BPSL3186	rplQ	919.86	1002.62	889.83	200.64	218.32	240.42	2.092574	UP	3.59E-05
BPSL1911	typA	133.68	149.53	143.95	28.28	33.18	39.38	2.082709	UP	4.27E-05
BPSL3158	BPSL3158	184.78	175.32	214.62	37.26	48.77	49.74	2.081695	UP	3.05E-04
BPSL3203	rplN	1441.14	1639.75	1557.35	291.39	347.94	458.54	2.07887	UP	9.88E-05

BPSL1917	rbfA	249.62	259.59	266.66	56.81	69.1	59.44	2.065563	UP	5.85E-06
BPSL2283	iscX	158.73	161.53	142.64	29.71	47.63	33.29	2.064958	UP	1.28E-04
BPSL3401	atpE	1349.38	1564.1	1173.16	271.64	337.17	379.84	2.047383	UP	9.19E-04
BPSL2148	fabZ	138.83	155.29	131.41	29.75	35.67	38.49	2.033926	UP	1.40E-04
BPSS1943	BPSS1943	43.69	35.88	31.9	4.53	3.68	19.01	2.033917	UP	9.78E-03
BPSL1009	BPSL1009	190.92	174.88	158.48	36.92	43.38	48.76	2.022296	UP	1.90E-04
BPSS1539	bsaU	5.35	6.9	6.59	2.19	1.37	1.09	2.018496	UP	1.21E-03
BPSS1576	bcsG	12.88	14.08	17.81	3.39	3.57	4.14	2.011973	UP	1.71E-03
BPSL2379	cyoB	22.64	17.01	17.89	3.06	3.55	7.66	2.01158	UP	3.18E-03
BPSL1852	dusA	70.11	82.94	79.6	15.95	17.19	24.76	2.006526	UP	2.49E-04
BPSS0140	BPSS0140	132.29	174.37	161.61	39.14	36.37	41.12	2.005402	UP	7.28E-04
BPSL3396	atpD	990.73	1021.22	893.99	225.7	295.87	204.01	2.001798	UP	1.05E-04
BPSL2363	BPSL2363	16.13	12.05	14.51	2.09	2.51	6.08	1.998987	UP	3.54E-03
BPSL0275	flgG	11.46	11.31	13.1	3.22	2.4	3.36	1.99799	UP	1.57E-04
BPSL3237	BPSL3237	177.27	163.63	156.88	32.35	41.37	50.96	1.997278	UP	1.03E-04
BPSL3220	rpoC	535.43	581.97	514.16	115.05	157.97	136.42	1.994528	UP	6.55E-05
BPSS1535	bsaY	10.08	11.37	10.59	2.94	1.92	3.19	1.992813	UP	1.21E-04
BPSL3192	infA	889.28	1026.68	849.61	163.7	301.43	230.84	1.99048	UP	4.95E-04
BPSL3395	BPSL3395	1438.05	1415.4	1274.5	291.27	446.44	303.26	1.987497	UP	1.35E-04
BPSL3210	rplB	1381.5	1363.75	1298.83	310.93	382.24	327.12	1.986832	UP	6.95E-06
BPSL0446	secB	1062.15	1215.85	1103.68	245.44	278.69	329.91	1.985365	UP	8.53E-05
BPSL1741	BPSL1741	199.18	173.61	183.31	18.15	33.42	89.21	1.981902	UP	3.74E-03
BPSS1115	BPSS1115	717.01	601.03	544.71	60.55	149.38	261.93	1.981003	UP	3.88E-03
BPSL3005	rplU	2908.65	3561.68	3289.14	618.03	1053.24	802.26	1.980231	UP	4.36E-04
BPSL2515	rpsA	1652.71	1764.52	1674.23	370.06	432.25	502.59	1.96414	UP	1.63E-05
BPSL3190	rpsM	1808	1809.15	1341.32	335.16	531.35	410.6	1.957012	UP	1.78E-03

BPSS2038	BPSS2038	108.64	117.88	110.33	26.29	25.4	35.1	1.956506	UP	3.81E-05
BPSS0588	BPSS0588	5.39	4.63	4.29	1.07	0.86	1.77	1.951426	UP	1.15E-03
BPSL2284	fdx	116.09	118.46	93.39	25.85	35.63	23.61	1.94637	UP	7.76E-04
BPSL2239	BPSL2239	265.08	302.32	274.8	59.99	71.52	87.23	1.944946	UP	1.09E-04
BPSL0241	BPSL0241	435.64	480.16	469.71	104.51	108.17	147.24	1.944669	UP	5.82E-05
BPSL3189	rpsK	1513.54	1524.11	1280.74	354.57	451.11	316.42	1.944292	UP	2.79E-04
BPSL3216	fusA	212.1	224.28	179.14	51.19	64.15	44.61	1.944185	UP	4.90E-04
BPSL1221	nuoK	307.76	326.01	274.35	58.21	114.58	64	1.939275	UP	6.72E-04
BPSL3368	BPSL3368	18.1	21.3	22.43	6.66	4.81	4.69	1.93588	UP	4.59E-04
BPSL1000	apbC	153.98	169.81	162.53	35.97	40.91	50.24	1.935715	UP	4.24E-05
BPSL0028	fliN	24.35	23.28	27.66	6.98	7.3	5.45	1.932067	UP	2.09E-04
BPSL1256	BPSL1256	24.38	19.94	20.49	4.21	6.9	5.88	1.931531	UP	5.73E-04
BPSS1286	BPSS1286	80.32	92.42	73.48	20.4	22.49	21.71	1.930342	UP	4.07E-04
BPSL1458	rpsF	1830.47	2335.91	1935.23	393.05	595.81	615.9	1.926832	UP	9.06E-04
BPSS1538	bsaV	13.21	11.98	11.95	4.13	2.62	3.04	1.923593	UP	1.18E-04
BPSL1349	carA	169.76	179.29	157.91	41.07	40.75	51.95	1.922117	UP	6.57E-05
BPSS1958	BPSS1958	257.52	212.05	194.74	20.69	38.73	116.14	1.919892	UP	9.39E-03
BPSL0833	ugpC	180.61	175.13	178.72	61.27	44.21	35.84	1.919116	UP	6.82E-05
BPSL0780	sucD	427.02	500.47	414.53	119.87	124.74	110.26	1.919044	UP	2.66E-04
BPSL1219	nuoI	111.05	120.38	109.33	28.41	33.84	28.1	1.915159	UP	2.84E-05
BPSS1162	BPSS1162	858.84	996.94	941.18	190.11	246.28	306.56	1.912523	UP	1.97E-04
BPSL2932	edd	27.46	33.76	35.14	9.64	7.97	8	1.911727	UP	6.28E-04
BPSS0761	codA	49.23	51.11	56.56	15.14	14.38	12.27	1.908616	UP	8.37E-05
BPSS1101	BPSS1101	398.11	340.15	349.72	38.32	62.29	191.06	1.899243	UP	6.36E-03
BPSS1514	BPSS1514	9.42	13.75	10.41	2.49	2.81	3.71	1.898003	UP	3.83E-03
BPSL3225	rplK	1797.53	1850.52	1650.13	395.3	512.39	514.27	1.897616	UP	5.58E-05

BPSL3201	rplE	1061.63	1064.54	833.71	231.99	271.44	292.02	1.895696	UP	7.77E-04
BPSS0880	BPSS0880	626.15	749.6	720.04	133.69	166.45	264.43	1.89227	UP	7.04E-04
BPSL1761	BPSL1761	57.84	52.8	55.35	15.53	15.44	13.85	1.888882	UP	1.30E-05
BPSL1408	BPSL1408	20.88	20.61	20.53	0.88	2.99	12.93	1.884272	UP	1.54E-02
BPSL1499	BPSL1499	97.07	121.75	108.5	19.37	23.25	46.25	1.880933	UP	1.95E-03
BPSL0665	BPSL0665	32.14	36.94	38.04	9.81	9.46	9.89	1.877165	UP	1.39E-04
BPSS1592	sctC	6.36	7.44	9.28	1.69	2.07	2.54	1.873219	UP	3.24E-03
BPSL0087	BPSL0087	30.89	38.21	26.93	6.78	8.01	11.43	1.872817	UP	2.90E-03
BPSL3222	rplL	1409.5	1529.26	1409.59	336.2	424.81	429.19	1.869264	UP	3.03E-05
BPSL2984	accC	128.67	145.87	130.58	33.66	37.81	39.51	1.86805	UP	6.77E-05
BPSS1364a	rpsU	155.22	179.93	127.67	41.66	40.64	45.26	1.859275	UP	1.80E-03
BPSL2931	eda	154.58	181.73	194.49	54.72	51.63	40.55	1.853334	UP	5.18E-04
BPSL2029	BPSL2029	13.62	12.98	17.4	6.06	2.01	4.13	1.850622	UP	4.23E-03
BPSL0734	BPSL0734	26.57	27.37	25.38	6.42	4.48	11.21	1.842986	UP	7.89E-04
BPSL3400	BPSL3400	808.56	924.74	831.36	204.12	251.51	259.7	1.842087	UP	9.88E-05
BPSL2398	hpnJ	111.39	124.65	115.06	25.96	30.31	42.24	1.83354	UP	1.78E-04
BPSL2309	BPSL2309	20.05	24.01	18.42	6.02	2.71	8.81	1.832746	UP	3.47E-03
BPSS1924	BPSS1924	129.78	124.47	119.93	10.92	22.8	71.36	1.832244	UP	8.68E-03
BPSS1996	BPSS1996	40.21	49.47	35.2	10.72	13.08	11.45	1.824847	UP	2.13E-03
BPSL3336	BPSL3336	62.5	62.67	58.6	20.73	14.86	16.53	1.817992	UP	3.69E-05
BPSS1541	bsaS	17.12	21.76	19.32	6.64	5.59	4.28	1.817679	UP	7.62E-04
BPSS0234	cydB	229.87	187.72	201.97	41.31	52.25	82.26	1.817145	UP	1.05E-03
BPSL3196	rpsE	1347.98	1356.65	1215.32	329.53	422.85	360.73	1.816239	UP	6.20E-05
BPSL1945	thrS	199.86	220.85	194.44	46.42	55.32	73.49	1.811688	UP	2.05E-04
BPSL2031	BPSL2031	8.5	9.35	9.34	2.88	3	1.88	1.808948	UP	1.39E-04
BPSS1139	BPSS1139	9.06	7.02	6.58	2.24	1.69	2.57	1.801636	UP	2.61E-03

BPSL0294	BPSL0294	15.87	18.45	17.53	5.2	4.92	4.77	1.8	UP	8.71E-05
BPSL2187	BPSL2187	563.88	538.6	492	121.71	134.18	202.13	1.799603	UP	3.16E-04
BPSL0229	fliG	17.11	16.17	17.35	5.07	4.35	5.2	1.792049	UP	1.14E-05
BPSS0557	BPSS0557	179.67	203.39	205.15	48.02	43.64	78.45	1.789863	UP	5.24E-04
BPSL2413	BPSL2413	173.7	177.65	163.89	45.17	53.13	50.82	1.788771	UP	1.33E-05
BPSS2348	arsC	33.78	40.51	31.64	8.38	10.7	11.65	1.785392	UP	9.14E-04
BPSS1937	BPSS1937	179.82	161.34	154.09	14.91	31.22	97.74	1.783391	UP	1.15E-02
BPSL3195	rpmD	1141.04	1050.68	871.45	278.49	362.12	249.92	1.782289	UP	1.09E-03
BPSL1851	BPSL1851	40.07	41.61	40.63	10.2	13.15	12.21	1.782215	UP	7.84E-06
BPSL3212	rplD	608.4	555	486.13	142.34	161.18	177.08	1.779146	UP	4.50E-04
BPSS0235	BPSS0235	247.87	221.47	229.16	56.52	59.54	87.47	1.779019	UP	1.96E-04
BPSL2489	rplS	2967.6	2825.02	2379.19	623.87	858.35	903.23	1.776394	UP	6.13E-04
BPSL0911	BPSL0911	686.57	780.53	798.84	164.37	211.63	289.61	1.767361	UP	4.52E-04
BPSL3187	rpoA	1201.55	1255.68	1050.45	309.51	416.07	306.15	1.765452	UP	3.17E-04
BPSS1779	BPSS1779	68.02	72.35	81.23	19.17	20.2	25.9	1.763466	UP	2.95E-04
BPSL2741	BPSL2741	44.29	33.34	37.4	8.21	9.45	16.43	1.75459	UP	2.75E-03
BPSS0320	BPSS0320	184.15	201.1	215.05	54.53	55.32	68.12	1.75405	UP	1.45E-04
BPSL1475	BPSL1475	70.73	75.39	88.47	24.27	20.08	25.23	1.753397	UP	5.78E-04
BPSL2370	sufC	5.14	3.71	3.31	1.04	1.32	1.25	1.752072	UP	7.12E-03
BPSS1147	hyfB	147.37	122.79	140.94	27.92	35.12	59.12	1.750717	UP	1.29E-03
BPSL1223	BPSL1223	210.64	219.65	212.87	59.22	73.76	58.19	1.750322	UP	1.23E-05
BPSS1729	BPSS1729	42.31	37.49	43.48	8.88	9.23	18.55	1.74966	UP	1.39E-03
BPSS1152	BPSS1152	15.47	23.57	19.08	5.14	5.31	6.88	1.745763	UP	4.84E-03
BPSS0839	BPSS0839	631.18	532.48	547	55.01	138.6	316.56	1.745503	UP	8.53E-03
BPSL0302	BPSL0302	39.79	33.12	34.04	2.95	6.96	22.04	1.743049	UP	1.55E-02
BPSL1323	BPSL1323	872.81	818.61	942.98	262.15	261.15	264.83	1.740969	UP	6.88E-05

BPSL1476	BPSL1476	296.98	324.92	341.7	72.69	90	125.86	1.739613	UP	3.82E-04
BPSS1239	BPSS1239	6.66	6.18	5.48	2.02	1.6	1.87	1.738541	UP	3.00E-04
BPSL3228	tuf	85.44	105.96	90.11	20.6	23.76	40.02	1.738213	UP	1.61E-03
BPSL2362	BPSL2362	7.59	5.59	7.8	1.18	1.79	3.32	1.737883	UP	6.71E-03
BPSS0832	BPSS0832	41.23	33.78	32.69	5.36	9.73	17.31	1.732953	UP	4.68E-03
BPSL0779	sucC	311.01	325.22	260.44	91.48	90.1	88.29	1.732313	UP	4.46E-04
BPSS1953	atpD	324.68	284	312.6	31.67	64.78	181.1	1.730892	UP	1.02E-02
BPSL3193	secY	1603.7	1556.91	1507.3	425.21	542.4	439.64	1.729898	UP	1.94E-05
BPSL3105	BPSL3105	991.47	1087.38	1106.76	336.21	343.86	284.07	1.724255	UP	5.16E-05
BPSL3243	BPSL3243	264.64	314.97	267.08	63.79	79.2	113.85	1.720964	UP	8.79E-04
BPSL0824	BPSL0824	33.69	35.12	37.73	12.22	10.35	9.76	1.72045	UP	5.96E-05
BPSL3000	BPSL3000	45.41	56.91	61.48	15.1	14.47	20.2	1.718587	UP	1.75E-03
BPSL1460	rpsR	1592.16	1694.62	1405.39	346.94	480.2	605.4	1.71168	UP	6.51E-04
BPSL1471	BPSL1471	98.47	112.72	112.3	28.77	33.85	36.35	1.708658	UP	1.34E-04
BPSL2422	efp	549.78	682.91	534.79	142.45	168.62	229.69	1.708634	UP	1.60E-03
BPSS0482	BPSS0482	64.37	57.36	48.68	23.93	15.19	13.05	1.707718	UP	2.19E-03
BPSS0834	BPSS0834	110.92	111.99	111.45	16.82	24.09	61.53	1.706623	UP	5.06E-03
BPSL0192	BPSL0192	5.6	5.38	6.52	2.65	1.53	1.19	1.704361	UP	1.98E-03
BPSL3199	rpsH	389.74	397.64	316.25	81.85	116.46	140.6	1.703282	UP	1.20E-03
BPSL1207	pnp	341.07	370.23	304.18	99.73	114.53	97.64	1.703006	UP	2.93E-04
BPSL1459	priB	1249.9	1529.41	1244.92	367.2	449.18	426.82	1.694654	UP	6.73E-04
BPSL3402	atpB	460.02	506.42	478.17	142.73	156.11	149.72	1.687307	UP	1.90E-05
BPSL1772	cobO	84.94	93.01	83.26	25.97	28.35	27.18	1.680338	UP	4.16E-05
BPSL0023	BPSL0023	52.61	50.81	50.47	15.59	13.54	19.04	1.675693	UP	3.48E-05
BPSS2040	BPSS2040	35.41	36.79	36.97	11.86	9.12	13.37	1.668194	UP	4.88E-05
BPSL1548	BPSL1548	100.03	123.82	120.59	28.26	36.04	44.36	1.664432	UP	8.62E-04

BPSL2299	lpdA	120.25	121.9	124.75	40.27	37.5	38.03	1.663752	UP	7.31E-07
BPSL2452	BPSL2452	182.56	195.84	177.2	57.21	60.09	58.16	1.662904	UP	2.27E-05
BPSS0721	fabI	61.04	50.58	42.8	13.38	10.17	25.22	1.662794	UP	7.29E-03
BPSL3330	BPSL3330	22.78	27.94	23.14	7.32	7.42	8.59	1.662607	UP	5.99E-04
BPSS1780	BPSS1780	77.5	78.23	65.42	21.87	21.83	26.39	1.657745	UP	3.39E-04
BPSL3213	rplC	953.09	998.1	965.01	258.01	320.56	348.21	1.653791	UP	2.44E-05
BPSS0579	BPSS0579	52.7	68.03	75	19.83	17.87	24.55	1.652719	UP	2.94E-03
BPSS1931	BPSS1931	51.22	46.77	56.4	9.03	11.09	29.23	1.645457	UP	7.46E-03
BPSL1941	pheS	98.05	107.55	95.02	29.49	33.82	32.86	1.644282	UP	6.93E-05
BPSL3335	BPSL3335	13.11	13.92	11.72	5.4	2.8	4.21	1.642693	UP	8.88E-04
BPSS2039	BPSS2039	73.47	75.51	80.84	26.49	21.26	26.01	1.639594	UP	4.66E-05
BPSS0688	bprR	16.91	13.09	13.71	5.29	4.42	4.33	1.63842	UP	1.27E-03
BPSL1213	BPSL1213	151.23	157.68	145.45	47.35	49.94	48.66	1.638362	UP	9.05E-06
BPSL2356	BPSL2356	5.74	5.92	5.15	1.31	1.68	2.41	1.638288	UP	6.71E-04
BPSL2245	BPSL2245	504.78	582.6	547.4	182.76	163.99	178.46	1.63813	UP	9.06E-05
BPSL1031	BPSL1031	39.23	46.82	47.46	13.1	14.62	15.19	1.637562	UP	3.72E-04
BPSS1544	bsaP	7.07	10.5	7.39	3.51	1.74	2.83	1.627191	UP	9.64E-03
BPSL1222	nuoL	199.75	208.81	206.91	64.64	78.61	56.36	1.624505	UP	3.96E-05
BPSL2030	BPSL2030	18.48	19.61	20.19	8.43	5.64	4.84	1.623852	UP	3.95E-04
BPSL0190	BPSL0190	99.57	101.93	109.34	30.28	38.1	32.78	1.619533	UP	4.82E-05
BPSL1407	BPSL1407	189.43	145.62	138.56	18.77	41.95	93.49	1.618803	UP	1.74E-02
BPSL3333	BPSL3333	88.34	95.39	91.18	31.73	25.69	32.38	1.614172	UP	3.11E-05
BPSS1150	BPSS1150	66.85	48.47	43.96	13.55	14.77	23.78	1.61221	UP	9.77E-03
BPSL2481	orn	155.46	193.97	198.94	44.57	53.78	81.24	1.610443	UP	2.21E-03
BPSL3224	rplA	1559.78	1474.62	1327.29	420.39	509.82	499.64	1.609023	UP	1.86E-04
BPSL2366	hemN	177.8	134.86	152.63	39.77	47.92	64.86	1.608848	UP	1.97E-03

BPSL1226	BPSL1226	118.08	128.19	130.79	36.29	44.68	42.72	1.608065	UP	5.32E-05
BPSL2246	BPSL2246	709.95	855.22	761.48	225.14	291.12	248.62	1.604949	UP	3.69E-04
BPSL0191	BPSL0191	40.51	41.47	33.99	12.72	12.97	12.5	1.602485	UP	3.86E-04
BPSL1551	BPSL1551	469.26	441.41	448.94	66.95	122.3	258.53	1.602331	UP	6.16E-03
BPSL2895	BPSL2895	102.64	127.86	119.84	26.65	33.42	55.37	1.601613	UP	2.37E-03
BPSS2284	BPSS2284	160.42	135.69	120.02	12.62	30.8	94.3	1.595296	UP	2.76E-02
BPSS0319	BPSS0319	89.32	91.81	83.62	29.83	24.38	34.01	1.585453	UP	9.08E-05
BPSL2871	secD	153.37	168.42	163.86	50.05	55	56.9	1.584368	UP	2.52E-05
BPSL0589	BPSL0589	5.12	3.31	3.23	0.18	1.14	2.57	1.583726	UP	4.94E-02
BPSS0142	BPSS0142	30.12	39.98	37.09	11.59	10.97	13.26	1.581333	UP	1.38E-03
BPSS0576	livH	47.62	56.83	48.32	16.65	17.34	17.09	1.580531	UP	3.35E-04
BPSL3197	rplR	1363.17	1205.98	1155.97	372.23	454.73	420.15	1.578698	UP	2.47E-04
BPSL1224	nuoN	187.5	190.07	188.74	60.42	72.89	56.58	1.576428	UP	1.47E-05
BPSS1578a	BPSS1578a	15.42	11.8	10.47	3.47	4.1	5.09	1.573904	UP	5.79E-03
BPSS2041	BPSS2041	26.55	26.92	24.5	7.93	7.66	10.63	1.572251	UP	1.41E-04
BPSL2501	BPSL2501	45.13	48.14	49.35	13.63	12.54	21.83	1.57107	UP	5.87E-04
BPSL2463	BPSL2463	71.62	93.35	77.59	23.03	25.71	32.92	1.57064	UP	1.67E-03
BPSL1495	rho	566.61	650.1	569.46	179.48	182.11	240.5	1.568818	UP	3.09E-04
BPSL3398	atpA	1007.64	1018.11	936.44	325.73	428.71	248.25	1.562789	UP	3.60E-04
BPSS1993	BPSS1993	89.73	89.84	90.5	31.28	27.36	32.9	1.560859	UP	3.63E-06
BPSS0860	fliD	265.51	299.56	286.05	81.76	69.02	138.07	1.559042	UP	1.32E-03
BPSL1477	BPSL1477	174.19	175.2	203.39	51.34	53.04	83.24	1.558892	UP	9.91E-04
BPSL3159	BPSL3159	98.08	103.93	116.75	33.18	33.5	41.62	1.557437	UP	3.40E-04
BPSS2286	BPSS2286	22.21	18.32	17.39	2.79	2.83	14.06	1.557331	UP	3.41E-02
BPSS2037	BPSS2037	25.2	26.25	28.47	9.54	6.57	11.06	1.556542	UP	4.22E-04
BPSL2983	accB	132.36	137.08	101.52	40.83	40.9	45.13	1.548026	UP	1.93E-03

BPSS1704	asd	234.6	260.11	255.46	65.08	80.72	111.6	1.543206	UP	4.79E-04
BPSL2869	tgt	109.63	113.14	119.74	37.51	37.1	43.05	1.541522	UP	2.91E-05
BPSL1918	infB	545.03	535.89	566.65	173.58	188.44	206.04	1.536225	UP	1.04E-05
BPSL0187	gatC	40.09	43.7	37.77	8.36	11.52	22.09	1.534238	UP	4.13E-03
BPSL0668	glyQ	91.95	111.14	103.94	29.63	28.87	47.64	1.532411	UP	1.28E-03
BPSL2129	guaB	428.68	437.08	421.57	128.57	159.53	157.3	1.531208	UP	1.37E-05
BPSL1206	rpsO	888.29	1167	1433.34	307.01	386.21	513.96	1.53102	UP	1.07E-02
BPSS0577	BPSS0577	62.13	69.44	67.75	21.94	23.51	23.59	1.529582	UP	4.43E-05
BPSL0315	BPSL0315	39.14	48.25	54.47	12.94	14.55	21.72	1.527444	UP	4.05E-03
BPSL0096	speG	537.48	617.15	510.15	147.01	201.89	229.32	1.525641	UP	8.40E-04
BPSL2156	frr	399.44	414.33	380.71	117.78	144.19	154.56	1.51989	UP	5.98E-05
BPSL1920	rimP	212.27	261.04	274.14	71.96	81.08	107.86	1.51848	UP	1.71E-03
BPSL2562	rpoZ	1515.11	1747.39	1628.39	445.42	615.1	649.24	1.516303	UP	3.24E-04
BPSL1030	BPSL1030	22.71	26.51	33.73	9.07	8.95	11.05	1.512711	UP	5.55E-03
BPSL1744	BPSL1744	87.72	77.16	56.56	36.39	14.28	26.94	1.512601	UP	1.27E-02
BPSL2042	BPSL2042	26.12	33.03	35.41	8.2	11.89	13.19	1.506575	UP	2.96E-03
BPSL1351	carB	176.46	169.76	175.58	56.4	64.55	62.7	1.506538	UP	4.08E-06
BPSS2350	BPSS2350	45.45	54.92	50.06	15.11	18.56	19.3	1.505845	UP	4.25E-04
BPSS1261	BPSS1261	68.36	76.13	79.53	22.3	27.52	29.14	1.504434	UP	2.43E-04
BPSS1765	BPSS1765	142.42	166.73	167.73	44.47	54.37	69.4	1.503106	UP	7.28E-04
BPSS0562	BPSS0562	7.29	8.75	7.59	2.37	2.87	3.11	1.500772	UP	5.06E-04
BPSL2043	BPSL2043	41.53	41.49	38.49	12.68	14.14	16.18	1.498666	UP	5.24E-05
BPSL3231	paaB	416.26	376.78	363.02	108.42	151.99	150.11	1.493692	UP	3.13E-04
BPSL1035	BPSL1035	10.12	13.27	10.44	3.89	2.73	5.41	1.491667	UP	4.57E-03
BPSL1250	serA	62.86	63.96	61.09	22.6	22.68	21.55	1.491474	UP	1.56E-06
BPSL1514	hisS	224.68	228.39	239.22	74.02	88.61	84.03	1.488853	UP	1.72E-05

BPSS1778	BPSS1778	223.88	241.56	220.16	74.06	80.22	90.46	1.486117	UP	5.57E-05
BPSL1164	purD	177.14	176.74	169.58	59.08	55.18	73.41	1.479881	UP	5.06E-05
BPSL3198	rplF	672.09	700.74	634.4	200.91	251.86	267.81	1.477975	UP	1.04E-04
BPSL0792	BPSL0792	34.56	45.73	40.77	10.56	16.16	16.74	1.477962	UP	2.40E-03
BPSL2444	rpmF	2245.77	2489.55	2948.06	864.22	790.05	1109.39	1.475161	UP	1.96E-03
BPSL1513	ispG	192.41	214.75	240.6	66.26	75.48	91.89	1.471234	UP	9.49E-04
BPSL3230	paaA	236.25	234.02	190.82	68.4	88	82.09	1.470918	UP	8.93E-04
BPSL2952	gap	1216.81	1290.65	1305.24	424.29	443.25	507.92	1.470899	UP	2.62E-05
BPSL0394	BPSL0394	370.47	434.17	415.03	116.13	130.7	193.49	1.469867	UP	1.02E-03
BPSL1980	BPSL1980	189.48	194.69	196.37	58.59	67.66	83.34	1.469826	UP	8.01E-05
BPSL1212	BPSL1212	294.29	310.99	291	101.96	106.99	115.24	1.46711	UP	1.28E-05
BPSS2288	BPSS2288	1062.07	957.77	930.77	298.7	168.39	600.62	1.466493	UP	9.45E-03
BPSL0927	BPSL0927	249.57	259.35	241.3	79.76	84.06	107.73	1.466096	UP	9.53E-05
BPSL1211	BPSL1211	258.02	273.5	266.54	74.93	102.65	111.31	1.465977	UP	1.38E-04
BPSL2127	guaA	89.23	91.93	86.25	25.31	31.23	40.28	1.465676	UP	2.57E-04
BPSL1246	BPSL1246	125.52	133.27	114.89	41.05	49.03	45.32	1.464576	UP	1.65E-04
BPSL2261	BPSL2261	72.99	80.8	87.24	26.36	27.96	33.02	1.464498	UP	3.65E-04
BPSL1571	BPSL1571	67.66	67.96	81.96	27.47	25.16	26.44	1.460344	UP	6.35E-04
BPSS1577	bcsA	15.03	13.48	16.25	4.97	6.24	5.08	1.458223	UP	4.55E-04
BPSL1770	BPSL1770	70.59	77.19	80.11	26.07	27.6	29.28	1.458024	UP	8.30E-05
BPSS1594	pilM	59.7	57.38	58.86	23.31	20.46	20.34	1.456462	UP	6.07E-06
BPSS0488	BPSS0488	73.85	85.66	92.23	23.94	25.72	42.44	1.450661	UP	2.62E-03
BPSL1032	hisQ	37.02	33.42	37.66	11.79	12.86	14.93	1.449523	UP	1.43E-04
BPSL2937	lptE	443.21	445.66	448.94	149.06	170.59	170.29	1.449196	UP	2.68E-06
BPSL2138	BPSL2138	106.59	114.36	105.14	40.12	37.71	41.63	1.448743	UP	2.37E-05
BPSL1278	BPSL1278	63.39	72.23	68.63	24.91	24.32	25.68	1.447106	UP	7.71E-05

BPSL2522	BPSL2522	7423.51	8334.82	8227.68	2661.43	3073.15	3066.38	1.44646	UP	9.13E-05
BPSL1478	thrC	224.74	217.32	238.12	79.08	75.07	95.74	1.444623	UP	8.22E-05
BPSL1484	clpB	254.93	238.45	252.11	72.72	83.13	118.4	1.442697	UP	4.38E-04
BPSL0790	BPSL0790	89.5	92.81	100.36	35.18	29.62	39.23	1.442119	UP	1.51E-04
BPSL3040	BPSL3040	127.22	139.94	102.44	39.35	45.59	51.26	1.440238	UP	2.52E-03
BPSL2739	hmgA	59.15	55.63	54.87	19.22	20.21	23.15	1.438788	UP	3.57E-05
BPSL2820	BPSL2820	77.22	90.96	89.62	29.86	32.13	33.59	1.431472	UP	2.77E-04
BPSL1940	pheT	115.23	115.62	125.25	40.74	46.44	44.97	1.430106	UP	3.55E-05
BPSS1772	BPSS1772	12.24	14.79	14.04	3.16	4.34	7.82	1.422669	UP	5.70E-03
BPSS0309	BPSS0309	9.05	10.06	8.6	3.79	2.91	3.64	1.422171	UP	3.44E-04
BPSS0759	codB	51.55	52.88	54.81	19.27	16.16	24.05	1.420726	UP	1.80E-04
BPSL2742	BPSL2742	55.58	47.03	48.61	14.99	15.32	26.32	1.417011	UP	2.29E-03
BPSL2287	iscA	31.05	40.87	33.33	11.41	12.12	16	1.4128	UP	2.65E-03
BPSS1915	BPSS1915	51.36	43.19	40.26	8.97	15.05	26.72	1.409732	UP	1.05E-02
BPSL3194	rplO	1445.06	1279.37	1223.55	448.97	589.71	447.28	1.409719	UP	5.49E-04
BPSL1350	leuE	71.53	64.98	58.32	22.94	24.14	26.36	1.407578	UP	5.08E-04
BPSL1322	BPSL1322	893.07	823.14	815.6	344.97	307.98	302.18	1.4064	UP	4.79E-05
BPSL0690	BPSL0690	290.17	246.11	235.34	77.14	109.67	104.39	1.40588	UP	1.21E-03
BPSL3004	rpmA	931.45	1232.83	1192.32	305.58	415.37	546.19	1.405425	UP	4.03E-03
BPSL1258	BPSL1258	21.48	16.98	21.09	6.5	8.15	7.9	1.400974	UP	1.28E-03
BPSL2300	aceF	90.62	98.4	97.03	35.45	37.5	35.43	1.400169	UP	1.87E-05
BPSL0887	BPSL0887	227.09	211.64	202.86	68.42	84.29	90.5	1.399449	UP	1.62E-04
BPSL0531	ptsN	135.57	148.02	122.12	48.92	43.07	62.38	1.394056	UP	8.81E-04
BPSS0833	BPSS0833	678.21	553.65	558.61	101.43	164.23	416.83	1.391459	UP	2.42E-02
BPSL0189	gatB	70.65	74.57	74.42	27.33	27.04	29.41	1.390463	UP	6.87E-06
BPSL0119	BPSL0119	14.21	16.06	13.8	4.84	4.69	7.29	1.389619	UP	1.14E-03

BPSL1220	BPSL1220	147.47	149.04	148.37	50.75	63.72	55.67	1.386694	UP	1.77E-05
BPSS0575	BPSS0575	261.11	316.24	274.53	104.1	108.14	113.87	1.385292	UP	4.81E-04
BPSL0733	BPSL0733	63.63	70.88	69.12	25.61	21.63	31.03	1.379419	UP	2.79E-04
BPSL2281	lysS	95.66	92.48	87.63	30.22	32.89	43.09	1.376682	UP	2.44E-04
BPSS1930	BPSS1930	18.66	16.8	18.46	3.97	5.16	11.65	1.375625	UP	1.09E-02
BPSL3104	tssE	256.49	270.56	304	118.76	105.5	96.31	1.374296	UP	3.94E-04
BPSS1994	BPSS1994	101.43	114.27	95.59	29.56	36.48	54.08	1.373783	UP	2.23E-03
BPSL2160	map	290.52	313.34	336.98	102.26	116.51	144.36	1.373463	UP	4.55E-04
BPSL0227	fliI	8.36	6.81	7.64	3.48	2.57	2.76	1.372453	UP	8.96E-04
BPSL1257	BPSL1257	28.11	24.52	26.51	8.65	10.63	11.37	1.36852	UP	2.54E-04
BPSL0277	BPSL0277	5.73	6	7.17	2.74	2.16	2.42	1.368471	UP	1.22E-03
BPSS1357	BPSS1357	129.8	130.53	129.07	44.66	45.38	60.89	1.367373	UP	1.16E-04
BPSL0832	BPSL0832	130.66	123.12	110.68	60.07	39.49	41.72	1.367203	UP	1.05E-03
BPSL0052	BPSL0052	6.81	9.56	6.93	4.09	2.5	2.45	1.365935	UP	1.05E-02
BPSL3379	cadR	97.94	104.04	104.99	28.14	39.24	51.75	1.365561	UP	9.47E-04
BPSLt45	BPSLt45	85.13	82.86	77.34	28.77	31.58	34.88	1.365236	UP	6.71E-05
BPSL1402	tig	603.52	662.6	644.86	208.55	221.08	312.17	1.365211	UP	4.62E-04
BPSS1593	pilV	23.19	26.25	28.7	9.94	9.49	10.92	1.364365	UP	6.42E-04
BPSS2003	BPSS2003	602.49	569.85	558.41	162.54	209.45	300.79	1.363191	UP	1.17E-03
BPSS1992	BPSS1992	180.35	190.44	196.76	89.41	67.57	63.84	1.361877	UP	2.41E-04
BPSL3276	BPSL3276	230.74	282.81	305.36	92.52	92.07	134.53	1.359606	UP	3.14E-03
BPSL2503	BPSL2503	81.86	90.77	98.01	28.24	30.54	46.69	1.359542	UP	1.79E-03
BPSL0547	BPSL0547	90.7	116.15	105.27	34.99	32.24	54.5	1.358416	UP	3.36E-03
BPSL2381	cyoD	17.19	13.63	13.29	3.38	5.02	8.87	1.352838	UP	1.20E-02
BPSL3138	hisC	26.41	29.53	28.96	11.92	11.18	10.46	1.339022	UP	8.22E-05
BPSL0274	flgF	15.74	14.51	14.31	6.96	5.06	5.62	1.336899	UP	2.38E-04

BPSS1717	BPSS1717	260.53	266.43	247.95	99.02	107.5	100.34	1.336448	UP	1.35E-05
BPSL2260	BPSL2260	185.43	204.67	193.22	64.68	73.8	92.72	1.335146	UP	2.98E-04
BPSL2355	BPSL2355	24.6	18.94	19.5	7.22	8.17	9.6	1.334917	UP	2.77E-03
BPSL0905	lspA	83.85	82	88.77	32.28	39.49	29.23	1.33399	UP	1.50E-04
BPSL0643	BPSL0643	105.48	117.99	119.49	41.2	45.53	49.52	1.331784	UP	1.67E-04
BPSL2943	fur	134.72	154.8	110.1	47.44	57.31	54.64	1.326068	UP	3.79E-03
BPSS1554	bprQ	55.3	65.66	70.33	20.55	23.75	32.06	1.324872	UP	2.41E-03
BPSL2563	gmk	429.49	510.88	495.34	144.36	155.91	274.59	1.320482	UP	4.09E-03
BPSL2199	BPSL2199	487.92	533	501.3	197.41	217.19	195.02	1.320195	UP	3.58E-05
BPSL2987	tpx	776.39	828.9	736.04	298.7	354.25	285.74	1.318608	UP	1.64E-04
BPSL0186	BPSL0186	414.91	461.98	431.98	146.11	160.05	218.75	1.31818	UP	5.65E-04
BPSL0075a	rpmH	487.81	555.37	500.42	161.48	219.84	237.9	1.317775	UP	5.76E-04
BPSS1995	BPSS1995	39.16	40.38	42.07	13.59	14.58	20.62	1.317604	UP	4.99E-04
BPSL0206	BPSL0206	149.35	165.48	148.5	50.49	66.9	68.59	1.316893	UP	3.19E-04
BPSL2857	phnG	8.51	8.66	10.39	4.51	4.7	1.87	1.314618	UP	7.39E-03
BPSS1237	BPSS1237	18.48	20.56	21.34	7.69	7.72	8.87	1.314302	UP	2.13E-04
BPSS1949	BPSS1949	87.73	77.35	73.44	13.93	18.76	63.26	1.313756	UP	4.32E-02
BPSL1497	trxA	1322.71	1352.04	1346.13	485.75	577.04	560.92	1.308217	UP	1.11E-05
BPSS0887	bpsR	39.62	48.95	55.81	20.1	18.64	19.58	1.307808	UP	3.68E-03
BPSL2839	aroG	47	51.54	50.21	17.33	18.93	23.84	1.307453	UP	2.41E-04
BPSL3334	BPSL3334	16.96	20.9	23.29	11.34	6.41	6.97	1.306674	UP	7.35E-03
BPSL1525	BPSL1525	284.01	283.79	298.88	102.36	120.93	127.81	1.303617	UP	4.62E-05
BPSL1403	clpP	704.14	732.14	661.7	259.3	270.5	320.26	1.303364	UP	1.15E-04
BPSS1439	BPSS1439	12.49	15.51	18.32	5.99	5.22	7.58	1.30167	UP	7.29E-03
BPSL0666	gmhB	86.28	77.29	92.49	33.64	30.09	40.38	1.298373	UP	6.92E-04
BPSL2147	lpxA	317.85	343.83	356.59	125.81	148.15	140.6	1.296467	UP	1.06E-04

BPSL2314	BPSL2314	11.05	11.98	12.1	2.84	3.32	8.15	1.29568	UP	1.59E-02
BPSL2254	BPSL2254	790.18	811.77	769.95	242.1	358.64	367.83	1.292115	UP	3.78E-04
BPSS1707	leuC	76.69	79.28	78.18	29.01	29.38	37.35	1.290239	UP	8.19E-05
BPSL1259	BPSL1259	23.74	19.32	24.18	7.78	8.54	11.23	1.287267	UP	2.11E-03
BPSL1218	nuoH	152.74	150.06	146.81	59.78	68.64	55.9	1.286462	UP	2.85E-05
BPSS1843	BPSS1843	55.89	55.89	64.66	23.45	25.83	23.29	1.281732	UP	3.37E-04
BPSL0381	dsbA	187.56	204.8	206.52	77.85	80.54	88.45	1.278691	UP	6.77E-05
BPSL0794	BPSL0794	49.41	57.3	58.73	18.86	19.45	29.89	1.278464	UP	2.15E-03
BPSL1254	BPSL1254	36.57	32.1	28.34	12.11	11.68	16.21	1.278133	UP	2.40E-03
BPSL2368	BPSL2368	11.29	8.76	9.14	3.82	3.52	4.73	1.274049	UP	2.77E-03
BPSL3044	paaI	83.18	71.11	81.44	30.43	30.45	36.65	1.273217	UP	4.30E-04
BPSS1719	sdhD	337.74	386.15	348.51	127.92	167.66	148.44	1.272147	UP	3.56E-04
BPSS0829	BPSS0829	17.33	17.14	16.57	7.74	7.41	5.99	1.271653	UP	6.89E-05
BPSL2285	hscA	47.09	44.82	47.91	18.36	20.67	18.89	1.271437	UP	1.92E-05
BPSL0825	BPSL0825	28.92	27.55	26.95	13.07	9.31	12.18	1.27129	UP	2.17E-04
BPSL2431	lepA	113.43	120.48	123.82	40.31	49.67	58.24	1.271131	UP	3.15E-04
BPSL1944	infC	769.14	890.68	750.64	272.72	330.32	396.55	1.2699	UP	1.15E-03
BPSL2521	gyrA	458.58	470.88	461.94	166.45	188.42	222.19	1.269744	UP	8.23E-05
BPSL1216	nuoF	160.51	159.98	157.13	63.75	74.46	59.93	1.269343	UP	3.14E-05
BPSL0494	BPSL0494	112.44	98.37	94.04	38.14	37.67	50.67	1.26919	UP	1.05E-03
BPSL1511	rlmN	527.86	605.42	683.48	199.99	215.06	338.86	1.268904	UP	4.88E-03
BPSL0799	BPSL0799	55.28	60.85	59.17	22.2	24.74	25.9	1.267023	UP	6.61E-05
BPSL1230	BPSL1230	43.54	52.17	55.85	16.46	21.63	24.94	1.265778	UP	2.58E-03
BPSL2430	lepB	151.61	170.69	162.03	56.32	68.35	76.77	1.26564	UP	3.11E-04
BPSL1098	BPSL1098	158.65	166.53	168.51	57.02	62.26	86.21	1.264537	UP	5.33E-04
BPSL1846	BPSL1846	44.21	42.23	42.78	18.55	14.88	20.37	1.264151	UP	1.27E-04

BPSL0121	def	235.72	263.47	314.17	71.96	102.55	164.39	1.263034	UP	1.13E-02
BPSL3399	BPSL3399	574.07	648.91	584.81	264.14	283.36	205.76	1.263007	UP	4.41E-04
BPSS1405	sctS	6.67	8.8	4.56	1.7	3.45	3.2	1.262314	UP	4.39E-02
BPSL2316	ntrC	54.67	50.97	58.78	22.76	21	24.95	1.258794	UP	2.27E-04
BPSS0836	BPSS0836	419.8	343.06	432.37	83.71	137.4	279.23	1.256308	UP	2.31E-02
BPSL1515	BPSL1515	189.33	188.08	199.74	74.16	78.23	89.33	1.255609	UP	4.41E-05
BPSL2961	BPSL2961	66.82	68.18	80.78	31.11	26.25	33.05	1.255007	UP	1.02E-03
BPSS0133	BPSS0133	21.26	21.76	22.22	11.47	7.16	8.75	1.252634	UP	6.09E-04
BPSS0497	BPSS0497	5.57	8.18	8.19	3.21	2.9	3.13	1.247599	UP	8.47E-03
BPSS2319	BPSS2319	7.15	9.55	8.73	3.64	2.87	4.2	1.247573	UP	3.63E-03
BPSL2934	BPSL2934	37.32	35.65	42.86	13.38	12.47	22.94	1.247352	UP	5.01E-03
BPSL0117	BPSL0117	580.96	659.88	671.56	243.47	233.14	332.78	1.240477	UP	9.86E-04
BPSL1215	nuoE	255.39	237.74	245.66	91.04	113.9	108.24	1.238172	UP	7.77E-05
BPSL1468	BPSL1468	152.42	155.23	161.96	64.41	61.79	73.02	1.237101	UP	3.41E-05
BPSL1467	BPSL1467	155.81	170.18	145.84	65.28	56.29	78.65	1.236681	UP	7.04E-04
BPSL3043	paaG	332.69	280.75	291.13	99.55	125.87	159.24	1.233648	UP	1.79E-03
BPSL3062	ychF	235.24	272.65	258.81	85.39	93.3	147.75	1.231844	UP	2.82E-03
BPSL0073	gyrB	164.9	175.13	183.09	69.07	64.41	89.37	1.231069	UP	4.22E-04
BPSL1530	BPSL1530	127.73	126.65	138.37	50.9	50.97	65.46	1.230915	UP	2.52E-04
BPSL2758	BPSL2758	400.97	443.64	392.17	157	171.6	198.64	1.230057	UP	2.95E-04
BPSL3289	BPSL3289	448.23	410.38	354.37	163.49	182.88	170.73	1.230041	UP	1.13E-03
BPSS1771	BPSS1771	365.63	393.27	335.38	156.01	140.88	169.77	1.229538	UP	3.62E-04
BPSL2896	purH	165.03	153.8	165.78	61.64	62.92	82.11	1.229495	UP	2.70E-04
BPSS0846	BPSS0846	45.17	48.85	47.33	14.47	19.12	26.71	1.229042	UP	1.92E-03
BPSL0519	pth	33.54	34.3	33.42	14.7	16.87	11.63	1.228961	UP	2.34E-04
BPSL3397	atpG	578.24	588.44	519.71	216.3	287.77	215.99	1.227749	UP	5.53E-04

BPSL2357	BPSL2357	17.97	13.57	17.96	5.58	5.12	10.47	1.225407	UP	1.38E-02
BPSL1790	BPSL1790	6.7	6.09	7.01	3.06	2.33	3.09	1.223364	UP	5.05E-04
BPSL2445	BPSL2445	182.71	220.87	239.8	70.5	92.39	112.8	1.222624	UP	4.12E-03
BPSL1524	BPSL1524	499.83	526.85	531.4	197.98	227.57	243.62	1.219325	UP	5.80E-05
BPSL0491	BPSL0491	239.72	215.9	160.11	82.52	103.27	78.89	1.218049	UP	9.16E-03
BPSL0475	BPSL0475	56.02	47.5	41.31	16.75	25.12	20.39	1.217983	UP	4.95E-03
BPSS1131	BPSS1131	47.5	53.54	48.7	19.72	19.3	25.37	1.217551	UP	4.53E-04
BPSL1116	BPSL1116	61.43	68.26	68.3	28.69	27.64	28.93	1.215487	UP	8.46E-05
BPSS1720	sdhC	759.32	836.89	744.4	256.92	384.81	366.45	1.215131	UP	8.31E-04
BPSL1939	BPSL1939	136.5	112.52	120.74	51.13	56.94	51.48	1.212581	UP	6.52E-04
BPSL2272	BPSL2272	527.01	572	583.94	213.02	229.26	284.24	1.211918	UP	3.23E-04
BPSL0881	BPSL0881	28.55	30.83	30.33	12.86	12.82	13.06	1.211445	UP	1.67E-05
BPSS1596	BPSS1596	42.21	37.22	42.24	18.57	16.92	17.06	1.211211	UP	1.92E-04
BPSS1633	BPSS1633	7.98	8.2	8.15	3.06	3.46	4	1.209602	UP	8.06E-05
BPSL0532	raiA	740.84	811.35	709.23	227.4	302.18	448.41	1.209337	UP	3.94E-03
BPSS1724	BPSS1724	327.2	359.1	343.66	170.35	162.88	112.48	1.208411	UP	6.69E-04
BPSL1760	BPSL1760	55.75	49.4	45.29	25.85	20.76	18.55	1.20713	UP	1.59E-03
BPSS1114	ftsH	226.57	188.73	198.18	49.6	53.33	163.36	1.204018	UP	4.13E-02
BPSL1866	BPSL1866	51.16	60.49	58.08	21.28	23.51	28.99	1.20194	UP	9.01E-04
BPSL0625	BPSL0625	26.53	28.45	32.1	12.13	10.14	15.59	1.201667	UP	1.98E-03
BPSL2642	trmB	21.91	26.59	23.07	8.86	9.35	12.91	1.201513	UP	2.08E-03
BPSS1585	BPSS1585	953.31	698.48	802.88	157.29	298.6	612.01	1.200753	UP	3.94E-02
BPSS1878	BPSS1878	20.91	21.46	19.56	8.93	7.81	10.21	1.200353	UP	2.00E-04
BPSS1634	BPSS1634	5.74	5.93	5.61	2.4	2.56	2.56	1.200299	UP	7.01E-06
BPSL3073	prfA	65.81	75.74	80.92	22.92	30.29	43.68	1.199191	UP	5.11E-03
BPSL2408	BPSL2408	225.36	238.25	223.05	98.54	106.28	94.32	1.198775	UP	2.55E-05

BPSL3214	rpsJ	1790.42	2272.4	2025.14	645.24	1017.92	991.14	1.197627	UP	3.38E-03
BPSS1270	BPSS1270	6.57	7.77	5.24	2.32	3.07	3.15	1.197073	UP	9.06E-03
BPSS1211	BPSS1211	37.12	45.26	45.01	17.17	18.86	19.54	1.196874	UP	9.78E-04
BPSL2040	BPSL2040	22.35	25.49	23.12	10.75	8.97	11.28	1.194738	UP	3.45E-04
BPSL0990	BPSL0990	62.15	66.84	66.79	26.75	25.94	32.86	1.194394	UP	1.64E-04
BPSL0197	BPSL0197	50.57	52.59	55.58	22.84	22.63	24.08	1.190543	UP	4.07E-05
BPSS2043	BPSS2043	125.58	150.32	142.05	54.05	56.55	72.88	1.187708	UP	1.13E-03
BPSL2982	aroQ	173.49	203.93	201.19	72.47	79.08	102.56	1.187138	UP	1.26E-03
BPSS0857	BPSS0857	14.24	16.98	19.58	7.91	6.06	8.35	1.186491	UP	4.98E-03
BPSL3290	ahcY	985.76	988.49	807.52	359.21	423.4	441.37	1.184423	UP	1.32E-03
BPSL0918	BPSL0918	364.61	382.48	397.71	159.5	158.57	186.85	1.180969	UP	8.90E-05
BPSL1867	BPSL1867	137.16	142.41	142.35	60.06	59.2	66.85	1.180814	UP	1.23E-05
BPSL0898	BPSL0898	368.65	484.25	392.26	125.78	211.63	212.96	1.177857	UP	7.06E-03
BPSL1765	BPSL1765	160.16	156.38	176.67	77.79	64.75	75.78	1.175758	UP	2.49E-04
BPSS2347	BPSS2347	47.91	50.08	50.83	17.03	22.21	26.65	1.175437	UP	6.89E-04
BPSL2872	secF	229.83	218.13	223.73	95.62	101.04	100.81	1.175051	UP	5.21E-06
BPSL1919	nusA	509.12	542.05	567.81	183.95	208.96	324.14	1.17494	UP	2.92E-03
BPSS0130	BPSS0130	11.03	11.52	10.98	6.2	4.15	4.51	1.174018	UP	6.85E-04
BPSL3142	BPSL3142	87.23	98.31	106.67	35.63	42.74	51.22	1.173051	UP	1.68E-03
BPSL2928	purB	213.93	224.75	214.51	82.94	95.42	111.4	1.172644	UP	1.73E-04
BPSS0296	BPSS0296	144.08	142.43	133.04	60.6	57.59	68.01	1.17199	UP	7.34E-05
BPSL1472	BPSL1472	108.6	113.7	106.71	45.35	49.79	50.94	1.171373	UP	2.26E-05
BPSL2289	BPSL2289	47.72	50.99	50.1	18.26	22.4	25.45	1.170531	UP	2.78E-04
BPSL0875	adk	401.19	490.17	446.25	164.74	173.79	256.56	1.168478	UP	3.13E-03
BPSL1082	BPSL1082	433.23	460.24	476.42	226.55	220.92	162	1.168433	UP	4.68E-04
BPSL0231	fliE	33.09	27.27	23.81	13.63	11.48	12.34	1.16834	UP	4.98E-03

BPSS1706	leuD	64.21	68.71	76.58	23.9	29.72	39.71	1.166537	UP	2.72E-03
BPSL0481	BPSL0481	66.02	53.24	48	24.43	28.34	21.8	1.165425	UP	5.54E-03
BPSL1933	bamC	388.53	448.31	484.46	157.06	190.72	241.58	1.164737	UP	2.80E-03
BPSL0077	yidD	76.41	79.58	73.44	30.01	42.7	29.9	1.160883	UP	7.81E-04
BPSL0793	BPSL0793	223.2	233.56	246.86	105.68	83.84	125.6	1.158895	UP	7.32E-04
BPSL0406	BPSL0406	67.97	57.97	70.59	28.31	29.62	30.13	1.158191	UP	7.40E-04
BPSL1523	BPSL1523	152.16	164.43	166.78	64.34	70.49	81.76	1.158161	UP	2.00E-04
BPSL2315	xth	29.87	38.89	32.99	13.12	14.98	17.53	1.156974	UP	3.12E-03
BPSL2313	BPSL2313	22.9	19.61	21.41	5.92	8.34	14.42	1.156222	UP	1.22E-02
BPSL2301	aceE	185.1	200.27	198.69	95.65	92.85	73.84	1.154679	UP	2.14E-04
BPSL1500	recR	438.2	446.34	435.34	182.95	185.6	224.54	1.154084	UP	6.26E-05
BPSS1579	bcsE	14.22	12.33	13.23	5.65	6.76	5.47	1.153697	UP	4.24E-04
BPSL0182	rodA	41.07	39.28	39.56	18.02	19.12	16.78	1.15306	UP	1.49E-05
BPSL1188	BPSL1188	387.31	440.89	456.95	152.24	173.82	252.07	1.152471	UP	3.09E-03
BPSS0943	BPSS0943	1131.89	1211.36	1164.67	507.67	519.3	551.75	1.151861	UP	1.73E-05
BPSL0644	aspS	252.69	245.72	255.73	110.05	113.15	116.67	1.149849	UP	2.54E-06
BPSS0050	BPSS0050	18.96	19.72	14.67	6.04	8.06	9.95	1.149451	UP	7.25E-03
BPSL3137	hisB	114.02	126.22	120.32	52.5	46.82	63.23	1.149356	UP	3.79E-04
BPSL2298	BPSL2298	4937.24	5827.46	5056.67	2330.64	2677.53	2147.88	1.144639	UP	8.27E-04
BPSL3232	paaC	282.43	236.12	236.05	94.9	118.29	128.16	1.14446	UP	1.68E-03
BPSL2151	bamA	665.82	723.09	769.82	291.81	334.69	350.53	1.143708	UP	3.48E-04
BPSL2491	rimM	1090.65	1157.3	1253.93	490	493.48	604.31	1.14111	UP	4.56E-04
BPSL2870	yajC	301.67	370.29	322.02	130.98	138.37	182.61	1.137022	UP	2.24E-03
BPSS2287	BPSS2287	37.14	28.81	28.15	6.44	13.96	22.39	1.136921	UP	3.47E-02
BPSS0290	ceoR	11.93	11.67	13.42	5.5	5.35	5.99	1.136413	UP	3.12E-04
BPSL0185	mreC	30.21	30.15	34.25	12.72	13.72	16.72	1.132298	UP	6.96E-04

BPSL1070	parC	44.32	45.71	51.21	23.9	21.96	18.63	1.131001	UP	6.04E-04
BPSL2443	plsX	81.37	100.1	109.87	35.72	39	58.41	1.129868	UP	8.57E-03
BPSL0238	BPSL0238	52.98	58.52	55.56	22.1	24.79	29.57	1.127589	UP	3.68E-04
BPSL1003	BPSL1003	71.01	73.83	71.01	31.75	31.93	35.36	1.123946	UP	1.32E-05
BPSL3295	flhB	6.29	5.13	6.08	3.17	2.26	2.6	1.123883	UP	2.08E-03
BPSL3226	nusG	332.32	366.52	327.17	131.84	155.07	184.55	1.121838	UP	7.08E-04
BPSL2157	pyrH	354.7	367.94	318.81	152.08	164.18	163.13	1.119322	UP	2.48E-04
BPSL1099	BPSL1099	177.19	216.3	200.54	79.4	87.29	106.78	1.119153	UP	1.57E-03
BPSS0755	BPSS0755	7.87	7.38	9.11	3.21	3.49	4.53	1.117156	UP	2.58E-03
BPSL0973	BPSL0973	113.8	130.63	123.69	53.17	52.9	63.65	1.11702	UP	3.93E-04
BPSS1718	sdhA	294.72	314.23	302.76	132.22	142.9	145.32	1.116675	UP	1.92E-05
BPSS0605	BPSS0605	9.07	14.18	17.41	5.14	5.47	8.15	1.11595	UP	4.89E-02
BPSL2271	kdsA	302.98	289.21	293.32	130.76	133.19	144.82	1.115219	UP	1.17E-05
BPSS1631	BPSS1631	21.3	21.79	25.09	9.33	10.33	11.87	1.112623	UP	9.52E-04
BPSS1777	BPSS1777	118.46	109.88	118.56	52.94	51.97	55.52	1.112576	UP	3.49E-05
BPSL2382	BPSL2382	5.44	3.93	3.62	1.15	1.75	3.11	1.111965	UP	4.50E-02
BPSL2042A	BPSL2042A	22.59	23.36	28.41	11.51	10.78	12.15	1.110442	UP	2.05E-03
BPSL2938	leuS	184.23	188.02	192.43	75.27	76.9	109.74	1.108362	UP	9.22E-04
BPSL1418	BPSL1418	170.94	186.14	172.63	75.44	76.01	94.48	1.106955	UP	2.77E-04
BPSL3416	BPSL3416	32.57	51.32	46.14	18.45	20.02	21.9	1.106941	UP	1.50E-02
BPSS1879	BPSS1879	26.07	27.62	27.43	12.62	9.67	15.46	1.103581	UP	1.15E-03
BPSS1753	BPSS1753	147.73	168.86	128.78	57.49	79.53	70.52	1.101615	UP	3.90E-03
BPSL0814	bpeA	86.31	105.48	99.97	49.29	43.37	43.56	1.098844	UP	9.99E-04
BPSL1372	BPSL1372	31.28	26.09	24.02	11.46	13.51	13.16	1.093925	UP	3.05E-03
BPSL2561	BPSL2561	258.39	284.74	311.81	107.6	111.6	181.55	1.093121	UP	6.07E-03
BPSL1214	BPSL1214	234.97	224.43	240.6	110.25	113.29	105.18	1.090496	UP	1.99E-05

BPSL1745	arcC	70.92	62.72	58.49	40.24	14.44	35.63	1.089125	UP	1.78E-02
BPSL0906	ileS	72.1	68.94	73	33.5	37.63	29.53	1.08839	UP	1.39E-04
BPSL0228	fliH	10	7.23	6.08	3.92	3.95	3.1	1.087385	UP	2.63E-02
BPSS0631	BPSS0631	16.12	16.2	19.26	6.16	7.17	10.96	1.086449	UP	7.07E-03
BPSL2985	prmA	130.63	134.48	142.84	63.56	63.92	64.66	1.086234	UP	3.78E-05
BPSL0074	dnaN	675.52	690.65	623.64	269.81	313.76	353.9	1.085786	UP	3.77E-04
BPSS1569	bpsR3	1457.15	1564.6	1736.09	728.17	684.27	832.2	1.083823	UP	8.17E-04
BPSS0124	BPSS0124	8.12	10.37	8.79	4.04	4.41	4.43	1.082711	UP	2.11E-03
BPSL2874	BPSL2874	617.78	660.43	598.87	323.93	316.09	246.75	1.081858	UP	4.16E-04
BPSS0767	BPSS0767	235.98	292.47	233.32	115.22	130.44	115.12	1.078236	UP	2.58E-03
BPSL1290	thiC	33.49	38.43	32.38	16.89	17.15	15.38	1.077572	UP	7.05E-04
BPSL0011	gspG	213.88	246.53	268.49	107.53	109.27	128.76	1.076785	UP	1.78E-03
BPSL1844	nodI	24.07	28.15	25.58	12.48	11.41	13.04	1.074977	UP	4.46E-04
BPSS0806	BPSS0806	48.21	50.19	41.39	17.8	22.69	25.88	1.074658	UP	2.33E-03
BPSL0545	uvrA	98.45	96.88	97.3	42.22	42.56	54.23	1.073889	UP	2.10E-04
BPSL1531	BPSL1531	369.3	431.53	385.62	168.52	191.51	204.07	1.072628	UP	6.27E-04
BPSL1002	dcd	131.46	148.18	164.74	58.16	69.23	83.94	1.072296	UP	3.09E-03
BPSL2288	iscU	93.9	109.83	122.8	49.18	53.93	52.28	1.071322	UP	2.53E-03
BPSL1165	BPSL1165	256.6	309.13	319.83	104.58	120.04	196.81	1.071297	UP	1.10E-02
BPSS0350	BPSS0350	12.15	11.07	11.29	5.07	4.81	6.55	1.070682	UP	6.84E-04
BPSL1985	BPSL1985	253.08	283.27	294.01	119.49	127.07	148.98	1.069913	UP	6.60E-04
BPSS1456	BPSS1456	7.6	8.5	5.31	3.25	3.33	3.62	1.069716	UP	1.74E-02
BPSL2953	tkt	471.14	484.38	461.24	193.2	209.74	272.33	1.069059	UP	5.89E-04
BPSL2009	alaS	283.77	293.63	316.76	117.03	122.72	186.62	1.068427	UP	3.06E-03
BPSS0890	BPSS0890	44.13	46.43	36.86	25.61	17.97	17.2	1.067923	UP	4.87E-03
BPSL2765	pal	830.71	945.97	854.33	420	424.96	410.29	1.067642	UP	2.05E-04

BPSLt46	BPSLt46	112.47	152.8	120.39	56.16	47.04	81.15	1.064882	UP	1.38E-02
BPSL1858	ssuD	7.28	6.84	7.71	3.7	3.2	3.54	1.06419	UP	2.00E-04
BPSL1964	BPSL1964	372.2	379.84	340.14	139.75	153.31	229.75	1.062852	UP	3.41E-03
BPSL2247	BPSL2247	346.03	321.26	303.02	156.63	177.27	130.59	1.062798	UP	7.84E-04
BPSL1105	bspR	153.81	161.14	165.23	69.03	67.86	93.1	1.062004	UP	7.16E-04
BPSL2514	BPSL2514	1422.57	1268.13	1368.43	536.29	604.72	803.23	1.061964	UP	1.56E-03
BPSS0201	pip	55.09	54.92	48.98	28.25	23.9	24.07	1.060695	UP	3.61E-04
BPSL2473	BPSL2473	197.26	197.31	192.44	77.9	96.8	106.87	1.059891	UP	2.97E-04
BPSS1205	BPSS1205	7.71	9.08	10	4.04	3.9	4.92	1.058804	UP	3.26E-03
BPSL1946	BPSL1946	96.23	95.88	93.28	43.63	43.99	49.65	1.055918	UP	2.17E-05
BPSL0188	gatA	103.08	105.25	105.8	51.13	47.57	52.62	1.053759	UP	5.91E-06
BPSL0179	queD	37.24	44.51	51.27	17.81	16.78	29.53	1.052797	UP	1.63E-02
BPSL1126	BPSL1126	24.64	27.58	33.38	14.29	13.66	13.39	1.050072	UP	4.64E-03
BPSL0478	BPSL0478	22.15	22.37	20.4	9.57	10.09	11.7	1.049737	UP	2.35E-04
BPSL2045	BPSL2045	34.91	39.99	41.59	16.34	19.37	20.57	1.049512	UP	1.07E-03
BPSS0560	betC	13.6	14.25	14.86	6.93	5.61	8.11	1.048432	UP	8.11E-04
BPSL1841	BPSL1841	107.64	109.1	90.31	54.96	45.16	48.46	1.047234	UP	1.39E-03
BPSL2734	BPSL2734	626.55	606.53	606.16	307.17	272.62	310.82	1.046244	UP	2.22E-05
BPSL2752	BPSL2752	6.81	9.01	10.63	3.2	3.77	5.85	1.044871	UP	2.94E-02
BPSL2286	hscB	45.55	54.42	54.05	22.35	24.87	27.5	1.043551	UP	1.25E-03
BPSL0341	BPSL0341	85.18	77.52	75.91	40.05	36.07	39.68	1.043019	UP	1.97E-04
BPSS1275	BPSS1275	5.35	4.58	5.33	2.82	2.29	2.3	1.04221	UP	1.05E-03
BPSL2996	BPSL2996	95.49	105.37	106.67	42.99	37.82	68.55	1.041933	UP	6.51E-03
BPSL1769	cobW	87.31	86.54	86.74	47.12	46.53	32.93	1.041732	UP	6.51E-04
BPSS1852	BPSS1852	17.96	17.91	16.05	9.51	6.94	8.77	1.041722	UP	8.45E-04
BPSS1578	bcsQ	21.05	19.87	21.64	9.08	11.2	10.11	1.041644	UP	1.82E-04

BPSL0439	BPSL0439	51.44	57.26	67.01	25.01	22.49	37.94	1.040213	UP	1.03E-02
BPSL2875	BPSL2875	85.93	102.04	95.24	48.15	42.33	47.26	1.039925	UP	6.37E-04
BPSS1709	BPSS1709	20.94	21.18	21.93	9.26	9.48	12.46	1.037653	UP	5.22E-04
BPSL1799	BPSL1799	11.61	10.92	11.38	6.26	6.11	4.18	1.03488	UP	1.17E-03
BPSL2603	lolA	184.26	193.49	170.52	81.13	90.24	96.56	1.03303	UP	3.13E-04
BPSS1410	BPSS1410	6.31	4.33	3.57	1.97	2.47	2.51	1.031822	UP	4.42E-02
BPSL3292	BPSL3292	10.92	10.31	11.54	5.97	5.46	4.6	1.031601	UP	4.76E-04
BPSL1509	BPSL1509	1360.85	1379.42	1186.58	561.93	711.23	648.6	1.030944	UP	8.83E-04
BPSS1571	BPSS1571	61.29	63.51	70.44	29.29	23.45	42.85	1.030317	UP	6.46E-03
BPSL2796	BPSL2796	130.36	130.78	117.14	56.93	66.07	62.4	1.028813	UP	2.47E-04
BPSS1107	BPSS1107	344.48	329.07	331.71	194.9	143.97	154.34	1.027295	UP	4.65E-04
BPSS1679	BPSS1679	4379.83	4560.87	4291.19	2231.58	2284.63	1976.31	1.027169	UP	5.44E-05
BPSL2012	BPSL2012	254.5	253.49	250.17	98.45	132.36	141.27	1.026889	UP	6.04E-04
BPSL0438	BPSL0438	349.12	387	394.48	155.55	162.44	237.47	1.025334	UP	2.99E-03
BPSS1430	BPSS1430	14.21	22.77	23.18	10.53	8.45	10.58	1.025158	UP	2.75E-02
BPSL2617	BPSL2617	16.28	16.53	13.66	7.53	7.17	8.14	1.024737	UP	1.20E-03
BPSS0373	BPSS0373	16.24	23.43	24.94	9.92	10.27	11.57	1.024547	UP	1.60E-02
BPSL2266	BPSL2266	301.46	331.85	334.45	124.76	152.96	198.44	1.023203	UP	2.38E-03
BPSL2713	BPSL2713	14.48	13.88	14.14	7.49	6.12	7.31	1.02258	UP	1.01E-04
BPSL2740	BPSL2740	29.88	24.12	25.27	11.4	11.8	15.82	1.022561	UP	4.01E-03
BPSL0812	bpeR	70.26	82.65	83.68	32.71	38.95	44.81	1.022431	UP	1.96E-03
BPSL2995	ffh	208.53	211.85	188.5	83.42	95.61	120.78	1.022109	UP	1.45E-03
BPSL1633	BPSL1633	40.29	46.51	54.23	21.82	18.68	28.95	1.021955	UP	9.13E-03
BPSL2415	BPSL2415	50.68	53.31	61.19	25.46	22.66	33.24	1.021647	UP	3.34E-03
BPSL3106	tssC	174.19	176.96	189.29	107.52	97.41	61.31	1.021407	UP	3.47E-03
BPSS0630	BPSS0630	82.24	86.14	83.11	40.47	41.49	41.95	1.021208	UP	4.60E-06

BPSL0673	BPSL0673	147.49	139.15	142.03	59.48	62.66	89.11	1.020916	UP	1.72E-03
BPSL1835	BPSL1835	9.76	9.53	9.28	4.58	4.9	4.61	1.019829	UP	9.62E-06
BPSL3013	BPSL3013	71.36	74.54	84.27	33.01	34.35	46.2	1.019245	UP	2.44E-03
BPSS0959	BPSS0959	6.6	9.87	10.36	4.45	5.11	3.69	1.017855	UP	2.23E-02
BPSL2039	BPSL2039	15.18	18.56	15.38	7.74	8.7	7.82	1.017731	UP	1.89E-03
BPSL1981	galU	198.84	200.59	202.81	98.03	89.73	109.72	1.017546	UP	6.72E-05
BPSL2519	serC	211.82	221.76	236.87	88.56	100.58	142.1	1.017253	UP	3.13E-03
BPSL1725	BPSL1725	374.05	420.72	319.95	168.18	160.89	221.83	1.016819	UP	5.74E-03
BPSL3242	BPSL3242	92.12	90.8	93.83	48.71	39.43	48.71	1.015988	UP	1.31E-04
BPSL2827	dnaK	259.52	259.8	262.72	106.19	116.17	165.96	1.009996	UP	2.09E-03
BPSL1100	BPSL1100	136.33	168.69	161.97	73.02	79.54	79.36	1.009764	UP	1.48E-03
BPSL1166	upp	98.24	88.68	105.73	50.53	41.4	53.5	1.008851	UP	1.32E-03
BPSL0078	yidC	135.61	148.85	147.41	55.43	68.56	90.72	1.008208	UP	2.87E-03
BPSS0543	BPSS0543	13.23	13.01	12.66	5.78	6.42	7.14	1.008182	UP	1.07E-04
BPSS1699	trpB	186.35	184.23	191.24	85.39	94.22	99.72	1.008137	UP	3.55E-05
BPSS0317	BPSS0317	20.91	19.28	17.34	12.03	7.02	9.58	1.006787	UP	5.61E-03
BPSS0363	BPSS0363	105.46	104.11	102.72	40.09	51.86	63.6	1.005508	UP	1.57E-03
BPSL1798	BPSL1798	15.72	14.04	17.29	8.49	8.55	6.4	1.005222	UP	2.58E-03
BPSS1356	BPSS1356	3837.18	4028.5	3570.61	1812.91	2122.86	1767.59	1.003735	UP	3.86E-04
BPSS1913	BPSS1913	51.37	53.38	56.84	27.07	27.07	26.48	1.003128	UP	7.41E-05
BPSL2024	BPSL2024	19.1	19.56	21.14	10.16	8.21	11.52	1.000483	UP	9.49E-04
BPSS1221	BPSS1221	15.93	15.79	15.23	30.28	32.24	31.45	-1.00108	DOWN	1.35E-05
BPSS2311	BPSS2311	9.95	9.42	9.72	22.74	16.42	19.11	-1.00223	DOWN	6.12E-03
BPSL0316	BPSL0316	23.96	22.38	24.85	40.19	45.11	57.62	-1.00546	DOWN	1.03E-02
BPSL0101	BPSL0101	22.44	23.27	25.75	53.38	44.36	45.86	-1.00685	DOWN	1.25E-03
BPSS1167	BPSS1167	16.39	16.15	13.56	27.76	31.89	33.16	-1.00951	DOWN	1.13E-03

BPSL3357	BPSL3357	19.68	19.23	20.81	47.16	40.13	33.04	-1.01071	DOWN	7.91E-03
BPSS1603	BPSS1603	11.27	10.09	11.59	26.12	21.02	19.39	-1.01373	DOWN	5.74E-03
BPSS0065	BPSS0065	8.1	8.68	10.15	22.8	16.29	15.31	-1.01439	DOWN	1.96E-02
BPSL1286	BPSL1286	7.31	6.19	7.64	15.6	13.4	13.75	-1.01595	DOWN	8.89E-04
BPSL0596	BPSL0596	106.31	97.29	106.93	245	203.62	180.41	-1.0184	DOWN	5.17E-03
BPSS0714	egtD	46.63	44.16	53.75	100.25	82.7	110.01	-1.01923	DOWN	4.32E-03
BPSL0848	kynB	23.32	23.45	24.76	46.53	45.49	53.12	-1.02082	DOWN	5.44E-04
BPSS0945	BPSS0945	9.51	11.29	12.7	23.81	20.03	24.28	-1.02392	DOWN	2.11E-03
BPSL2532	BPSL2532	48.75	44.11	47.79	106.42	99.42	80.23	-1.02426	DOWN	3.67E-03
BPSS1468	BPSS1468	13.35	13.96	15.45	27.8	34.8	24.4	-1.02475	DOWN	9.17E-03
BPSS1639	BPSS1639	16.67	17.35	16.38	43.27	31.83	27.46	-1.02497	DOWN	2.12E-02
BPSL0687	glpK	7.87	8.63	8.85	19.32	11.79	20.48	-1.02511	DOWN	3.32E-02
BPSS1483	BPSS1483	60.06	61.36	59.84	121.94	118.25	128.84	-1.02568	DOWN	3.74E-05
BPSL1234	BPSL1234	63.47	64.7	74.68	158.36	121.18	133.87	-1.02716	DOWN	3.62E-03
BPSL1102	BPSL1102	9.55	10.25	9.49	21.41	16	22.45	-1.03118	DOWN	7.18E-03
BPSS1871	BPSS1871	14.49	12.65	15.17	32.52	26.98	27.1	-1.03337	DOWN	1.72E-03
BPSS1883	BPSS1883	7.15	7.62	9.04	20.37	15.47	12.94	-1.03472	DOWN	2.10E-02
BPSL1361	pstA	18.26	19.5	21.07	40.24	43.04	37.27	-1.03501	DOWN	3.75E-04
BPSL1826	BPSL1826	49.54	56.79	58.42	111.99	87.76	138.2	-1.03653	DOWN	1.76E-02
BPSL0716	BPSL0716	6.15	6.3	5.9	12.76	9.01	15.91	-1.03802	DOWN	3.21E-02
BPSS2157	BPSS2157	5.04	4.57	5.18	13.11	8.71	8.58	-1.03945	DOWN	2.56E-02
BPSS0183	BPSS0183	7.13	6.49	8.6	15.53	12.02	18.25	-1.04349	DOWN	1.46E-02
BPSL1802	BPSL1802	18.3	14.53	18.73	43.79	33.17	29.66	-1.04815	DOWN	1.46E-02
BPSL0854	pdxH	178.15	165.77	165.29	393.82	309.58	350.42	-1.0493	DOWN	1.82E-03
BPSL0100	ada	21.32	19.3	20.08	48.39	40.06	37.31	-1.0509	DOWN	3.04E-03
BPSL1569	BPSL1569	139.21	190.5	185.7	294.67	316.97	456.44	-1.05123	DOWN	2.57E-02

BPSS1874	BPSS1874	34.2	27.96	34.8	74.9	56.9	69.2	-1.05173	DOWN	3.79E-03
BPSL2527	BPSL2527	7.04	7.35	8.96	17.75	12.45	18.23	-1.05248	DOWN	1.27E-02
BPSL1756	BPSL1756	23.69	26.11	29.09	66.1	53.59	43.94	-1.05252	DOWN	1.29E-02
BPSL2704	BPSL2704	195	202.07	182.53	411.14	443.55	349.49	-1.05492	DOWN	1.79E-03
BPSS1882	BPSS1882	35.52	35.03	35.08	78.52	58.37	82.6	-1.05514	DOWN	7.15E-03
BPSS1243	nirB	38.8	40.02	36.7	94.13	85.53	60.75	-1.05735	DOWN	1.44E-02
BPSL2964	BPSL2964	11.7	12.13	13.32	31.14	26.31	19.97	-1.05934	DOWN	1.48E-02
BPSL1022	BPSL1022	10.01	9.86	7.87	18.83	16.45	22.76	-1.06508	DOWN	6.79E-03
BPSL0928	BPSL0928	17.94	15.66	15.93	34.98	31.29	37.38	-1.06535	DOWN	7.03E-04
BPSL2221	BPSL2221	70.23	62.3	62.74	134.24	121.43	154.25	-1.06987	DOWN	1.94E-03
BPSL1168	BPSL1168	79.9	71.25	76.04	180.98	145.39	150.72	-1.07036	DOWN	1.84E-03
BPSS2344	BPSS2344	102.64	108.7	118.6	245.55	206.3	241.03	-1.0704	DOWN	7.97E-04
BPSS0150	BPSS0150	12.71	11.01	12.23	29.12	20.64	25.92	-1.07392	DOWN	6.31E-03
BPSS0573	BPSS0573	17.92	15.61	19.59	40.44	41.12	30.85	-1.08144	DOWN	4.90E-03
BPSS1825	BPSS1825	171.5	159.22	174.82	351.49	343.55	375.12	-1.08193	DOWN	5.92E-05
BPSS0986	BPSS0986	11.43	11.71	9.77	25.17	21.92	22.58	-1.08201	DOWN	4.57E-04
BPSS1745	BPSS1745	17.37	15.3	18.2	31.45	30.65	45.76	-1.08427	DOWN	1.89E-02
BPSS2291	BPSS2291	11.81	11.16	12.46	29.53	23.9	21.76	-1.08557	DOWN	4.85E-03
BPSL2694	BPSL2694	131.88	120.62	130.62	267.92	263.14	282.02	-1.0856	DOWN	2.81E-05
BPSS1484	BPSS1484	45.97	45.8	44.8	78.77	79.97	131.2	-1.08611	DOWN	4.16E-02
BPSS0450	BPSS0450	7.67	7	7.45	16.07	13.36	17.55	-1.0867	DOWN	2.63E-03
BPSL3371	eutC	11.29	11.06	11.37	26.4	20.4	24.96	-1.08958	DOWN	2.19E-03
BPSL2339	BPSL2339	53.25	52.63	61.8	151.77	97.09	109.05	-1.09389	DOWN	1.98E-02
BPSS0159	BPSS0159	6.92	7.65	8.55	16.58	18.82	14.14	-1.09945	DOWN	3.54E-03
BPSS1241	BPSS1241	33.68	32.12	36.44	80.49	73.62	65.17	-1.10081	DOWN	1.07E-03
BPSL1312	glmS	5.82	6.18	6.78	15.42	13.5	11.38	-1.10158	DOWN	3.93E-03

BPSS1819	BPSS1819	29.54	26.14	27.03	60.54	61.07	55.91	-1.10185	DOWN	8.13E-05
BPSL2214	BPSL2214	80.53	72.61	74.45	141.55	162.95	184.37	-1.10301	DOWN	2.29E-03
BPSS1182	BPSS1182	97.39	101.46	73.32	156.88	211.86	216.92	-1.10556	DOWN	7.79E-03
BPSS1421	BPSS1421	23.17	17.98	19.93	40.46	48.07	42.95	-1.10607	DOWN	9.70E-04
BPSL0919A	BPSL0919A	64.77	62.62	70.53	147.68	126.96	151.44	-1.10621	DOWN	6.73E-04
BPSS1872	BPSS1872	22.26	21.95	26.11	57.13	45.38	49.02	-1.1076	DOWN	1.90E-03
BPSL1924	BPSL1924	41.38	41.08	45.18	83.74	90.2	101.33	-1.10877	DOWN	7.51E-04
BPSS0666	BPSS0666	6.24	7.6	7.18	15.88	18.45	11.01	-1.10902	DOWN	2.17E-02
BPSS2171	BPSS2171	28.19	26.22	25.33	73.98	51.82	46.73	-1.11347	DOWN	2.12E-02
BPSS0182	BPSS0182	5.59	5.52	6.58	13.4	11.21	13.67	-1.11366	DOWN	1.28E-03
BPSS2264	BPSS2264	20.48	17.9	18.45	49.3	34.39	39.5	-1.11616	DOWN	7.61E-03
BPSL0451	BPSL0451	164.26	151.98	134.22	324.07	320.11	333.66	-1.1182	DOWN	5.24E-05
BPSL1613	BPSL1613	11.74	12.72	11.66	29.83	21.98	26.66	-1.11934	DOWN	3.60E-03
BPSL2639	BPSL2639	38.84	34.29	34.45	87.69	82.75	63.51	-1.12079	DOWN	5.00E-03
BPSS0409	BPSS0409	5.23	5.98	6.48	13.39	12.6	12.49	-1.12117	DOWN	1.14E-04
BPSL2142	BPSL2142	5.85	5.49	5.65	15.11	13.62	8.23	-1.12128	DOWN	3.35E-02
BPSS0370	murI	23.98	23.87	29.57	68.02	54.2	46.25	-1.12171	DOWN	1.02E-02
BPSS1242	nirD	105.78	107.03	100.17	255.58	236.46	189.87	-1.12351	DOWN	3.31E-03
BPSL2971	BPSL2971	10.49	9.54	11.38	19.02	20.05	29.77	-1.13202	DOWN	2.28E-02
BPSS1045	BPSS1045	9.42	8.07	8.18	17.16	23.65	15.53	-1.13408	DOWN	1.53E-02
BPSL2437	BPSL2437	110.56	107.43	119	237.35	219.3	284	-1.13609	DOWN	2.35E-03
BPSS0635	BPSS0635	13.36	13.94	14.06	37.1	30.04	24.08	-1.14111	DOWN	1.16E-02
BPSL0253	BPSL0253	85.34	80.74	97.38	209.84	197.23	174.01	-1.14115	DOWN	8.01E-04
BPSS0184	BPSS0184	11.8	11.71	14.32	26.46	24.8	32.41	-1.14518	DOWN	3.44E-03
BPSS1339	BPSS1339	25.7	22.52	31.13	54.15	64.07	58.35	-1.15394	DOWN	1.06E-03
BPSL2190	BPSL2190	10.09	11.22	14.55	28.31	23.89	27.62	-1.15437	DOWN	1.58E-03

BPSS1469	BPSS1469	7.23	8.35	7.21	18.99	15.77	16.04	-1.15643	DOWN	1.05E-03
BPSL0285	BPSL0285	12.69	12.47	16.33	33.97	31.85	26.67	-1.15653	DOWN	2.46E-03
BPSS1467	BPSS1467	8.92	9.54	10.07	18.97	25.61	19.06	-1.15745	DOWN	6.24E-03
BPSS0431	BPSS0431	8.62	7.39	8.1	21.26	16.29	16.34	-1.16039	DOWN	4.16E-03
BPSL2700	BPSL2700	71.33	65.73	72.44	167.94	157.61	142.95	-1.1611	DOWN	3.32E-04
BPSS0420	rfbG	100.35	104.38	123.83	276.98	248.37	211.62	-1.16545	DOWN	2.55E-03
BPSL2417	BPSL2417	7.42	7.94	8.8	21.23	14.65	18.36	-1.16674	DOWN	6.74E-03
BPSL0248	BPSL0248	6.84	6.92	7.47	18.01	14.79	14.87	-1.16698	DOWN	1.22E-03
BPSS0275	pcaF	7.63	6.65	5.88	11.07	14.81	19.48	-1.16993	DOWN	2.78E-02
BPSS0712	BPSS0712	215.79	234.88	262.82	537.67	521.51	547.05	-1.17071	DOWN	4.41E-05
BPSS2178	BPSS2178	7.39	6.86	9.35	16.65	19.96	16.53	-1.17101	DOWN	1.91E-03
BPSL1182	BPSL1182	51.23	42.65	53.28	132.62	114.21	84.85	-1.17241	DOWN	1.26E-02
BPSS0719	BPSS0719	8.51	7.66	7.95	20.06	17.68	16.81	-1.17735	DOWN	5.38E-04
BPSL1307	BPSL1307	28.87	22.59	24.72	62.6	65.02	45.57	-1.18487	DOWN	7.18E-03
BPSL1615	BPSL1615	16.68	16.91	16.27	40.71	34.15	38.6	-1.18623	DOWN	4.00E-04
BPSL1541	BPSL1541	96.74	104.12	120.52	258.78	239.28	233.57	-1.18683	DOWN	1.91E-04
BPSL0686	BPSL0686	6.28	8.3	8.94	20	12.32	21.35	-1.19023	DOWN	2.64E-02
BPSL0719	BPSL0719	25.68	21.7	25.42	50.28	51.73	64.12	-1.1903	DOWN	2.45E-03
BPSL1289	BPSL1289	6.36	9.26	7.32	18.87	16.22	17.26	-1.19032	DOWN	1.04E-03
BPSL2073	BPSL2073	20.48	26.09	24.61	65.46	59.83	37.4	-1.19258	DOWN	2.51E-02
BPSS2268	BPSS2268	13.66	12.31	16.29	34.4	26.84	35.43	-1.19378	DOWN	3.55E-03
BPSL3162	ugpQ	27.6	25.89	30.44	72.54	59.8	59.7	-1.19415	DOWN	1.28E-03
BPSS0453	BPSS0453	35.62	40.74	40.18	78.65	83.62	105.15	-1.19828	DOWN	3.73E-03
BPSL1046	BPSL1046	37.14	38.51	43.16	81.49	87.6	103.58	-1.1985	DOWN	1.69E-03
BPSL2234	BPSL2234	38.89	38.13	44.93	103.89	96.96	79.13	-1.19903	DOWN	2.37E-03
BPSL3163	BPSL3163	27.4	25.85	29.33	70.37	61.79	57.98	-1.2032	DOWN	7.03E-04

BPSS0006	tdh	112.59	153.39	147.7	245.2	291.85	416.77	-1.2052	DOWN	2.70E-02
BPSS0930	BPSS0930	17.98	16.82	16.18	49.92	32.56	36.04	-1.21713	DOWN	1.34E-02
BPSLt35	BPSLt35	245.93	325.04	421.67	762.09	733.09	820.9	-1.22234	DOWN	1.50E-03
BPSL2786	BPSL2786	31.28	27.73	30.25	72.94	67.7	68.34	-1.22728	DOWN	3.43E-05
BPSS2309	BPSS2309	14.06	14.26	13.58	39.18	24.56	34.42	-1.22819	DOWN	1.21E-02
BPSL3095	BPSL3095	14.79	16.89	18.8	47.98	38.18	32.11	-1.2283	DOWN	9.03E-03
BPSL3287	BPSL3287	22.54	21.5	23.44	64.93	46.25	46.92	-1.22831	DOWN	7.95E-03
BPSL1170	kdpF	22.59	22.67	19.42	61.89	50.84	38.87	-1.22888	DOWN	1.26E-02
BPSS2293	BPSS2293	9.19	10.13	10.29	25.58	23.14	20.82	-1.23176	DOWN	7.15E-04
BPSL1390	BPSL1390	63.69	70.1	67.22	133.3	168.5	170.41	-1.23216	DOWN	1.77E-03
BPSS0278	BPSS0278	7.77	8.99	8.56	19.95	19.09	20.84	-1.2418	DOWN	4.90E-05
BPSL1465	BPSL1465	33.07	33.32	38.35	98.13	80.68	69.48	-1.24521	DOWN	4.92E-03
BPSS0667	BPSS0667	9.26	7.01	7.91	16.3	18	23.21	-1.25	DOWN	6.99E-03
BPSL0707	BPSL0707	37.62	37.33	39.1	105.19	92.63	73.53	-1.25049	DOWN	4.72E-03
BPSL3327	BPSL3327	7.55	5.27	5.69	19.74	12.84	11.59	-1.25476	DOWN	3.13E-02
BPSL2717	BPSL2717	116.59	126.57	124.84	280.06	298.85	299.97	-1.25596	DOWN	1.85E-05
BPSLt16	BPSLt16	18.69	20.81	18.72	48.78	41.39	49.17	-1.25902	DOWN	5.02E-04
BPSS0030	BPSS0030	7.88	6.19	7.09	18.45	14.6	17.66	-1.26093	DOWN	1.50E-03
BPSS0713	egtB	39.05	39.04	38.68	91.03	83.36	105.49	-1.26114	DOWN	1.11E-03
BPSL1044	otsA	68.85	70.03	70.66	200.51	170.73	131.59	-1.26285	DOWN	8.06E-03
BPSL3256	BPSL3256	15.36	17.48	14.04	36.95	39.81	35.8	-1.26365	DOWN	1.49E-04
BPSS0025	BPSS0025	9.74	11.83	12.05	30.3	26.89	23.55	-1.26396	DOWN	1.66E-03
BPSS1372	BPSS1372	44.09	36.46	38.36	82.17	91.36	112.14	-1.26448	DOWN	3.72E-03
BPSL1273	BPSL1273	194.97	183.6	160.38	415.99	494.75	387.35	-1.26817	DOWN	1.68E-03
BPSL2834	recN	33.48	32.14	38.54	104.49	79.97	66.43	-1.26825	DOWN	1.24E-02
BPSL2390	BPSL2390	32.36	37.42	36.15	97.19	61.92	96.27	-1.26953	DOWN	1.31E-02

BPSL2589	BPSL2589	7.26	7.26	8.65	21.18	19.4	15.46	-1.2742	DOWN	3.34E-03
BPSL1735	BPSL1735	37.23	33.13	35.51	96.46	77.54	82.23	-1.27515	DOWN	9.94E-04
BPSL1055	BPSL1055	10.07	9.15	8.1	25	21.59	19.61	-1.27687	DOWN	1.50E-03
BPSL3274	BPSL3274	15.81	18.89	19.69	46.12	34.03	51.95	-1.28022	DOWN	8.71E-03
BPSL2224	BPSL2224	105.52	93.07	94.35	231.91	218.29	261.63	-1.28093	DOWN	4.78E-04
BPSL1057	BPSL1057	21.73	18.87	19.97	43.01	37.36	66.83	-1.2811	DOWN	3.34E-02
BPSS0021	BPSS0021	10.15	11.53	12.49	34.99	27.59	20.74	-1.28593	DOWN	1.71E-02
BPSS0060B	BPSS0060B	33.09	34.78	35.29	97.32	91.17	64.68	-1.29522	DOWN	7.58E-03
BPSL0597	BPSL0597	31.96	31.72	33.35	96.18	74.59	67.51	-1.29616	DOWN	5.50E-03
BPSL1043	BPSL1043	24.55	28.3	26.62	72.06	61.87	63.2	-1.31067	DOWN	3.14E-04
BPSS1184	BPSS1184	24.41	21.02	20.62	57.05	54.5	53.15	-1.31821	DOWN	3.82E-05
BPSS0179	tssG	7.05	6.85	6.91	17.83	15.71	18.37	-1.31874	DOWN	2.19E-04
BPSS1782	BPSS1782	499.35	501.75	535.24	1232.33	1165.77	1450.75	-1.32493	DOWN	8.90E-04
BPSL0853	BPSL0853	160.39	167.48	166.8	480.73	435.88	327.58	-1.33067	DOWN	5.37E-03
BPSL0729	tssF	7.81	8.01	10.38	27.7	21.21	17.24	-1.33617	DOWN	1.35E-02
BPSL0020	BPSL0020	17.12	16.73	13.75	32.75	44.36	43.26	-1.33844	DOWN	3.24E-03
BPSS2343	BPSS2343	54.56	55.31	57.31	151.03	123.27	148.71	-1.33929	DOWN	6.72E-04
BPSLt20	BPSLt20	7.79	13.67	11.8	24.02	31.77	28.4	-1.33986	DOWN	3.91E-03
BPSL3386	BPSL3386	11.29	10.27	9.15	20.89	25.86	31.4	-1.34754	DOWN	6.96E-03
BPSL1833	BPSL1833	29.5	30.17	32.95	98.32	63.83	73.64	-1.34811	DOWN	9.83E-03
BPSS0280	gabD	44.64	40.78	39.19	134.32	96.64	86.41	-1.34875	DOWN	1.18E-02
BPSS0711	alr	57.31	47.38	53	153.48	134.3	116.6	-1.35862	DOWN	1.73E-03
BPSL0889	BPSL0889	11.29	11.42	11.7	29.09	26.74	32.64	-1.36236	DOWN	4.68E-04
BPSS2206	BPSS2206	95.02	83.4	85.76	203.66	198.57	279.21	-1.36707	DOWN	6.15E-03
BPSL1370	BPSL1370	17.17	19.02	20.43	55.9	52.58	37.63	-1.36767	DOWN	6.36E-03
BPSL3299	cheZ	52.32	52.31	57.22	165.14	119.14	134.56	-1.37174	DOWN	3.26E-03

BPSS1859	BPSS1859	7.08	6.79	7.22	24.24	17.1	13.49	-1.37841	DOWN	2.36E-02
BPSL0033	BPSL0033	33.8	31.06	30.46	90.84	73.76	83.29	-1.37885	DOWN	5.46E-04
BPSL0021	BPSL0021	19.46	23.28	22.19	51.46	52.59	64.85	-1.37921	DOWN	1.45E-03
BPSL1305	BPSL1305	15.49	17.25	18.7	52.23	41.82	39.96	-1.38138	DOWN	2.19E-03
BPSS0748	BPSS0748	25.86	22.74	27.89	66.64	68.05	64.95	-1.38406	DOWN	1.94E-05
BPSS0646	BPSS0646	10.88	12.73	10.72	36.97	30.92	21.74	-1.38451	DOWN	1.46E-02
BPSLt27	BPSLt27	9.24	9.48	11.03	24.22	19.84	33.65	-1.38521	DOWN	1.77E-02
BPSL2388	astA	20.91	18.85	22.06	58.76	39.93	63.53	-1.39181	DOWN	9.98E-03
BPSL2341	BPSL2341	52.09	49.72	46.51	160.67	118.21	113.02	-1.40177	DOWN	5.89E-03
BPSL0216	BPSL0216	179.58	165.07	144.1	341.93	506.85	443.18	-1.40239	DOWN	5.50E-03
BPSL0419	BPSL0419	11.44	9.62	10.5	29.5	28.73	25.36	-1.40523	DOWN	2.28E-04
BPSS0427	BPSS0427	105.73	109.01	102.43	274.04	263.02	306.89	-1.4119	DOWN	1.91E-04
BPSL1172	kdpB	21.85	21.7	25.05	64.4	61.61	56.7	-1.41328	DOWN	1.09E-04
BPSL1445	BPSL1445	6978.35	6697.95	7450.36	19901.82	18001.22	18610.72	-1.41954	DOWN	3.99E-05
BPSS1417	BPSS1417	7.49	8.08	6.62	19.63	16.38	23.56	-1.42468	DOWN	4.18E-03
BPSL2231	BPSL2231	60.44	51.13	61.16	171.88	145.96	145.87	-1.4247	DOWN	4.65E-04
BPSS1873	BPSS1873	14.26	14.15	16.46	46.24	36.23	38.09	-1.42593	DOWN	1.34E-03
BPSS0126	BPSS0126	7.49	5.91	6.92	15.11	16.26	24.26	-1.45296	DOWN	1.56E-02
BPSS0441	BPSS0441	5.55	6.39	6.95	19.32	17.71	14.72	-1.45394	DOWN	1.47E-03
BPSS2310	BPSS2310	10.81	9.98	11.74	35.27	26.53	27.32	-1.45398	DOWN	2.66E-03
BPSS0152	BPSS0152	27.19	32.67	33.96	103.39	74.47	79.38	-1.45515	DOWN	4.03E-03
BPSL0279	BPSL0279	32.4	33.22	36.06	108.41	89.75	80.82	-1.45613	DOWN	1.97E-03
BPSS0177	tssE	5.88	4.92	5.6	17.29	13.44	14.29	-1.45687	DOWN	1.36E-03
BPSL1132	BPSL1132	5.07	5.98	6.38	12.79	16.58	18.65	-1.46206	DOWN	4.40E-03
BPSS1166	BPSS1166	17.52	16.05	14.95	37.63	40.53	55.73	-1.4644	DOWN	7.35E-03
BPSL3238	BPSL3238	17.99	17.96	20.73	52.34	47.23	57.11	-1.46691	DOWN	3.72E-04

BPSS1799	BPSS1799	348.57	315.32	317.66	790.52	890.53	1033.61	-1.46764	DOWN	1.26E-03
BPSL3317	BPSL3317	57.63	53.17	56.68	184.05	136.88	142.78	-1.46923	DOWN	2.69E-03
BPSL2701	BPSL2701	89.56	81.08	72.5	254.9	247.02	171.81	-1.47038	DOWN	5.97E-03
BPSL1171	kdpA	36.18	28.22	33.57	103.48	96.25	72.11	-1.47235	DOWN	4.05E-03
BPSS0766	BPSS0766	86.24	81.51	93.29	245.56	206.27	274.22	-1.4758	DOWN	1.49E-03
BPSS1817	BPSS1817	20.46	21.88	28.43	83.52	63.45	50.08	-1.47735	DOWN	1.37E-02
BPSL0846	kynA	30.12	28.61	29.49	83.82	83.09	79.49	-1.48182	DOWN	3.04E-06
BPSLt17	BPSLt17	274.51	418.46	603.15	1308.32	969.47	1351.27	-1.4854	DOWN	7.18E-03
BPSS1173	BPSS1173	34.3	32.55	31.51	96.79	77.74	102.2	-1.49234	DOWN	1.34E-03
BPSS1172	BPSS1172	43.48	40.28	37.21	108.99	98.04	133.41	-1.49275	DOWN	2.32E-03
BPSS0985	BPSS0985	10.26	11.85	11.01	35.92	29.61	27.76	-1.49402	DOWN	1.33E-03
BPSS1340	BPSS1340	14.21	13.31	11.8	38.78	37.73	34.3	-1.49475	DOWN	9.78E-05
BPSL3296	BPSL3296	22.92	24.49	21.98	79.77	54.88	62.18	-1.50415	DOWN	4.61E-03
BPSL1829	BPSL1829	7.97	7.72	9.24	28.82	19.69	22.46	-1.50933	DOWN	5.01E-03
BPSS0679	BPSS0679	10.78	12.1	12.33	40.06	35.21	25.54	-1.51758	DOWN	7.03E-03
BPSS1158	narH	5.6	4.77	5.55	15.42	14.12	16.07	-1.51851	DOWN	9.78E-05
BPSS2342	BPSS2342	31.63	27.92	33.1	96.32	82.34	87.68	-1.52341	DOWN	1.85E-04
BPSS1174	BPSS1174	30.8	28.66	28.19	101.84	71.39	79.32	-1.52674	DOWN	3.87E-03
BPSS2205	BPSS2205	89.44	79.82	102.08	266.73	220.77	295.17	-1.5283	DOWN	1.66E-03
BPSS1171	BPSS1171	38.41	35.81	33.65	96.44	92.74	122.4	-1.53031	DOWN	1.97E-03
BPSS1168	BPSS1168	28.43	25.05	25.99	73.81	69.48	86.97	-1.53478	DOWN	7.18E-04
BPSL2989	BPSL2989	512.07	544.83	698.66	1681.16	1548.45	1880.15	-1.54132	DOWN	5.71E-04
BPSS0915	BPSS0915	30.16	32.22	32.18	91.56	88.55	96.99	-1.5511	DOWN	1.86E-05
BPSS2158	BPSS2158	7.27	7.21	8.69	32.2	19.64	16.31	-1.55646	DOWN	3.68E-02
BPSS0180	BPSS0180	5.14	4.83	5.28	16.78	13.3	14.78	-1.55662	DOWN	6.31E-04
BPSL2389	aruF	21.17	19.81	23.14	74.93	44.65	69.7	-1.56168	DOWN	1.13E-02

BPSS1181	BPSS1181	36.42	31.12	32.19	112.22	84.81	97.49	-1.56227	DOWN	1.30E-03
BPSS0175	BPSS0175	5.12	4.45	4.31	17.52	14.22	9.36	-1.56613	DOWN	1.90E-02
BPSLt19	BPSLt19	6.16	8.42	10.57	17.12	21.73	36	-1.57344	DOWN	4.66E-02
BPSL2591	BPSL2591	44.61	42.24	47.02	154.27	131.57	112.94	-1.57476	DOWN	1.83E-03
BPSL0398	BPSL0398	11.96	13.71	16.82	49.13	38.8	39.29	-1.58213	DOWN	1.51E-03
BPSL2233	cysC	140.46	132.42	156.37	455.4	384.67	447.99	-1.58531	DOWN	2.62E-04
BPSS0718	BPSS0718	21.18	18.47	21.96	77.48	57.48	50.06	-1.58644	DOWN	7.58E-03
BPSL3314	aqpZ	175.77	183.83	162.22	664.74	547.63	355.38	-1.58707	DOWN	1.82E-02
BPSS0700	BPSS0700	12.59	13.21	15.54	43.8	37.92	43.16	-1.59493	DOWN	1.76E-04
BPSL1591	BPSL1591	416.01	392.67	456.89	1517.21	1200.21	1122.81	-1.60141	DOWN	2.16E-03
BPSS1454	BPSS1454	13.73	15.92	14.52	50.31	40.02	44.23	-1.60711	DOWN	5.93E-04
BPSL3297	BPSL3297	100.9	107.99	117.56	444.34	291.05	268.91	-1.62126	DOWN	1.51E-02
BPSL2387	astD	28.37	25.7	27.09	101.84	64.74	84.25	-1.62787	DOWN	6.23E-03
BPSS1175	BPSS1175	15.02	15.04	15.14	52.47	36.86	50.39	-1.62814	DOWN	2.99E-03
BPSLt31	BPSLt31	171.61	236.85	335.44	915.96	612.08	772.23	-1.62862	DOWN	6.54E-03
BPSS1416	BPSS1416	6.01	6.7	4.7	19.22	14.06	20.65	-1.63117	DOWN	4.29E-03
BPSS1460	BPSS1460	7.06	6.79	6.66	24.31	21.31	18.05	-1.63429	DOWN	1.36E-03
BPSL1134	plcR	21.21	20.66	22.76	74.27	68.07	58.49	-1.6357	DOWN	6.08E-04
BPSS2224	BPSS2224	8.21	5.92	7.63	23.6	16.9	27.19	-1.63726	DOWN	7.76E-03
BPSL2650	BPSL2650	14.91	13.77	15.21	57.25	49.42	29.93	-1.63799	DOWN	1.91E-02
BPSL2232	BPSL2232	65.65	65.87	74.34	254.63	211.99	175.5	-1.64118	DOWN	3.22E-03
BPSS0276	BPSS0276	124.89	114.2	106.91	473.65	288.49	317.62	-1.64187	DOWN	1.33E-02
BPSL1485	BPSL1485	48.69	46.26	43.44	180.76	161.73	90.32	-1.64499	DOWN	2.36E-02
BPSL1042	BPSL1042	11.96	10.98	14.32	43.57	36.87	36.13	-1.6455	DOWN	5.00E-04
BPSS1453	BPSS1453	19.92	20.54	23.76	66.47	63.23	71.55	-1.64789	DOWN	7.14E-05
BPSL2093	BPSL2093	40.16	52.29	47.33	136.9	133.36	169.8	-1.65454	DOWN	1.17E-03

BPSS2230	BPSS2230	12.39	9.67	10.94	49.47	25.73	28.9	-1.65743	DOWN	3.39E-02
BPSL0694	BPSL0694	78.57	70.88	76.26	257.17	209.22	246.56	-1.65933	DOWN	3.83E-04
BPSS2270	lpdA	31.92	37.21	39.86	140.67	101.97	103.57	-1.66745	DOWN	3.54E-03
BPSL2011	BPSL2011	7.06	8.33	11.07	22.91	22.58	38.66	-1.66915	DOWN	2.41E-02
BPSL0268	flgM	49.2	55.54	56.86	249.31	134.73	131.34	-1.67321	DOWN	3.86E-02
BPSS1455	BPSS1455	9.71	12.66	13.05	38.97	29.8	44.51	-1.67726	DOWN	4.19E-03
BPSL2394	hisQ	8.05	11.91	9.54	33.6	24.25	36.77	-1.68143	DOWN	5.21E-03
BPSS0337	BPSS0337	10.54	13.31	8.92	38.35	33.67	33.7	-1.6898	DOWN	2.70E-04
BPSL0706	BPSL0706	6.01	5.92	5.78	22.98	16.61	17.89	-1.6985	DOWN	2.43E-03
BPSL1045	BPSL1045	40.84	36.35	37.7	140.24	122.44	111.72	-1.70433	DOWN	5.07E-04
BPSS1019	BPSS1019	17.44	15.33	15.82	68.46	50.81	41.11	-1.72276	DOWN	9.73E-03
BPSL3300	cheY	58.86	63.95	59.64	247.73	176.71	188.83	-1.74902	DOWN	2.84E-03
BPSL3316	BPSL3316	15.78	13.57	16.26	62.35	42.71	48.35	-1.74997	DOWN	3.67E-03
BPSL2599	BPSL2599	318.28	306.22	282.74	1048.24	982.95	1035.4	-1.75708	DOWN	5.72E-06
BPSL2367	BPSL2367	18.42	16.5	17.48	69.97	47.08	60.35	-1.75937	DOWN	3.33E-03
BPSS2246	BPSS2246	38.92	36.2	45.68	152.56	115.49	143.46	-1.76831	DOWN	1.09E-03
BPSS0014	BPSS0014	63.25	62.24	64.29	238.32	198.52	213.6	-1.77709	DOWN	1.89E-04
BPSL1174	BPSL1174	26.23	25.87	28.3	92.96	88.02	96.75	-1.78842	DOWN	1.54E-05
BPSS0725	BPSS0725	26.97	23.77	22.27	105.08	77.18	70.64	-1.7924	DOWN	4.90E-03
BPSLt38	BPSLt38	84.88	50.49	85.21	213.41	194.65	358.89	-1.79783	DOWN	2.66E-02
BPSS1816	BPSS1816	14.9	14.25	21.09	78.71	61.31	36.88	-1.81603	DOWN	2.67E-02
BPSS2139	BPSS2139	9.64	9.16	9.88	46.29	33.79	20.95	-1.81667	DOWN	3.01E-02
BPSL0730	BPSL0730	6.42	7.28	8.01	31.12	23.15	23.44	-1.83974	DOWN	2.14E-03
BPSLt32	BPSLt32	35.66	33.43	42.86	122.27	144.05	136.37	-1.84682	DOWN	1.56E-04
BPSLt36	BPSLt36	40.48	49.14	53.34	142.39	150.2	222.25	-1.84851	DOWN	8.50E-03
BPSS1863	BPSS1863	6.64	5.88	8.46	24.15	20.4	31.03	-1.84899	DOWN	4.75E-03

BPSS2138	BPSS2138	18.48	17.09	17.31	87.82	63.63	40.35	-1.85881	DOWN	2.79E-02
BPSS1170	BPSS1170	27.32	24.13	23.7	81.39	76.19	117.49	-1.87195	DOWN	6.93E-03
BPSS1156	narI	7.43	7.7	8.75	27.73	25.79	34.36	-1.87973	DOWN	1.25E-03
BPSS1177	BPSS1177	8.8	8.28	10.28	42.98	28.19	29.57	-1.8805	DOWN	6.77E-03
BPSL2727	xdhB	5.26	5.83	6.95	16.72	18.48	31.43	-1.88497	DOWN	2.55E-02
BPSL1568	BPSL1568	41.29	55.75	46.38	161.43	150.86	218.18	-1.88702	DOWN	3.77E-03
BPSL1173	kdpC	17.92	14.91	18.19	65.36	59.87	64.92	-1.898	DOWN	2.27E-05
BPSL2529	BPSL2529	7.1	6.77	5.63	27.59	25.55	19.78	-1.90284	DOWN	1.71E-03
BPSL2702	BPSL2702	103.18	92.65	101.24	386.66	395.85	329.55	-1.90436	DOWN	2.06E-04
BPSL3321	fliT	42.59	50.63	48.78	216.6	164.19	159.34	-1.92742	DOWN	2.00E-03
BPSL3307	BPSL3307	45.7	50.93	43.22	223.39	161.71	150.56	-1.93744	DOWN	4.40E-03
BPSL1567	BPSL1567	23.96	29.46	27.11	98.51	100.31	110.67	-1.94229	DOWN	4.95E-05
BPSL2386	astB	30.27	31.08	32.05	149.64	84.38	127.43	-1.9523	DOWN	9.57E-03
BPSS1415	BPSS1415	7.83	6.7	5.8	24.9	20.29	33.55	-1.95349	DOWN	7.74E-03
BPSL2530	fdhF	7.9	8.32	8.69	37.29	30.88	28.45	-1.9556	DOWN	8.32E-04
BPSS1589	BPSS1589	34.47	33.47	36.91	146	130.91	130.59	-1.95847	DOWN	4.12E-05
BPSS0176	BPSS0176	6.28	5.09	5.46	26.1	20.3	19.34	-1.96574	DOWN	1.60E-03
BPSL3308	motB	25.5	28.22	35.2	141.72	91.26	115.51	-1.97054	DOWN	4.33E-03
BPSL1989	iolG	19.26	20.67	21.35	77.64	74.45	88.55	-1.97339	DOWN	1.57E-04
BPSL3298	BPSL3298	108.55	108.92	113.32	565.31	372.87	363.57	-1.97646	DOWN	7.92E-03
BPSS2271	BPSS2271	49.37	53.76	59.97	272.33	196.11	174.75	-1.97949	DOWN	5.79E-03
BPSS0027	BPSS0027	5.22	4.81	5.28	22.5	16.01	21.95	-1.98151	DOWN	1.95E-03
BPSS1178	BPSS1178	9.62	8.94	10	45.69	30.86	36.6	-1.98617	DOWN	2.87E-03
BPSS0633	BPSS0633	31.36	34.29	34.98	157.87	126.73	116.55	-1.99508	DOWN	1.31E-03
BPSL1746	BPSL1746	13.33	10.81	12.6	61.68	49.09	36.53	-2.00333	DOWN	7.23E-03
BPSS0619	BPSS0619	247.45	227.56	216.12	707.55	1162.39	911.25	-2.00867	DOWN	6.15E-03

BPSS0949	BPSS0949	31.96	27.11	27.82	158.85	109.22	83.39	-2.0161	DOWN	1.65E-02
BPSS1961	BPSS1961	25.41	24.17	19.87	90.74	73.75	116.5	-2.01647	DOWN	4.92E-03
BPSL0267	BPSL0267	16.22	19.44	17.16	94.71	64.22	56.24	-2.02632	DOWN	1.00E-02
BPSL0701	BPSL0701	7.5	9.84	8.39	37.51	32.54	34.88	-2.0279	DOWN	7.69E-05
BPSL1988	BPSL1988	9.92	11.3	10.61	44.05	37	49.58	-2.03703	DOWN	8.46E-04
BPSL2465	BPSL2465	45.89	41.62	44.79	206.53	165.26	178.7	-2.0569	DOWN	3.37E-04
BPSS0620	mmsB	94.19	89.79	83.49	288.13	470.27	363.76	-2.06883	DOWN	5.76E-03
BPSL2919	BPSL2919	10.44	9.26	11.58	52.58	51.12	28.42	-2.07854	DOWN	1.28E-02
BPSL2925	BPSL2925	178.54	176.48	170.88	879.09	776.11	568.99	-2.08042	DOWN	3.43E-03
BPSL3389	putA	37.5	31.6	35.55	187.41	147.54	110.73	-2.09044	DOWN	6.89E-03
BPSL0356	BPSL0356	20.84	21.8	23.21	105.51	114.55	62.3	-2.10028	DOWN	1.11E-02
BPSL2728	xdhA	9.48	8.65	10.57	44.33	47.37	36.04	-2.15409	DOWN	6.52E-04
BPSS0008	BPSS0008	13.05	12.64	16.67	57.55	61.91	71.27	-2.17076	DOWN	3.10E-04
BPSL0422	BPSL0422	25.76	24	27.8	126.64	104.68	117.91	-2.17079	DOWN	1.52E-04
BPSL3303	BPSL3303	36.09	40.89	39.14	202.29	156.61	168.95	-2.18451	DOWN	5.61E-04
BPSS2204	BPSS2204	54.16	54.41	63.58	314	230.25	249.34	-2.20473	DOWN	1.25E-03
BPSL3309	motA	41.82	43.26	48.61	257.05	163.81	203.23	-2.22286	DOWN	3.81E-03
BPSL0320	BPSL0320	10.55	10.76	9.39	56.24	53.46	34.54	-2.23216	DOWN	5.19E-03
BPSL1976	BPSL1976	16.2	16.76	20.16	107.5	65.04	79.37	-2.24558	DOWN	6.14E-03
BPSS0914	BPSS0914	73.47	93.57	106.23	488.59	502.4	315.48	-2.25728	DOWN	4.81E-03
BPSL2110	BPSL2110	28.39	26.64	27.99	123.72	145.48	129.41	-2.26345	DOWN	8.72E-05
BPSL0465	BPSL0465	10.69	13.82	13.64	88.04	47.5	48.62	-2.2712	DOWN	2.20E-02
BPSS0281	BPSS0281	7.55	7.79	9.96	60.45	32.57	29.66	-2.27769	DOWN	3.00E-02
BPSL3302	cheD	45.22	44.86	48.77	278.98	197.64	198.97	-2.28262	DOWN	2.66E-03
BPSL2385	astE	17.84	16.27	19.02	100.2	63.42	96.7	-2.29269	DOWN	4.18E-03
BPSS0965	BPSS0965	20.91	19.44	21.15	99.07	107.06	98.27	-2.30731	DOWN	9.22E-06

BPSS0933	BPSS0933	46.08	49.59	57.14	315.9	209.63	258.24	-2.35869	DOWN	2.43E-03
BPSS2292	BPSS2292	5.36	5.47	5.17	34.03	25.65	24.4	-2.39369	DOWN	1.69E-03
BPSS0913	BPSS0913	185.04	186.12	207.09	1052.68	975.37	1015.29	-2.39589	DOWN	3.96E-06
BPSL0280	flgK	55.55	55.31	67.49	418.02	269.01	254.79	-2.40074	DOWN	8.27E-03
BPSS2137	BPSS2137	13.62	10.91	13.08	85.43	69.4	45.22	-2.41117	DOWN	9.87E-03
BPSL0704	BPSL0704	9.49	10.41	13.08	85.19	44.29	47.63	-2.42498	DOWN	2.17E-02
BPSL1747	BPSL1747	8.51	9.07	8.65	60.74	45.99	34.28	-2.42651	DOWN	7.51E-03
BPSS1447	BPSS1447	20.83	32.09	31.77	188.68	87.51	182.08	-2.43593	DOWN	1.93E-02
BPSL3306	cheA	51.66	52.84	64.6	412.01	258.73	249.19	-2.44365	DOWN	9.11E-03
BPSL3305	cheW	139.09	137.03	144.18	961.14	653.3	680.28	-2.44883	DOWN	3.16E-03
BPSL3301	BPSL3301	84.49	81.16	85.65	620.76	397.62	365.72	-2.46147	DOWN	9.27E-03
BPSL0421	BPSL0421	31.93	26.07	30.87	188.37	158.15	146.94	-2.47316	DOWN	4.19E-04
BPSL0232	fliS	49.61	46.66	47.21	360.05	210.7	229.81	-2.48016	DOWN	9.54E-03
BPSS1462	BPSS1462	5.34	4.33	5.74	30.85	33.42	22.39	-2.4915	DOWN	2.11E-03
BPSS1218	BPSS1218	5.17	6.56	8.02	37.74	27.81	47	-2.51064	DOWN	5.25E-03
BPSL0235	BPSL0235	7.99	8.93	12.47	77.65	43	48.7	-2.52661	DOWN	1.25E-02
BPSS1449	BPSS1449	24.58	34.41	32.03	171.86	108.78	248.75	-2.54008	DOWN	2.27E-02
BPSS0253	BPSS0253	8.7	8.4	9.09	72.38	47.68	32.32	-2.54059	DOWN	2.27E-02
BPSL3304	BPSL3304	70.03	68.62	72.74	582.2	335.31	357.96	-2.59305	DOWN	1.08E-02
BPSL2585	BPSL2585	22.71	25.9	25.88	169.05	198.25	89.01	-2.6149	DOWN	1.76E-02
BPSL1263	cdpA	8.53	8.23	8.47	73.03	41.17	41.24	-2.62315	DOWN	1.50E-02
BPSL0703	BPSL0703	6.22	6.96	7.44	60.86	32.24	34.42	-2.62861	DOWN	1.80E-02
BPSL1994	iolC	6.64	6.14	7.41	41.83	44.5	46.92	-2.72242	DOWN	1.55E-05
BPSS1906	BPSS1906	5.84	5.5	6.42	55.81	34.59	27.59	-2.73196	DOWN	1.70E-02
BPSL1993	BPSL1993	5.58	7.66	6.99	41.18	46	52.66	-2.78921	DOWN	2.97E-04
BPSL3320	fliD	44.72	46.09	49.59	450.47	297.23	240.08	-2.81465	DOWN	1.09E-02

BPSL0325	BPSL0325	5.12	4.76	8.39	50.96	34.08	43.83	-2.81837	DOWN	1.84E-03
BPSL0233	BPSL0233	45.58	46.29	51.54	465.3	283.18	303.86	-2.87538	DOWN	6.25E-03
BPSS0622	BPSS0622	76.65	59.82	68.35	409.8	572.69	527.68	-2.88228	DOWN	8.75E-04
BPSL0281	flgL	58.13	62.85	63.89	599.94	434.59	338.96	-2.89326	DOWN	6.53E-03
BPSL1731	BPSL1731	17.89	17.02	16.91	203.07	105.71	76.94	-2.89597	DOWN	4.34E-02
BPSL1990	BPSL1990	6.76	6.62	6.66	40.76	50.8	61.21	-2.93041	DOWN	1.70E-03
BPSL1997	iolB	5.9	5.65	5.33	45.39	43.38	40.02	-2.93163	DOWN	1.88E-05
BPSL2584	BPSL2584	25.93	28.3	24.11	232.32	239.74	140.32	-2.96661	DOWN	5.11E-03
BPSS0621	BPSS0621	62.3	49.77	48.41	320.48	493	446.27	-2.97267	DOWN	2.09E-03
BPSL3338	BPSL3338	10.26	10.78	13.08	129.83	70.14	72.97	-2.99989	DOWN	1.50E-02
BPSL1262	BPSL1262	9.5	10.42	12.34	128.77	74.44	76.82	-3.11776	DOWN	9.63E-03
BPSL1566	BPSL1566	15.4	14.64	14.72	130.51	143.19	127.47	-3.16393	DOWN	1.61E-05
BPSS1446	BPSS1446	10.39	16.78	16.62	182.04	67.75	161.45	-3.23131	DOWN	2.54E-02
BPSS1448	BPSS1448	67.24	88.86	93.49	956.51	508.39	1037.71	-3.3258	DOWN	1.04E-02
BPSS1445	BPSS1445	15.28	19.75	22.08	290.88	87.16	203.03	-3.3469	DOWN	4.16E-02
BPSS0259	BPSS0259	7.39	9.82	10.15	129.97	113.59	79.07	-3.55974	DOWN	2.81E-03
BPSS0260	BPSS0260	6.74	7.43	7.71	146.05	118.56	91.07	-4.0229	DOWN	2.18E-03
BPSS0258	BPSS0258	5.23	6.94	6.2	128.16	103.59	90.81	-4.13414	DOWN	7.62E-04
BPSS0261	dhaL	7.7	8.07	8.85	204.13	178.5	147.72	-4.42904	DOWN	4.95E-04
BPSS0731	BPSS0731	9.33	9.71	11.46	256.11	192.97	313.5	-4.64401	DOWN	2.18E-03
BPSS1892	catA	8.71	8.29	7.21	217.96	197.28	255.04	-4.79109	DOWN	2.19E-04
BPSS1891	BPSS1891	5.94	6.19	9.11	180.31	187.69	461.22	-5.2869	DOWN	4.35E-02
BPSS0011	BPSS0011	15.56	12.98	12.66	493.16	553.32	1023.19	-5.65061	DOWN	1.57E-02

a. “I” represents the intracellular *B. ps*, and “NI” represents the in vitro *B.ps*.

b. “FC” represents Fold change.
